# Improving rational use of ACTs through diagnosis-dependent subsidies: Evidence from a cluster-randomized controlled trial in western Kenya

**DOI:** 10.1371/journal.pmed.1002607

**Published:** 2018-07-17

**Authors:** Wendy Prudhomme O’Meara, Diana Menya, Jeremiah Laktabai, Alyssa Platt, Indrani Saran, Elisa Maffioli, Joseph Kipkoech, Manoj Mohanan, Elizabeth L. Turner

**Affiliations:** 1 Department of Medicine, Duke University School of Medicine, Durham, North Carolina, United States of America; 2 Duke Global Health Institute, Duke University, Durham, North Carolina, United States of America; 3 Moi University School of Public Health, College of Health Sciences, Eldoret, Kenya; 4 Moi University School of Medicine, College of Health Sciences, Eldoret, Kenya; 5 Academic Model Providing Access to Healthcare (AMPATH), Eldoret, Kenya; 6 Department of Biostatistics and Bioinformatics, Duke University, Durham, North Carolina, United States of America; 7 Department of Economics, Duke University, Durham, North Carolina, United States of America; 8 Sanford School of Public Policy, Duke University, Durham, North Carolina, United States of America; Mahidol-Oxford Tropical Medicine Research Unit, THAILAND

## Abstract

**Background:**

More than half of artemisinin combination therapies (ACTs) consumed globally are dispensed in the retail sector, where diagnostic testing is uncommon, leading to overconsumption and poor targeting. In many malaria-endemic countries, ACTs sold over the counter are available at heavily subsidized prices, further contributing to their misuse. Inappropriate use of ACTs can have serious implications for the spread of drug resistance and leads to poor outcomes for nonmalaria patients treated with incorrect drugs. We evaluated the public health impact of an innovative strategy that targets ACT subsidies to confirmed malaria cases by coupling free diagnostic testing with a diagnosis-dependent ACT subsidy.

**Methods and findings:**

We conducted a cluster-randomized controlled trial in 32 community clusters in western Kenya (population approximately 160,000). Eligible clusters had retail outlets selling ACTs and existing community health worker (CHW) programs and were randomly assigned 1:1 to control and intervention arms. In intervention areas, CHWs were available in their villages to perform malaria rapid diagnostic tests (RDTs) on demand for any individual >1 year of age experiencing a malaria-like illness. Malaria RDT-positive individuals received a voucher for a discount on a quality-assured ACT, redeemable at a participating retail medicine outlet. In control areas, CHWs offered a standard package of health education, prevention, and referral services. We conducted 4 population-based surveys—at baseline, 6 months, 12 months, and 18 months—of a random sample of households with fever in the last 4 weeks to evaluate predefined, individual-level outcomes. The primary outcome was uptake of malaria diagnostic testing at 12 months. The main secondary outcome was rational ACT use, defined as the proportion of ACTs used by test-positive individuals. Analyses followed the intention-to-treat principle using generalized estimating equations (GEEs) to account for clustering with prespecified adjustment for gender, age, education, and wealth. All descriptive statistics and regressions were weighted to account for sampling design. Between July 2015 and May 2017, 32,404 participants were tested for malaria, and 10,870 vouchers were issued. A total of 7,416 randomly selected participants with recent fever from all 32 clusters were surveyed. The majority of recent fevers were in children under 18 years (62.9%, *n =* 4,653). The gender of enrolled participants was balanced in children (49.8%, *n =* 2,318 boys versus 50.2%, *n =* 2,335 girls), but more adult women were enrolled than men (78.0%, *n =* 2,139 versus 22.0%, *n =* 604). At baseline, 67.6% (*n =* 1,362) of participants took an ACT for their illness, and 40.3% (*n =* 810) of all participants took an ACT purchased from a retail outlet. At 12 months, 50.5% (*n =* 454) in the intervention arm and 43.4% (*n =* 389) in the control arm had a malaria diagnostic test for their recent fever (adjusted risk difference [RD] = 9 percentage points [pp]; 95% CI 2–15 pp; *p* = 0.015; adjusted risk ratio [RR] = 1.20; 95% CI 1.05–1.38; *p* = 0.015). By 18 months, the ARR had increased to 1.25 (95% CI 1.09–1.44; *p* = 0.005). Rational use of ACTs in the intervention area increased from 41.7% (*n =* 279) at baseline to 59.6% (*n =* 403) and was 40% higher in the intervention arm at 18 months (ARR 1.40; 95% CI 1.19–1.64; *p* < 0.001). While intervention effects increased between 12 and 18 months, we were not able to estimate longer-term impact of the intervention and could not independently evaluate the effects of the free testing and the voucher on uptake of testing.

**Conclusions:**

Diagnosis-dependent ACT subsidies and community-based interventions that include the private sector can have an important impact on diagnostic testing and population-wide rational use of ACTs. Targeting of the ACT subsidy itself to those with a positive malaria diagnostic test may also improve sustainability and reduce the cost of retail-sector ACT subsidies.

**Trial registration:**

ClinicalTrials.gov NCT02461628

## Abstract 2

In a cluster randomized controlled trial, Wendy O'Meara and colleagues investigate whether providing vouchers for discounted medication at testing improved testing before treatment and correct use of artemisinin combination therapy in Kenya.

## Author summary

**Why was this study done?:**

- In most malaria-endemic countries, first-line antimalarials called artemisinin combination therapies, or ACTs, are available over the counter in retail medicine outlets and can be purchased without a diagnostic test.
- ACTs are heavily subsidized by government or international donors and are very inexpensive to the consumer. Approximately 40% of all subsidized ACTs are sold through the private sector, where it is estimated that 80% are taken by people without malaria.
- Misuse and overconsumption of ACTs has serious consequences; it can lead to delayed treatment for the true cause of illness and may contribute to the spread of antimalarial resistance. It also wastes public subsidy funds for patients who don’t need antimalarials.
- The use of a diagnostic test before treatment could improve the correct use of ACTs.

**What did the researchers do and find?:**

- We tested an innovative approach that created a partnership between community health workers (CHWs) and retail medicine outlets. Our intervention was specifically designed to reach individuals purchasing drugs over the counter and to incorporate the retail sector, which delivers the majority of ACTs in Kenya.
- The CHWs provided free malaria testing in the community using simple point-of-care malaria rapid diagnostic tests (RDTs) and issued a voucher to anyone with a positive test, which could be redeemed at a retail medicine outlet in exchange for a discounted ACT.
- The voucher allowed the ACT subsidy to be targeted only to patients with a confirmed malaria infection and created an incentive for patients to be tested by a CHW before buying a drug. Individuals with a negative test or without a test had to pay a higher price for retail ACTs.
- We tested the effect of this intervention on testing before treatment and correct use of ACTs in a cluster-randomized controlled trial. After 18 months, the intervention improved the proportion of fevers tested before treatment by 25% and improved the proportion of ACT dispensed to true malaria cases by 40% compared to the control arm.

**What do these findings mean?:**

- Making ACT subsidies conditional on the diagnostic test result could help ensure that the information from the test informs treatment.
- Community-based interventions that include the private sector can have an important impact on diagnostic testing and population-wide rational use of ACTs, as well as potentially improve care for the millions of suspected malaria cases seeking treatment in retail outlets.

## Introduction

Each year, half of the 215 million cases of malaria—and hundreds of millions of cases of nonmalaria febrile illnesses—seek care in the informal health sector [1]. In the mid-2000s, most malaria-endemic countries changed their first-line antimalarial to an artemisinin combination therapy (ACT) due to widespread resistance to older drugs. At the time, it was recognized that most malaria cases cared for in the retail sector were being inappropriately treated due to the high cost of new ACTs [2]. In response to this, the Affordable Medicines Facility-malaria (AMFm) piloted retail-sector ACT subsidies in 7 countries that were subsequently widely adopted. As a result, the market share of ACTs dramatically increased [3, 4]. By 2017, 10 years after retail-sector subsidies were introduced, 44% of quality-assured ACTs were distributed through the retail sector [5, 6].

There has been enormous global investment in publicly subsidized ACTs delivered through the private retail sector, but targeting of ACTs to those with a malaria diagnosis remains poor in this context. Malaria diagnostic testing is largely absent from the retail sector [6], and as a result, individuals without malaria consume 66% to 80% of ACTs sold over the counter [7, 8]. At the same time, up to 70% of individuals with malaria (but without information about their diagnosis) fail to get an ACT [9]. The discordance between who needs an ACT and who purchases one highlights the importance of improving access to diagnostic testing amongst those who seek care outside the formal health sector. Furthermore, inappropriate use of subsidized ACTs results in wastage of limited public funds.

There are compelling individual and public health benefits to improving targeting of ACTs. Malaria can quickly become life-threatening if inappropriately treated. The precipitous decline in malaria mortality has been attributed largely to expanding access to ACTs [10]. On the other hand, treating a patient’s nonmalaria illness with antimalarials leaves the true cause untreated, leading to poor outcomes and elevated mortality from nonmalarial illnesses [11, 12]. A critical public health concern is the spread of antimalarial resistance, which is accelerated by presumptive ACT use [13–16]. No immediate successors are available to replace artemisinin in the case of widespread resistance, which could lead to a marked increase in malaria-associated deaths [17, 18]. Finally, in an era of uncertain funding for global health, the sustainability of drug subsidy programs is being called into question. Several studies have shown that test-and-treat reduces program costs over presumptive treatment across a wide range of malaria prevalences if adherence to the test is high [19, 20]. Even when not strictly cost-saving, there is good evidence that it is cost-effective up to high prevalence levels [20–23]. The recent decline in the cost of rapid diagnostic tests (RDTs) makes testing even more affordable and cost-saving than ever before [24]. In summary, targeting subsidized ACTs to individuals with parasitologically confirmed malaria could significantly contribute to sustainability and cost-effectiveness of donor-funded subsidies as well as safeguard future efficacy of these key drugs.

Seventy percent of the population of Kenya is at risk for malaria [25]. Since 2003, the incidence of malaria has declined in many parts of Kenya, although progress has stagnated in the last few years [6]. Over the counter consumption of ACTs provided in the retail sector is high [25, 26]. We tested a strategy to improve targeting of ACT to individuals with confirmed malaria infection in western Kenya where two-thirds of families access care in the retail or informal health sector [25, 26]. Free, community-based testing using point-of-care malaria RDT was coupled with a diagnosis-dependent ACT subsidy. The subsidy, provided in the form of a voucher that could be redeemed at a retail medicine outlet, was conditional upon a positive malaria diagnostic test, and served both as an incentive to be tested as well as a mechanism to target the subsidy to individuals with confirmed infection. Our goal was to attract individuals who normally seek care in the retail sector to have a diagnostic test performed by a community health worker (CHW) before purchasing drugs. Our study provides new evidence on the potential to use conditional subsidies and community-level diagnostic testing to improve population-wide targeting and rational use of ACTs.

## Methods

**Fig 1:**
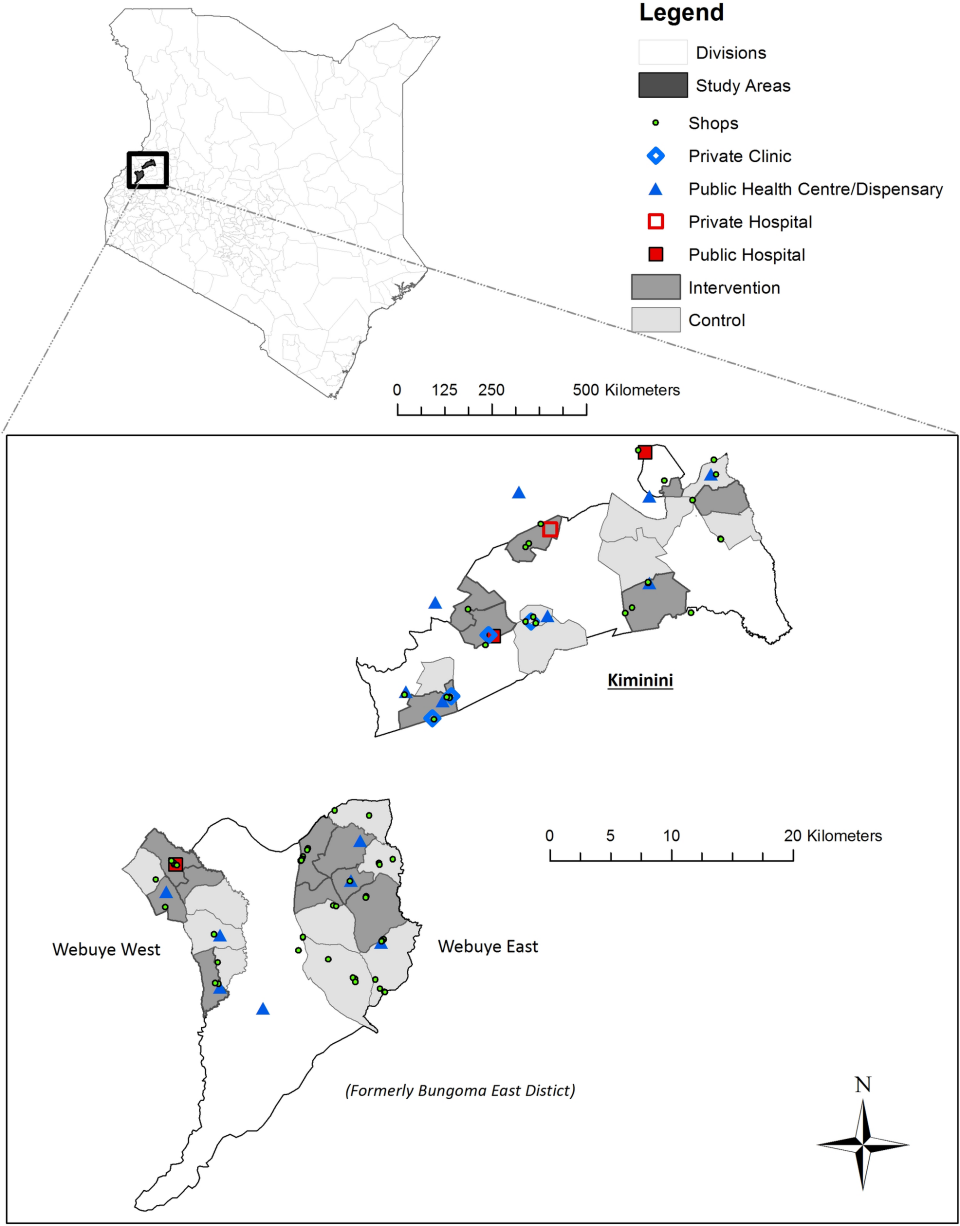
Map of the study area showing intervention and comparison clusters (i.e., CUs). Bungoma East was divided into multiple subcounties after the study began. Webuye West and Webuye East were previously divisions within Bungoma East subcounty and are now separate subcounties. In Laktabai and colleagues [10], we refer to Bungoma East and Kiminini. Here, we have used the updated administrative structure. CU, community unit.

We estimate the public health impact of free malaria testing and conditional antimalarial subsidies implemented through a partnership between CHWs and the private retail sector. We conducted a cluster-randomized trial in 32 community units (CUs; i.e., clusters) across a population of more than 160,000 people (Fig 1). CHWs offered free, community-based malaria testing using RDTs to individuals experiencing a malaria-like illness. The conditional subsidy was in the form of a voucher issued to participants with a positive malaria test, which could be redeemed at a local drug retailer for the purchase of a quality-assured ACT at a reduced, fixed price. The voucher was intended to create an incentive for an individual with suspected malaria to seek out a test from their CHW (individual benefit) while also allowing drug subsidies to be targeted to those with confirmed malaria (community-wide benefit).

The full study protocol has been published [27]. Ethical approval was granted by Moi University Institutional Research and Ethics Committee and Duke University Institutional Review Board. The trial is registered at ClinicalTrials.gov (NCT02461628).

### Study area

We conducted the study in 3 subcounties in western Kenya (Webuye West, Webuye East, and Kiminini), which all experience moderate to high perennial transmission with seasonal peaks. More than 40% of the population of the 3 subcounties live below the poverty line [28].

Each subcounty is divided into CUs. A CU is a geographically defined administrative unit consisting of approximately 1,000 households served by 10 to 22 CHWs who are supervised by a government-employed community health extension worker. Each CHW is assigned to specific households within their CU. All CUs with active, preexisting CHWs in each subcounty were eligible to participate in the study (10 Webuye East, 8 Webuye West, and 14 Kiminini; Fig 1). CHWs were established in the area beginning in 2007 although some areas selected and trained CHWs much later. The package of services provided by CHWs is laid out in Kenya’s Community Strategy plan [29] and includes health education, disease prevention, and referral services. CHWs are unpaid volunteers although they are sometimes engaged in specialized campaigns for which they often receive some transport allowance or incentives.

### Randomization

The unit of randomization was the CU. CUs were divided into 5 strata based on subcounty and whether a public health facility that offered malaria diagnostic testing was located within the CU (Fig 1). Each stratum contained an even number of CUs that were assigned equally to 2 distinct groups by using a random draw for each CU, which was taken from a uniform distribution on the interval [0,1]. The lower half of values of the random draws were assigned to the first group and the upper half to the second group. Subsequently, which of the 2 groups was allocated to intervention was determined using a second (single) random draw from a uniform distribution on the interval [0,1]. It was predetermined that if the random draw was less than or equal to 0.5, the first group would be assigned to intervention, if greater than 0.5 to control. Randomization and allocation to treatment arm was performed by the primary study statistician (ELT) using Stata version 13. Because of the nature of the intervention, study participants could not be blinded to treatment arm.

### Procedures

Prior to the study, CHWs provided standard services as described above. In intervention CUs, we trained CHWs to perform malaria RDTs and to offer free testing for residents of their CU. We sensitized intervention CUs to the program, including the conditional voucher, through meetings with community leaders, appearances at public meetings, and printed posters in retail outlets. Any resident in an intervention cluster who was older than 1 year and reported a fever or malaria-like illness in the previous 48 hours was eligible for a free RDT and could contact a CHW to request a test. The CHW obtained written informed consent, performed the RDT, and explained the results to the participant. A parent or guardian provided written informed consent for a child less than 18 years, and children 8 years and above gave verbal assent for testing. If the test was positive, the CHW provided a serialized voucher to the participant to be used within 2 days of testing. Local medicine retailers serving residents of the intervention clusters—who routinely stocked quality-assured artemether lumefantrine (AL; i.e., the first-line ACT recommended for uncomplicated malaria by the Government of Kenya)—were enrolled in the study, and study participants could redeem their vouchers for a discounted quality-assured AL (“conditional subsidy”). The price to the participant was fixed according to the age-specific dose (Table 1). The CHW also provided a written referral note documenting the test result, which the participant could present at any local health facility. The CHW immediately referred individuals who were seriously ill to a health facility and also advised malaria-negative participants to visit a health facility. Pregnant women were eligible for a test but did not receive a voucher. Instead, they were referred to the nearest health facility. The referral notes and vouchers were filled out in carbon-copy booklets in which one copy was retained and given to the study team; they were scanned, and data were extracted using Captricity (Oakland, CA). We met monthly with the CHWs to replenish supplies and review used RDTs, which the CHWs were instructed to keep. The study team collected vouchers that were redeemed at the retail outlets. The difference between the retail price and the subsidized price for the drugs dispensed was reimbursed to the shop owner.

**Table 1:** Prices for a 3-day course of first-line ACT (AL) in the retail sector and study-subsidized prices for voucher holders. Prices are in KES. 1USD is approximately 100 KES. Prices were set to ensure that the price of the adult dose was equivalent to the target price under the AMFm program. For comparison, the price of a diagnostic test at a facility is 50–100 KES for patients over 5 years and free for children 5 years and below.

Age group	Number of tablets	Unsubsidized price (KES)^1^	Study-subsidized price for voucher holders (KES)
Adult dose	24	100–120	40
9–15 years	18	80	20
3–8 years	12	50	15
1–2 years	6	40	10
<1 year	-	-	Not eligible

^1^Unsubsidized refers to the prevailing retail price available to customers without a voucher, which includes a partial subsidy provided by the government through funding from DFID. These are the prices observed in our study outlets, which are similar to those reported from a nationwide survey [30].

Abbreviations: ACT, artemisinin combination therapy; AL, artemether lumefantrine; AMFm, Affordable Medicines Facility-malaria; DFID, Department For International Development, UK; KES, Kenya shillings.

In control areas, households had access to normal avenues for treatment, including government health facilities, private health facilities, and pharmacies or retail medicine outlets. CHWs continued to provide health promotion and referral services according to government guidelines as they had before the study.

We collected individual-level study outcomes based on a population-based survey sampling strategy in order to assess the community-level impact of the testing and voucher program. We conducted repeated cross-sectional household surveys at 4 time points: baseline, 6 months, 12 months, and 20 months post baseline. The final survey was planned for 18 months post baseline, but a nationwide doctor’s strike forced a 2-month delay. Each cross-sectional survey targeted households in which at least one member >1 year of age had a fever or malaria-like illness in the last 1 month. A systematic random sampling approach was used in which the first household in each cluster was selected by starting at a random household and visiting every *n*th household, such that *n* is the sampling interval that gives the desired number of households in that cluster. Each survey represents an independent random sample, and only 1 random fever per household was included. The sampling interval and starting point were different in each wave of data collection in order to minimize the occurrence of the same household being surveyed in multiple waves. The parent or guardian of a child less than 18 years responded on their behalf. We recorded information about treatment seeking, testing, and drug consumption. Trained field researchers interviewed respondents, and information was collected via electronic forms on android tablets. Verbal informed consent was obtained from respondents who participated in the surveys.

### Outcome measures

The primary outcome that we compare across the intervention and control arms is uptake of testing, defined as the percent of fevers in the previous 4 weeks that receive a malaria diagnostic test from any source. The main secondary outcomes are the percent of all ACTs used that were taken by people with a positive malaria test (rational ACT use; see Box 1) and the percent of all ACTs used that were taken by people without a test. Other predefined secondary outcomes are test adherence (defined as positives taking ACT or negatives not taking ACT amongst those tested) and targeted ACT use (defined as positives taking ACT or negatives not taking ACT amongst all fevers [Box 1]). We also report the percent of those with a positive test who took an ACT after a test, the percent of those with a negative test who took an ACT after a test, and the percent of those with no test who took an ACT. We present descriptive results for the percent of test-positive people who took an ACT and received an appropriate dose.

**Box 1. Definition of outcomes**

**Primary outcome**

**Uptake of diagnostic testing:** The proportion of recent fevers (in the past 4 weeks) that received a malaria diagnostic test of any type (i.e., RDT or microscopy).

**Secondary outcomes**

We chose 3 secondary outcomes that are each a composite of other outcomes. Each has a different denominator and provides a different understanding of ACT use.

**Test adherence:** Positives taking ACT or negatives not taking ACT evaluated among all individuals tested. Individuals not tested are not included. This is a measure of how people use information from a test.

**Targeted ACT use:** Positives taking ACT or negatives not taking ACT evaluated among all fevers. Individuals not tested are included in the denominator. This is a measure of the population-level targeting of ACTs among recent fevers.

**Rational ACT use:** The percent of all ACTs used that were taken by people with a positive malaria test. WHO defines rational drug use as when “patients receive medications appropriate to their clinical needs, in doses that meet their own individual requirements, for an adequate period of time, and at the lowest cost to them and their community.” [http://www.who.int/medicines/areas/rational_use/en/]. We tailor this definition to focus on the proportion of all ACTs consumed in the last month that were used “rationally” according to the results of the diagnostic test.

We also report the following simple outcomes:

**Took an ACT after a positive test:** Positives who took an ACT evaluated among all those who tested positive.

**Took an ACT after a negative test:** Negatives who took an ACT evaluated among all those who tested negative.

**Took an ACT without a test:** Untested individuals who took an ACT evaluated among all untested participants.

**Received an age-appropriate dose of ACT:** Individuals who received the correct number of AL tablets based on their age evaluated among all those who took ACT. Information for this outcome was not available at baseline; therefore, we have restricted our treatment of this outcome to a descriptive analysis.

Less than 2% of participants used an ACT other than AL, and none of those were quality-assured (WHO-preapproved). Therefore, we restrict our analysis to AL use.

### Statistical analysis

Details of our sample size calculation are described elsewhere [27]. Briefly, the target sample size of eligible individuals with a fever in the last 4 weeks was 640 per arm (i.e., 40 per CU) at each time point, which—assuming 22.2% of households would have an eligible member—would require contacting 5,766 households. Power for our stratified design was conservatively based on a two-tailed *t* test for the comparison of 2 proportions at a single time point under a matched design [31]. To attain an overall type I error (α) of 5%, we used a Bonferroni correction-fixed α at 1.67% (5%/3) for each of the 3 follow-up time points [32]. Based on pilot data, we hypothesized an increase from 31% in control to 70% in intervention in our primary outcome of uptake of testing. Assuming a conservative intraclass correlation coefficient (ICC) of 0.073 (corresponding to a coefficient of variation of 0.40), we had more than 95% power to detect this hypothesized effect size.

Given that we designed our study to equally weight each CU in the analysis by sampling the same number of fevers per CU (i.e., 40), we computed survey weights to account for the unequal numbers that were obtained in practice due to the systematic random sampling approach used. These were given by ${weight}_{ik}=\left(\frac{N_{k,total}}{32}\right)/N_{ik},$ such that *I* = 1,…,32 indicates CU; *k* = 0, 1, 2, and 3 indicates baseline, 6 months, 12 months, and 18 months post baseline, respectively; *N*_*k*,*total*_ represents the total number of fevers surveyed across all CUs at time point *k*; and *N*_*ik*_ represents the actual number of fevers in CU *i* at time point *k*. All descriptive statistics and all regression analyses included these weights. For reporting purposes, weighted frequencies were rounded to the nearest whole number. As a consequence, weighted totals may differ to observed totals.

All analyses were based on the intention-to-treat principle. Study statisticians were blinded to treatment allocation until all results were finalized. All analyses were performed in SAS version 9.4. We estimated relative effects (risk ratios [RRs]) and absolute effects (risk differences [RDs]) to compare individual-level binary outcomes between intervention and control arms at each time point. We used the modified Poisson [33] approach and a linear-binomial model (i.e., with identity link) to estimate RR and RD, respectively. To account for correlation within CUs, we used generalized estimating equations (GEEs) with robust standard errors and the Kauermann and Carroll (KC) correction [34] to avoid inflated type I error because of relatively few clusters (*N* = 32). We selected an independence working correlation matrix to obtain unbiased treatment effects due to the numerical implementation of weighted GEEs in the SAS GLIMMIX procedure. We included fixed effects for strata (to account for the stratified design) and adjusted for the baseline level of the outcome variable (as log-cluster–level proportion or cluster-level proportion for the RR and RD models, respectively) in order to improve the precision of estimation of the intervention effect [35]. Adjusted models included 4 additional covariates: age (1–4, 5–17, and 18+ years), sex, education level of the respondent, and household wealth index quintile (based on a household asset index following standard methods, see S1 Text) [36]. All secondary and other outcomes were analyzed using the same modelling procedures and adjustment approach. Because 2 adjusted RD models of other outcomes (percentage of participants with a positive test taking ACT and percentage of participants with a negative test taking ACT) did not converge, we used a linear-normal (i.e., linear-probability) model in these 2 cases, and results are presented separately [37]. Given that testing and treatment decisions may depend on the age of the febrile individual, particularly for children below the age of consent (<18) or for individuals in higher-risk age groups (age <5), we evaluated the age subgroup using an interaction between age and treatment arm.

**Fig 2:**
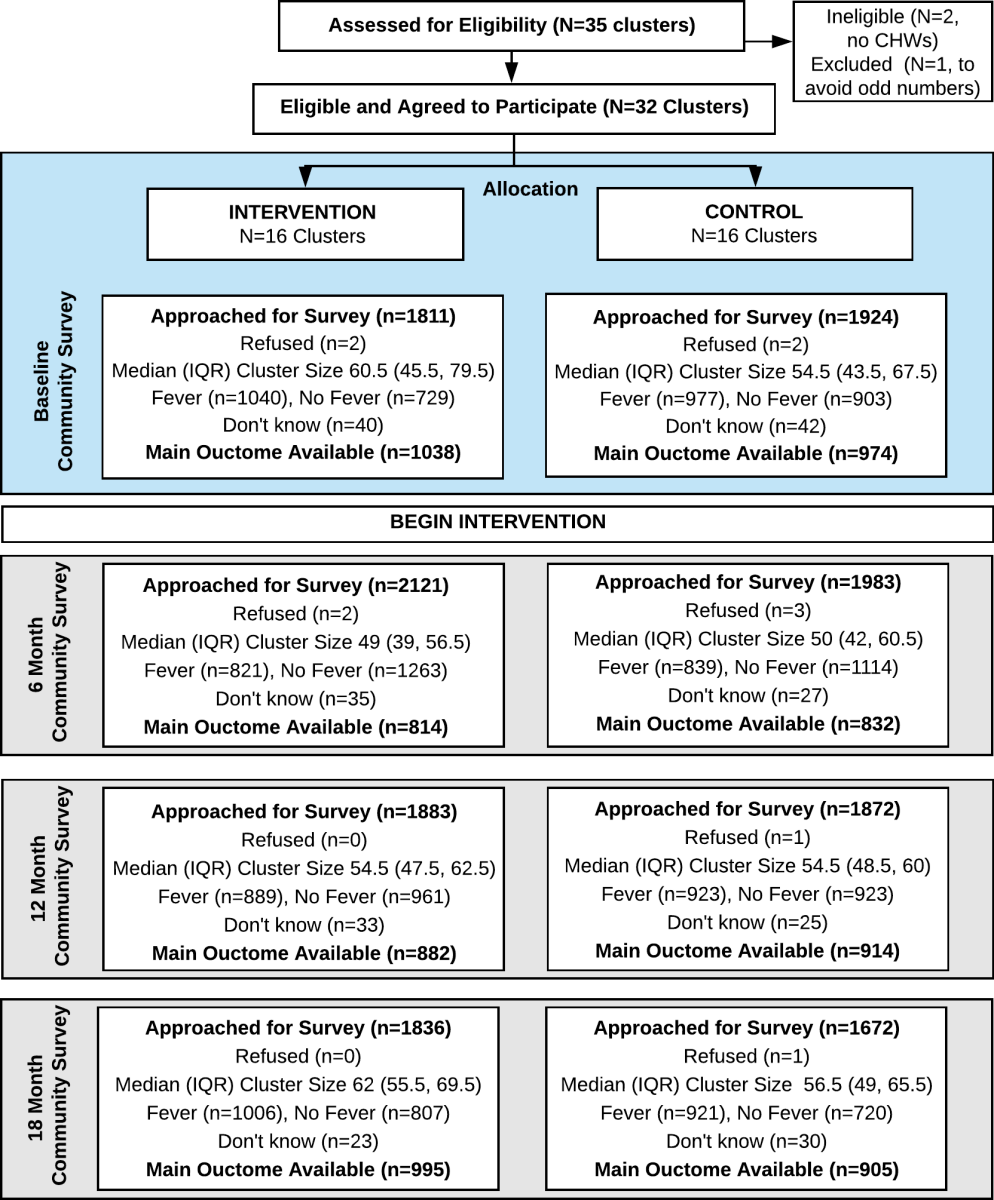
CONSORT diagram. Thirty-two clusters were randomized to 2 arms. All clusters were analyzed at each time point. CHW, community health worker; CONSORT, **CON**solidated **S**tandards **O**f **R**eporting **T**rials; IQR, interquartile range.

Owing to the repeated cross-sectional c-RCT design, we did not need to account for missing data due to attrition of study participants in order to estimate the intention-to-treat effect for the intervention. Instead, survey nonresponse may lead to nonrepresentativeness of the cross-sectional samples. The nonresponse rates did not appear to differ between arms and were overall very low (<3 per arm per survey), therefore we did not perform additional analyses to account for nonresponse (Fig 2). In line with our power calculation based on the primary outcome of uptake of testing evaluated at 3 time points, we adjusted for 3 multiple comparisons of the primary outcome. To do so, we used the Benjamini-Hochberg (BH) procedure [38], which controls the false discovery rate and is more powerful than the conservative Bonferroni approach used to calculate sample size.

All data are available from the Dryad Digital Repository (https://doi.org/10.5061/dryad.59p4111) [39].

## Results

The study area included over 160,000 people (based on estimates from the 2009 census) in 32 clusters across 3 subcounties (Fig 1). A total of 292 CHWs in 16 intervention clusters were trained to perform RDTs, and 42 retail outlets serving these clusters were enrolled to redeem vouchers.

**Fig 3:**
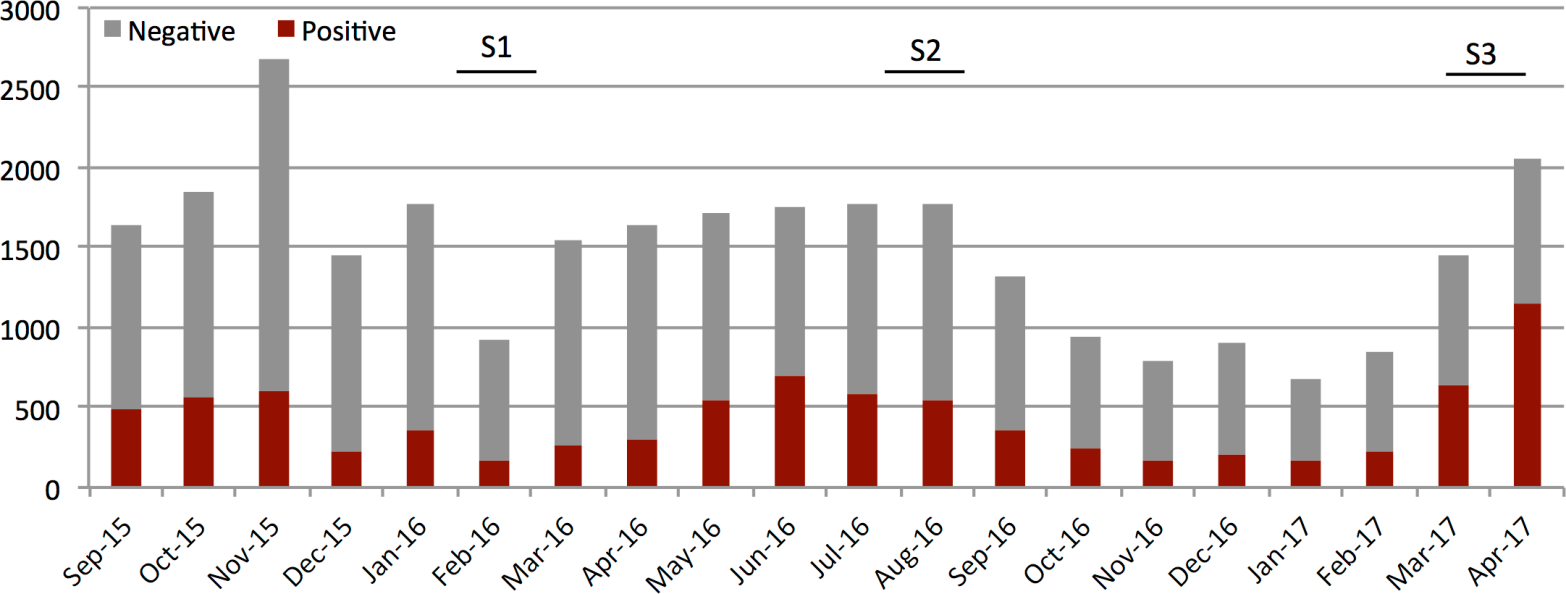
Number of positive (red portion) and negative (grey portion) RDTs performed by CHWs per month over the study period in 16 intervention CUs. The first full month of testing for the first 11 CUs was September 2015, and the first full month of testing for the remaining 5 CUs was October 2015. The study continued until the last CU had participated for 19 months (April 2017). Survey periods of 6, 12, and 18 months are indicated. Monthly fluctuations in testing rates are related to fever prevalence and possibly holiday travel (December–January). Seasonal rains in March–June usher in the high malaria season, which typically continues through July. Annual variations in rainfall and overall transmission are common. CHW, community health worker; CU, community unit; RDT, rapid diagnostic test.

The intervention was launched on July 21, August 17, and September 22, 2015 in Bokoli, Kiminini, and Ndivisi subcounties, respectively. The intervention continued until May 5, 2017. Fig 3 shows the number of tests done per month and the proportion of positive tests over the study period. We had no instances of stock out of RDTs among our CHWs. In total, 32,404 RDTs were conducted by the CHWs, and 33.7% (*n =* 10,870) of those were positive. All RDT interpretations were counterchecked by the study team. Vouchers for qualified ACTs were redeemed by 93.9% (*n =* 10,204) of voucher recipients.

### Impact on malaria diagnostic testing and ACT consumption

A total of 7,416 households with fever in the last 1 month participated in the surveys: 2,017, 1,660, 1,812, and 1,927 at baseline, 6 months, 12 months, and 20 months, respectively. Table 2 describes the weighted characteristics of the observed 2,017 survey participants at baseline in the intervention and control arms. Fig 2 shows the participant enrollment flowchart. All means and frequencies were weighted such that each CU was represented equally in the summary statistics. The median distance from a household to a public health facility at baseline was 2.3 km, and the median distance to a medicine retailer was half as far. At baseline, the majority of reported fevers were in children (62.2%; *n =* 1,253), and 57.3% (*n =* 1,155) of participants reported visiting a medicine retailer for their febrile illness. Overall, 40.3% (*n =* 810) of all fevers used an ACT purchased at a medicine retailer. Baseline characteristics of intervention and control arms were comparable (Table 2).

**Table 2:** Weighted characteristics of participants and respondents (for participants <18 years) in the baseline survey by arm*. Weighted characteristics for each follow-up survey can be found in S1 Table.

Variable: Number (%), unless otherwise stated	Control (*N =* 1,009)	Intervention (*N =* 1,009)
***Age of participant***^***1***^		
Under 5	221 (21.9%)	239 (23.7%)
5 to 17	388 (38.5%)	405 (40.2%)
18+	399 (39.6%)	364 (36.1%)
***Gender of participant***^***2***^		
Male	379 (37.6%)	416 (41.3%)
Female	629 (62.4%)	592 (58.7%)
***Wealth index (quintile)***		
0 to 20th	216 (21.7%)	211 (21.1%)
>20.0 to 40th	169 (17.0%)	182 (18.2%)
>40.0 to 60th	202 (20.3%)	218 (21.8%)
>60.0 to 80th	196 (19.7%)	207 (20.8%)
>80.0	212 (21.3%)	181 (18.1%)
Missing	13 (.%)	10 (.%)
***Highest school level of participant (or respondent if participant <18 years)***		
None or less than primary	399 (39.9%)	421 (42.0%)
Completed primary	373 (37.2%)	360 (35.9%)
Completed secondary	230 (22.9%)	221 (22.1%)
Missing	7 (.%)	6 (.%)
***Distance***^***3***^**: *Median (25th*, *75th percentile)***		
Nearest retail medicine outlet (km)	1.3 (0.7–2.3)	1.1 (0.7–1.6)
Nearest private hospital (km)	14.1 (8.5–42.9)	15.2 (9.8–41.5)
Nearest public health center/dispensary (km)	2.2 (1.3–3.2)	2.4 (1.5–3.5)
Nearest public hospital (km)	5.9 (4.1–8.2)	4.1 (2.4–8.3)
***Health-seeking behavior of participant***^***4***^		
Sought treatment in public facility	371 (37.6%)	372 (37.7%)
Sought treatment at a private facility	165 (16.7%)	152 (15.4%)
Sought treatment at a medicine retailer	565 (57.2%)	590 (59.8%)
Had a malaria test	440 (43.6%)	422 (41.8%)
Took an ACT	679 (67.3%)	683 (67.9%)

*Note that the weighted totals are provided by arm, which sum to *n =* 2,018 across arms. Due to rounding, this is slightly different than the observed total of *n =* 2,017 (*n =* 977 control; *n =* 1,040 intervention).

^1^One participant in the control arm and 1 participant in the intervention arm were missing age information.

^2^One participant in the control arm and 1 participant in the intervention arm were missing gender information.

^3^Distance calculated as straight line between 2 points; *n =* 570 participants in the control arm and *n =* 299 participants in the intervention arm were missing valid coordinates.

^4^*n =* 19 participants in the control arm and *n =* 23 participants in the intervention arm were missing data on health-seeking behavior.

Abbreviation: ACT, artemisinin combination therapy.

At baseline, 42.7% (*n =* 862) of participants had a self-reported malaria test for their illness, and 99% of all tests were done in a health facility (*n =* 853). At the end of the study, uptake of malaria testing for febrile illness significantly improved in the intervention arm; 55% (*n =* 524) of recent illnesses were tested for malaria compared to 44.7% (*n =* 423) in the control arm (Table 3). The intervention led to a relative increase in the primary outcome of testing uptake of 20% after 1 year (adjusted RR: 1.20; 95% CI 1.05–1.38, Table 3) and of 25% by 18 months (adjusted RR: 1.25; 95% CI 1.09–1.44), with BH-adjusted *p*-values of 0.015 and 0.005, respectively. Corresponding absolute effects were increases of 9 percentage points [pp] (95% CI 2–15) and 11 pp (95% CI 5–18). At 18 months, 44.2% (*n =* 230) of all tests in the intervention arm were done by a CHW.

**Table 3:** Weighted design-adjusted^1^ as well as design- and covariate-adjusted,^2^ model-estimated, between-arm differences comparing intervention versus control arm in malaria testing behavior, targeted ACT use, rational ACT use (i.e., percent of ACT users testing positive), and percent of ACT users without a test. Sample proportions at each survey time point, including baseline, are also reported for each outcome. Coefficients of variation for each outcome are available in S2 Table. Full regression output can be found in S3 Table.

			**RR**		**RD**	
	Sample Proportions		Design Adjusted	Fully Adjusted	Design Adjusted	Fully Adjusted
Outcome	Control	Intervention	Estimate (95% CI)	Estimate (95% CI)	Estimate (95% CI)	Estimate (95% CI)
**Took malaria test (among all fevers)**^**3**^						
Baseline (*N =* 2,012)	43.7	41.9				
6 months (*N =* 1,646)	46.9	48.4	1.05 (0.91 to 1.22)	1.07 (0.93 to 1.23)	0.03 (−0.04 to 0.09)	0.03 (−0.04 to 0.10)
12 months (*N =* 1,796)	43.4	50.5	1.19 (1.04 to 1.37)	1.20 (1.05 to 1.38)	0.08 (0.02 to 0.15)	0.09 (0.02 to 0.15)
18 months (*N =* 1,900)	44.7	55.0	1.26 (1.09 to 1.45)	1.25 (1.09 to 1.44)	0.11 (0.04 to 0.18)	0.11 (0.05 to 0.18)
**Targeted ACT use (among all fevers)**						
Baseline (*N =* 2,000)	32.3	29.5				
6 months (*N =* 1,646)	39.3	38.8	1.02 (0.86 to 1.21)	1.03 (0.88 to 1.22)	0.01 (−0.06 to 0.07)	0.01 (−0.05 to 0.07)
12 months (*N =* 1,775)	32.4	39.0	1.25 (1.03 to 1.51)	1.26 (1.04 to 1.54)	0.08 (0.01 to 0.14)	0.09 (0.02 to 0.16)
18 months (*N =* 1,873)	32.5	46.0	1.46 (1.20 to 1.79)	1.47 (1.20 to 1.80)	0.15 (0.07 to 0.22)	0.15 (0.08 to 0.22)
**Rational ACT use (among ACT users)**						
Baseline (*N =* 1,381)	46.2	41.7				
6 months (*N =* 1,099)	51.4	44.9	0.91 (0.74 to 1.11)	0.91 (0.75 to 1.11)	−0.04 (−0.14 to 0.05)	−0.04 (−0.14 to 0.05)
12 months (*N =* 1,256)	43.2	48.3	1.15 (0.94 to 1.41)	1.16 (0.94 to 1.43)	0.07 (−0.02 to 0.16)	0.07 (−0.02 to 0.16)
18 months (*N =* 1,402)	44.1	59.6	1.40 (1.19 to 1.64)	1.40 (1.19 to 1.64)	0.17 (0.09 to 0.25)	0.17 (0.10 to 0.24)
**Had no test (among ACT users)**						
Baseline (*N =* 1,381)	49.8	51.8				
6 months (*N =* 1,099)	43.7	46.2	1.03 (0.83 to 1.28)	1.03 (0.83 to 1.27)	0.02 (−0.08 to 0.11)	0.02 (−0.08 to 0.11)
12 months (*N =* 1,256)	53.0	44.8	0.83 (0.71 to 0.98)	0.83 (0.71 to 0.98)	−0.09 (−0.17 to −0.01)	−0.09 (−0.17 to −0.01)
18 months (*N =* 1,402)	51.9	37.3	0.70 (0.59 to 0.83)	0.70 (0.60 to 0.83)	−0.15 (−0.23 to −0.08)	−0.15 (−0.22 to −0.08)

^1^Design-adjusted model: adjusts for baseline CU-level outcome proportion (as log-cluster–level proportion or cluster-level proportion for the RR and RD models, respectively), time indicators for 12 and 18 months, treatment indicator, time *x* treatment interaction, and fixed effects for strata.

^2^Design- and covariate-adjusted model: adds indicators for wealth quintile, participant age (<5, 5–17, 18+), participant female gender, and highest level of education of the respondent (none or less than primary, completed primary, completed secondary).

^3^ We used the BH procedure for determining significance of the 3 tests of the difference between arms at each follow-up time point for our primary outcome of uptake of malaria testing. *p*-Values based on adjusted models are 0.341, 0.015, and 0.005 for the relative risk model at 6, 12, and 18 months, respectively, and 0.346, 0.015, and 0.003 for the RD model at 6, 12, and 18 months, respectively.

Abbreviations: ACT, artemisinin combination therapy; BH, Benjamini-Hochberg; CU, community unit; RD, risk difference; RR, risk ratio.

**Fig 4:**
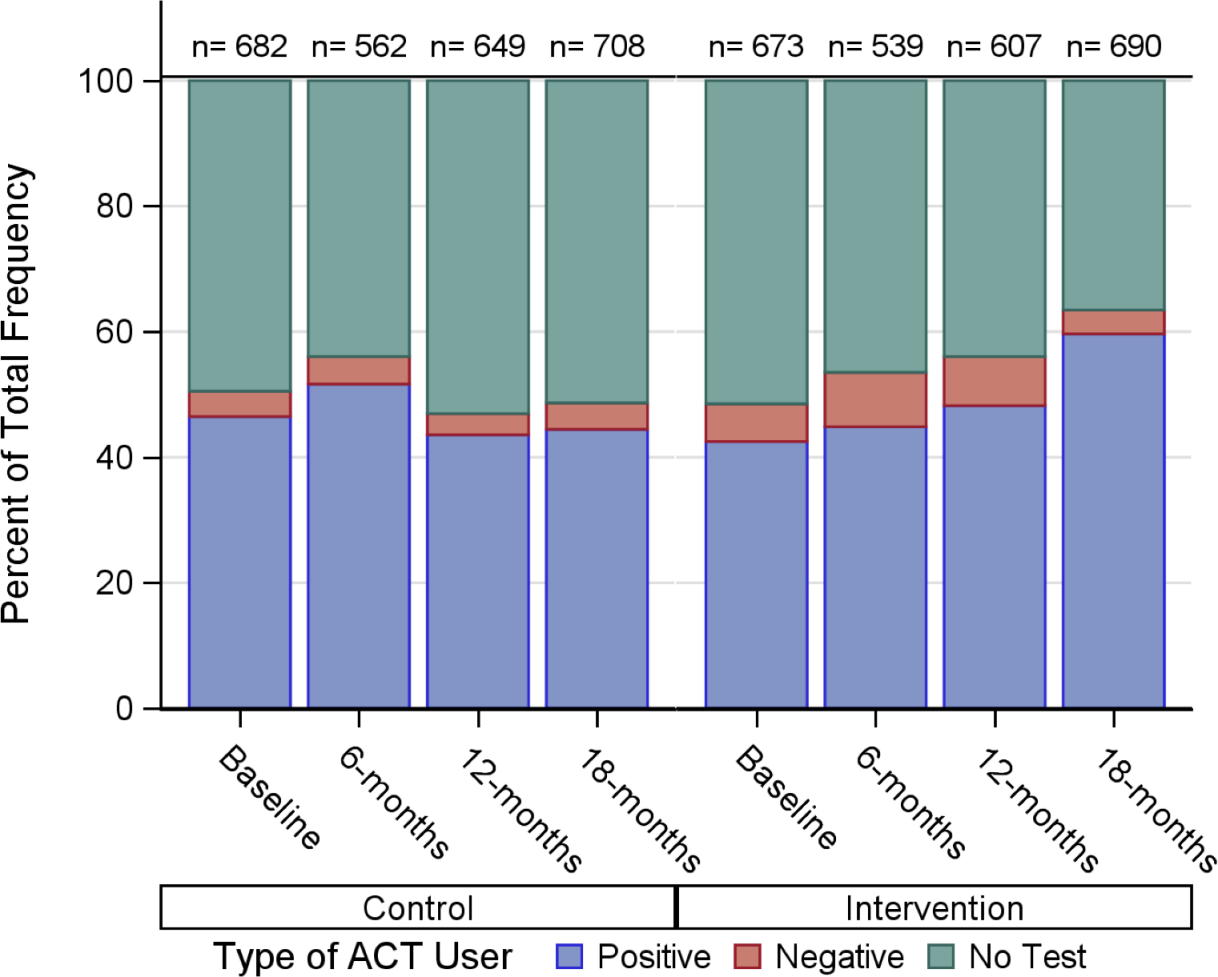
Proportion of ACT consumed by those with a febrile illness in the last month according to their testing uptake and result (positive or negative). Results are reported by intervention arm and survey period. The total number of ACT courses taken is indicated for each bar. ACT, artemisinin combination therapy.

**Fig 5:**
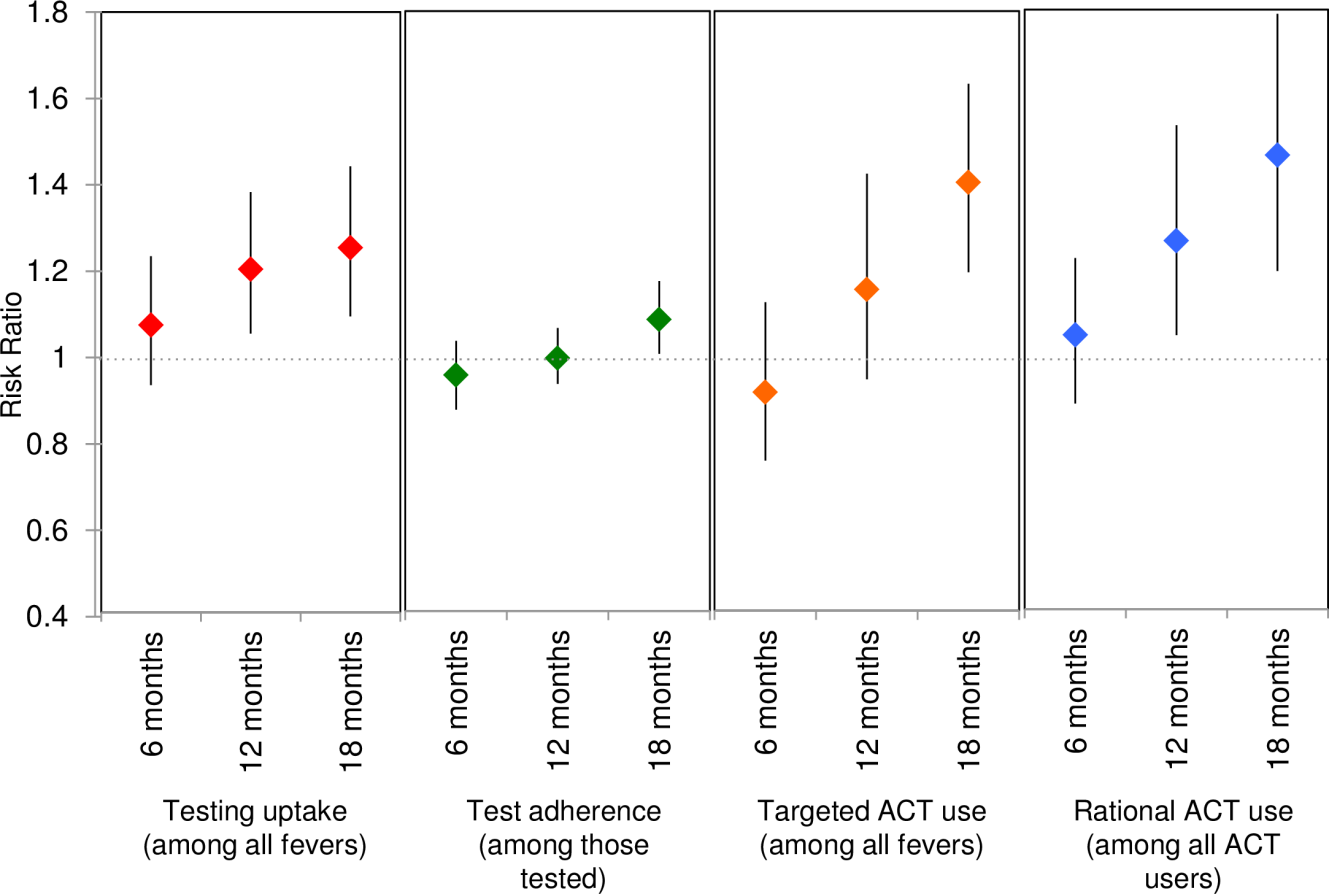
Adjusted modeled RRs and 95% CIs for the primary outcome of uptake of testing and 3 composite outcomes. Test adherence is defined as those who take ACT with a positive test or do not take ACT with a negative test among those tested. Targeted ACT use is defined as the proportion of all fevers that have a positive test and take ACT or a negative test and do not take ACT. Rational ACT use is defined as the proportion of all ACT courses consumed by individuals with a positive test. ACT, artemisinin combination therapy; RR, risk ratio.

ACT consumption was high among survey participants. In the baseline survey, 67.5% (*n =* 1,362) of all participants reported taking an ACT for their illness in the last month. Fig 4 shows the distribution of ACT consumers by testing uptake and test result in each survey. A substantial improvement in rational ACT use was observed by the end of the study period. Overall, the intervention led to a relative reduction of 30% in the proportion of ACT dispensed to those without a test (37.3% [*n =* 252] versus 51.9% [*n =* 364]; ARR 0.70; 95% CI 0.60–0.83, Table 3) and to a relative increase of 40% in the proportion of ACT doses that were dispensed to individuals with a positive malaria diagnostic test (44.1% [*n =* 309] versus 59.6% [*n* = 403]; ARR 1.40; 95% CI 1.19–1.64, Table 3, Fig 5). In addition, the proportion of ACT users in the intervention arm who received a correct, age-appropriate dose at 18 months was 69.1% (*n =* 487) compared to 60.9% (*n =* 405) in the control arm (S4 Table).

Parsing ACT consumption by test result shows that, at baseline, 83.6% (*n =* 539) of individuals with a positive test reported taking an ACT, and 34.3% (*n =* 42) of individuals who tested negative also took an ACT. After the intervention, adherence to malaria test results was higher among both positive and negative cases in the intervention arm at both the 12- and 18-month follow-up survey time point, resulting in a significantly higher proportion of test adherence among those tested at 18 months (88.5% [*n =* 428] versus 80.7% [*n =* 305]; ARR = 1.08; 95% CI 1.00–1.17, Fig 4, Table 4). In addition, ACT consumption by untested fevers in the intervention arm was 13% lower relative to the control arm (60.2% [*n =* 267] versus 71.6% [*n =* 400]; ARR 0.87; 95% CI 0.79–0.96, Table 4). Together, higher testing uptake, improved adherence to the test, and lower ACT consumption among those without a test led to substantial improvements in population-wide ACT targeting in the intervention arm at 18 months (46.0% [*n =* 428] versus 32.5% [*n =* 305]; ARR = 1.47; 95% CI 1.20–1.80, Table 3, Fig 4).

**Table 4:** Weighted design-adjusted^1^ as well as design- and covariate-adjusted,^2^ model-estimated, between-arm differences in ACT use among those who test positive, negative, without a test, and the overall percent who adhere to the test results between intervention and control arms. Sample proportions at each time point, including baseline, are also reported for each outcome. RD models did not converge for all outcomes. RDs were estimated using alternative methods and are presented in S5 Table.

			RR	
	Sample Proportions		Design Adjusted	Fully Adjusted
Outcome	Control^3^	Intervention^4^	Estimate (95% CI)	Estimate (95% CI)
**Took ACT after a positive test**				
Baseline (*N =* 634)	83.7	83.5		
6 months (*N =* 602)	89.7	88.5	0.98 (0.92–1.05)	0.98 (0.92–1.04)
12 months (*N =* 628)	84.4	87.9	1.03 (0.97–1.10)	1.02 (0.96–1.09)
18 months (*N =* 782)	83.8	90.0	1.07 (0.98–1.16)	1.06 (0.98–1.15)
**Took ACT after a negative test**				
Baseline (*N =* 122)	31.7	36.8		
6 months (*N =* 179)	42.4	37.7	0.86 (0.51–1.44)	0.82 (0.50–1.34)
12 months (*N =* 128)	37.8	31.9	0.81 (0.38–1.73)	0.83 (0.39–1.78)
18 months (*N =* 90)	45.6	29.9	0.64 (0.35–1.19)	0.69 (0.36–1.32)
**Took ACT with no test**				
Baseline (*N =* 1,240)	62.4	61.9		
6 months (*N =* 860)	56.5	58.6	1.06 (0.91–1.24)	1.09 (0.94–1.26)
12 months (*N =* 1,001)	69.2	61.3	0.91 (0.81–1.02)	0.92 (0.83–1.02)
18 months (*N =* 998)	71.6	60.2	0.87 (0.79–0.95)	0.87 (0.79–0.96)
**Test adherence among all tested**				
Baseline (*N =* 756)	81.5	80.0		
6 months (*N =* 781)	84.8	80.3	0.95 (0.87–1.03)	0.94 (0.87–1.02)
12 months (*N =* 755)	82.6	83.2	1.01 (0.94–1.08)	1.00 (0.93–1.07)
18 months (*N =* 868)	80.7	88.5	1.09 (1.01–1.18)	1.08 (1.00–1.17)

^1^Design-adjusted model: adjusts for baseline CU-level outcome proportion (as log-cluster–level proportion or cluster-level proportion for the RR and RD models, respectively), time indicators for 12 and 18 months, treatment indicator, time *x* treatment interaction, and fixed effects for strata.

^2^Design- and covariate-adjusted model: adds indicators for wealth quintile, patient age (<5, 5–17, 18+), female gender, and highest level of education of the respondent (none or less than primary, completed primary, completed secondary).

^3^*N =* 3 control participants at baseline, *N =* 1 at 12 months, and *N =* 3 at 18 months tested positive for malaria but had missing information on whether ACT was taken before or after malaria test. Data on chronology of ACT use were missing for 6-month wave.

^4^*N =* 6 intervention participants at baseline, *N =* 3 at 12 months, and *N =* 3 at 18 months tested positive for malaria but had missing information on whether ACT was taken before or after malaria test. *N =* 1 control participant and *N =* 1 intervention participant at 12 months tested negative for malaria but had missing information on whether ACT was taken before or after malaria test. Data on chronology of ACT use were missing for 6-month wave.

Abbreviations: ACT, artemisinin combination therapy; CU, community unit; RD, risk difference; RR, risk ratio.

We did not observe increased use of antibiotics following the intervention. At baseline, 32.5% (*n =* 655) of all recent fevers used an antibiotic (63.7% [*n =* 94] of malaria-negative fevers, 39.3% [*n =* 279] of malaria-positive fevers, and 24.0% [*n =* 276] of untested fevers; S6 Table). By 18 months, in the intervention arm, 22.6% (*n =* 217) of recent fevers reported taking an antibiotic (47.3% [*n =* 31] of negative; 25.2% [*n =* 114] of positive; and 15.5% [*n =* 67] of untested; S6 Table).

### Sensitivity analyses

We looked at the influence of participant age on the intervention effect for children 5 years and below, all children less than 18 years, and adults (18 years and above). There were no significant differences in treatment effects when children less than 5 years were compared to older participants. There were significant age effects when children less than 18 years were compared to adults (S7 Table). Age-stratified models showed a larger effect of the intervention in children (less than 18 years) than models that included all participants for the primary outcome of testing and the 3 main secondary outcomes related to ACT use. For example, by 18-month follow-up, the uptake of testing among children was 37% higher in the intervention arm than the control arm (56.8% [*n =* 351] versus 42.0% [*n =* 252]; ARR = 1.37; 95% CI 1.19–1.58), and targeted ACT use across all fevers in children was 60% higher in the intervention arm (49.1% [*n =* 299] versus 31% [*n =* 184]; ARR = 1.60; 95% CI 1.31–1.97).

We also investigated whether estimates of intervention effects differed when we restricted our analyses to those who provided documentation of their malaria test result (S8 Table). We saw a slight increase in the effect of the intervention on testing, targeted ACT use, and rational ACT use at the 18-month survey. However, this group may be slightly biased toward intervention users because the CHWs always provided documentation of testing.

## Discussion

In 2016, there were an estimated 216 million cases of malaria globally, but more than 400 million courses of ACT distributed [40]. Overconsumption of ACTs on this scale has grave implications for the spread of antimalarial resistance [18, 41] and results in poor outcomes for inappropriately treated nonmalaria fevers [11]. In our study area, nearly 70% of individuals with a recent fever reported taking an ACT, two-thirds of which was sourced from the retail sector. Against this background of very high ACT consumption, we rigorously tested a sustainable and scalable community-based intervention designed to increase malaria diagnostic testing and improve ACT targeting. Our intervention was specifically designed to reach individuals purchasing drugs over the counter and to incorporate the retail sector, which delivers the majority of ACTs in Kenya [30]. A particular strength of our study is the evaluation of outcomes in a representative sample of malaria-like illnesses in the population, rather than only in those who access the intervention. As a result, we were able to estimate the impact of the intervention on malaria diagnostic testing rates and targeting of ACTs among all fevers in the population as well as on the proportion of the total ACT consumed by those with a positive test. We find that population-wide targeting and rational use of ACTs improved by 46% and 40%, respectively, relative to the control arm.

We designed our intervention to leverage the simple demand response—in which consumers purchase less of a good when the price is higher—rather than complex regulatory strategies to target delivery of drugs. During the study period, retail-sector ACTs in Kenya were partially subsidized by the government. In the absence of these subsidies, the price difference for those with a positive test would be even larger and potentially present a greater incentive for testing, thereby enhancing uptake of testing and rational ACT use. In our study, improved targeting of ACTs was due to a combination of modest improvements in appropriate ACT consumption by those with a malaria test and a reduction in ACT consumption by those without a test. The latter may be at least partly explained by self-selection into the tested group by participants who strongly believe they have malaria or may be more likely to use information from a diagnostic test.

It is worth noting that the impact of the intervention on testing grew incrementally over time. The effect, which was small at 6 months, was large by 12 months and showed an additional improvement by 18 months. Impact on ACT consumption took even longer to emerge and was not apparent until 18 months. It is likely that targeting may have continued to improve beyond 18 months. This points to the importance of allowing sufficient time for penetration of the intervention, adoption, and positive learning experiences before evaluating the population-wide impact of community-based interventions. This is especially pertinent for interventions that seek to influence individual behavior at the community level, where individuals’ prior beliefs about the effectiveness of a new technology might take time to evolve.

Our intervention approach—community-based testing coupled with a diagnosis-dependent voucher to be redeemed at a retail outlet—is a hybrid approach between that of offering RDTs in retail outlets and community case management (CCM) of malaria, in which CHWs perform RDTs and dispense free ACTs to RDT-positive individuals. In our hybrid model, we rely on the robust supply chain of the retail sector to maintain a supply of ACTs. CHWs are still responsible for the RDTs, but in case RDTs are out of stock, this does not disrupt access to ACTs. There were 3 specific goals of our intervention design: (1) create a mechanism and an incentive (in the form of the voucher) for individuals who routinely seek care in the retail sector to receive a diagnostic test, (2) target the subsidy to individuals with parasitologically confirmed malaria by means of the voucher, and (3) place the information from the test in the hands of the individual, allowing them to decide how to act on it. This third aspect is distinct from most other approaches in which the patient plays a passive role, and it is the health care worker, CHW, or shop attendant who acts on the results. Shop-based [42] or community-based [43–47] testing and treatment studies have focused on ACT use among participants contacting the intervention. Therefore, it is not possible to compare our results at the population level to these studies. A single study [48] reported community-level testing rates in areas with and without retail outlets conducting RDTs, and they showed a very small increase in testing uptake (5.6 pp). Furthermore, all of these studies report on whether the CHW or shop attendant dispensed ACT following a test but not what the patients did afterwards, e.g., seek an ACT elsewhere, leading to an incomplete picture of ACT targeting. In our intervention, when we consider only individuals who were tested by a CHW, ACT consumption following a positive test was nearly as high as is seen in CCM studies (92.3% at 18 months compared to 99%) [45, 46].

Prior to retail-sector subsidies or widespread availability of RDTs, Cohen and colleagues [7] offered a refund for the price of an RDT to individuals testing positive at a retail outlet and purchasing an ACT. They showed no effect of the refund offer on testing rates or treatment with ACTs. In an individually randomized pilot study [49], we offered a conditional ACT subsidy ($0.55) to participants with a positive RDT and observed rates of test adherence and targeted ACT use comparable to those reported here. In our previous work, we observed suboptimal ACT consumption by those with a positive test when the ACT was unsubsidized. In the current study, we note that ACT use by those testing positive was highest among individuals tested by the CHW (92.3%) who had access to a voucher. This provides evidence that the subsidy level offered by the voucher and the patient contribution is not prohibitive. In addition, a higher proportion of ACT users in the intervention arm received an age-appropriate dose, indicating that the voucher allowed them to purchase the correct dose.

This work has several limitations. First, we are not able to separate the effects of free testing from the voucher on uptake of testing. A similar study that deploys free testing in the absence of a conditional voucher in the control arm would be required to resolve this. Second, we were not able to continue the intervention in order to measure the long-term effects and therefore do not know the maximum possible impact that might have been achieved. Third, our outcomes are based on self-reporting by the participant or respondent. However, we stratified our analysis to include only those individuals with documentation of a test (compared to those without a test, S8 Table), and the intervention effect on all outcomes did not differ substantially from those presented here. This increases our confidence in the self-reported outcomes. Fourth, as in other community-based malaria test-and-treat studies (i.e., [47, 50–52]), we did not provide further testing and treatment to malaria-negative patients; instead, we referred them to a health facility and therefore cannot be certain that they received correct treatment. Finally, though our survey design used repeated cross-sectional sampling, it is possible that the same household and/or febrile individual was surveyed at multiple survey time points and any resulting correlation was not explicitly modeled in our regression analysis. Fortunately, such repeated sampling was expected to be rare because both the sampling starting point and the sampling interval were different for each wave. Moreover, we used sandwich variance estimation, which is robust to misspecification of the variance structure in GEE analyses [34].

These limitations are balanced by the unique strengths of the study. As mentioned above, a particular strength of the study is the estimation of population-wide impact of the intervention on testing and ACT use, rather than outcomes measured only in those accessing the intervention. The cluster-randomized controlled design implemented in a large population enhances generalizability of our findings. We saw almost no contamination between the arms; only 8 participants reported being tested for malaria by a CHW in the control arm.

We offer an innovative approach that can enhance the sustainability of publicly funded, private-sector subsidies and improve malaria case management in the private sector by (1) targeting ACTs to individuals who objectively need them, (2) leveraging the effective supply chain of the private retail sector, and (3) allowing consumers without a confirmed infection to contribute more to the cost of the drug. Our results have significant policy implications. We demonstrate a high demand for malaria diagnostic testing and show that individuals use the information from a test. Moreover, these effects appear to increase over time. Scale-up of such a program would require thoughtful attention to monitoring (i.e., detecting deviations from expected rates of testing or test positivity and random checks); however, a similar voucher program for insecticide-treated nets in Tanzania showed little evidence of abuse [53]. In our study, shop attendants collected both the voucher and the positive RDT from the participant, which were then exchanged for payment. This ensured that the number of vouchers roughly equaled the number of positive tests. Larger-scale implementation of targeted subsidies could be achieved in a number of ways without necessarily relying only on CHWs to provide testing and vouchers.

In summary, we demonstrate that it is possible to target ACT subsidies to diagnostically confirmed malaria cases. Allocation of subsidy dollars between testing and treatment for test-positive individuals may present a better use of programmatic resources than unconditional private sector subsidies.

## Supporting information

S1 Text Calculating the wealth index. (DOCX)

S2 Text Statistical analysis plan. (DOCX)

S1 Protocol Full study protocol. (PDF)

S1 CONSORT checklist (DOCX)

S1 Table Weighted characteristics of febrile participants and respondents (for participants <18 years) by arm and follow-up survey time point. (DOCX)

S2 Table Coefficient of variation for study outcomes measured at baseline. (DOCX)

S3 Table Complete regression output for weighted design-adjusted and weighted design- and covariate-adjusted, model-estimated primary study outcome including all covariates. (DOCX)

S4 Table Number (percentage) of AL users who received a correct dose, an underdose, or an overdose according to their age. AL, artemether lumefantrine.
(DOCX)

S5 Table Study outcomes in Table 4 estimated using both the linear-binomial and linear-normal approaches to estimating RDs. RD, risk difference.
(DOCX)

S6 Table Weighted summaries of antibiotic use of febrile participants and respondents (for participants <18 years) by arm, follow-up survey time point, and malaria testing behavior. (DOCX)

S7 Table Age-stratified, weighted design-adjusted, and weighted design- and covariate-adjusted models for main study outcomes. (DOCX)

S8 Table Weighted design-adjusted and weighted design- and covariate-adjusted models for main study outcomes restricted to nontesters and those who had a record observed for their test. (DOCX)

## Abbreviations

ACT: artemisinin combination therapy
AL: artemether lumefantrine
AMFm: Affordable Medicines Facility-malaria
BH: Benjamini-Hochberg
CCM: community case management
CHW: community health worker
CONSORT: **CON**solidated **S**tandards **O**f **R**eporting **T**rials
CU: community unit
DFID: Department for International Development
GEE: generalized estimating equation
ICC: intraclass correlation coefficient
IQR: interquartile range
KC: Kauermann and Carroll
KES: Kenya shillings
pp: percentage points
RD: risk difference
RDT: rapid diagnostic testing
RR: risk ratio
