# Supplementary material for: Improving rational use of ACTs through diagnosis-dependent subsidies: Evidence from a cluster-randomized controlled trial in western Kenya
Source: PLoS Med. 2018 Jul 17;15(7):e1002607. doi: 10.1371/journal.pmed.1002607 (PMC6049880; doi:10.1371/journal.pmed.1002607)
Supplement: S1 Text — (DOCX) [file pmed.1002607.s001.docx]

**S1 Text:** Calculating the Wealth Index

Variables used and methods included to create the wealth index were based on methods developed by Demographic and Health Surveys (DHS)^1^. Frequency tables were created for each of the indicators in the wealth index. Sparsity in categories was improved by collapsing categories into meaningful dimensions (e.g. wall materials was grouped into “porous” and “non-porous”; roof type into thatched vs. not). Once meaningful categories were created for each of the categorical variables, binary indicators were created to represent each “category” (Supplementary Table 1). For all index variables, the binary value of one corresponded to the item associate with higher SES.

Because our variables were categorical, rather than the normally distributed continuous variables appropriate for the default Pearson correlation matrix constructed as part of traditional principal components analysis (PCA) analysis, a polychoric correlation matrix was computed for the final set of variables and used in the subsequent factor analysis. retaining only the first factor.^2^ To complete the wealth index, a factor score was computed for each household. Construction of the wealth index was performed using the polychoricpca package^3^ in Stata 15 SE.

In order to have the index values comparable to the entire study sample, data were pooled over all four waves for calculation of the index. In order to ensure that each CU contributed equally to the calculation of the wealth distribution for the study area, CU-level weights were calculated based on the following formula:

(1) ${weight}_{ik}= \left( \frac{N_{total}}{32} \right)/N_{ik}$

Where i=1,….,32 indicates CU and k=1, 2, 3 indicates 6-months, 12-months, and 18-months respectively. N_total_ represents total number of fevers while $N_{ik}$ represents the number of fevers in CU i at time point k.

The first principal component accounted for 37.3% of the total variance. Distribution of scores for the first principal component can be seen below in Figure 1.

1 Rutsein, S. O. & Johnson, K. The DHS Wealth Index. (Calverton, Maryland, USA, 2004).

2 Kolenikov, S. & Angeles, G. Socioeconomic Status Measurement with Discrete Proxy Variables: Is Principal Component Analysis a Reliable Answer? *Review of Income and Wealth* **55**, 128-165, doi:10.1111/j.1475-4991.2008.00309.x (2009).

3 Kolenikov, S. & Ángeles, G. The use of discrete data in principal component analysis with applications to socio-economic indices. CPC. (MEASURE Working paper no. WP-04-85, 2004).

**Supplementary** Table 1. Variables Included in Construction of Wealth Index^1^

| **Questions** | **Full list of responses** | **Reduced Categories^2^** |
| --- | --- | --- |
| **Main source of drinking water for your household:** | (1) Piped water/Public Tap/borehole (2) Unprotected well (3) Protected well (4) Protected Spring (5) Unprotected Spring (6) Rain water (7) River water (8) Other:_______ | (1) Unprotected: Unprotected well, unprotected spring, river water  (2) Protected: Piped water/Public Tap/borehole, protected well, protected spring, rain water, purchased water (other) |
| **Does your household have the following items:** |  |  |
| (1) Electricity? | Yes/No | Yes/No |
| (2) A television? | Yes/No | Yes/No |
| (3) A refrigerator? | Yes/No | Eliminated: lack of variation |
| (4) A radio? | Yes/No | Yes/No |
| (5) A mobile phone (at least one member of the household has)? | Yes/No | Yes/No |
| (6) A motorcycle (at least one member of the household has)? | Yes/No | Yes/No |
| (7) A car/truck? | Yes/No | Eliminated: lack of variation |
| (8) A bank account (at least one member of the household has)? | Yes/No | Yes/No |
| **How many of the following livestock does your household have?** |  |  |
| 1) Cows | Integer number of cows | Any cows: Yes/No |
| 2) Sheep | Integer number of sheep | Any sheep: Yes/No |
| 3) Goats | Integer number of goats | Any goats: Yes/No |
| 4) Pigs | Integer number of pigs | Eliminated: lack of variation |
| **What kind of toilet does your household have?** | (1) Flush or pour flush toilet (2) VIP / Ventilated improved pit latrine (3) Pit latrine with slab (4) Pit latrine without slab (5) Composting toilet (6) Bucket toilet (7) No facility/bush/field (8) Other (Please specify): ________ | (1) Non-improved Latrine: Pit latrine without slab, bucket toilet, No facility/bush/field, Other  (2) Improved Latrine: Flush or pour flush toilet, VIP / Ventilated improved pit latrine, Pit latrine with slab, composting toilet |
| **What is the main material of the floor in your house?** | (1) Earthen  (2) Cement (3) Floor Tiles (4) Wood planks (5) Polished wood (6) Other (please specify) ________ | (1) Earthen: Earthen  (2) Hard Surface: Cement, Floor tiles, wood planks, polished wood, other |
| **What is the main material of the walls in your house?** | (1) Stone  (2) Brick (3) Timber (4) Iron Sheet (5) Mud  (6) Wood (7) Cement (8) Other (please specify)_________ | (1) Porous: timber, mud, wood  (2) Non-porous: stone, brick, iron sheet, cement |
| ^1^Index based on DHS Wealth Index questions and methods with some adjustments for binary variable distribution | | |
| ^2^Variables listed in order from lowest SES to highest SES | | |


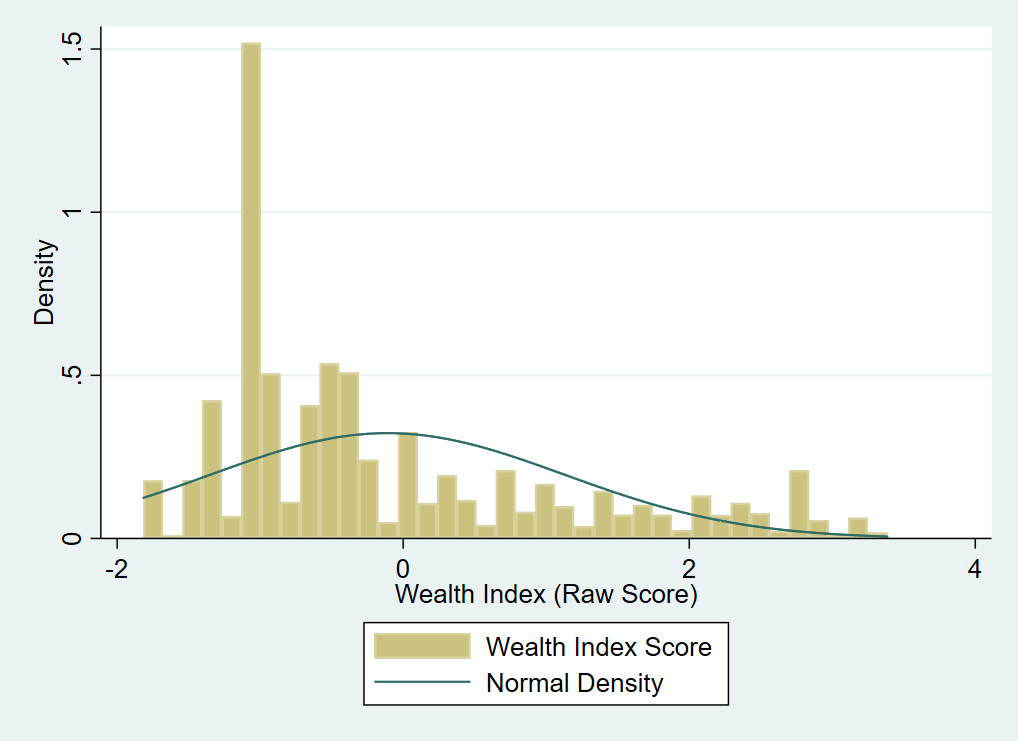


**Supplemental Figure 1.** Distribution of weighted wealth index scores over baseline, 6-, 12-, and 18-months
