# Supplementary material for: Improving rational use of ACTs through diagnosis-dependent subsidies: Evidence from a cluster-randomized controlled trial in western Kenya
Source: PLoS Med. 2018 Jul 17;15(7):e1002607. doi: 10.1371/journal.pmed.1002607 (PMC6049880; doi:10.1371/journal.pmed.1002607)
Supplement: S2 Text — (DOCX) [file pmed.1002607.s002.docx]

**Innovative Public-­private Partnership to Target Subsidized Antimalarials in the Retail Sector**

**Aim 2 Outcomes Paper**

**Statistical Analysis Plan**

**First Draft:** 5/23/2016

**Last Updated:** 10/9/2017

Contents

[Innovative Public-­private Partnership to Target Subsidized Antimalarials in the Retail Sector 1](#_Toc512852607)

[1. **Introduction** 3](#_Toc512852608)

[**2. Sample Size** 3](#_Toc512852609)

[**3. Randomization** 7](#_Toc512852610)

[**4. Study Population** 8](#_Toc512852611)

[4.1 Inclusion/Exclusion Criteria 8](#_Toc512852612)

[**4.1.1 INCLUSION CRITERIA** 8](#_Toc512852613)

[4.1.2 EXCLUSION CRITERIA 9](#_Toc512852614)

[**5. Study procedure:** 9](#_Toc512852615)

[5.1 Trial flow chart 10](#_Toc512852616)

[5.2 Timing of outcome measures 10](#_Toc512852617)

[**6. Primary, Secondary and Other Outcomes (see Table 1 for group definitions)** 10](#_Toc512852618)

[6.1.1 Primary Outcome Measures: 10](#_Toc512852619)

[6.1.2 Secondary Outcome Measures: 11](#_Toc512852620)

[6.1.3 Other Outcome Measures: 11](#_Toc512852621)

[6.2 Model Specification 11](#_Toc512852622)

[6.3 Outcome Definitions 13](#_Toc512852623)

[6.4. Demographic and Other Characteristics 14](#_Toc512852624)

[6.5 Missing data 15](#_Toc512852625)

[6.6 Sub-Analyses and Examination of Subgroups 15](#_Toc512852626)

[6.7 Statistical/analytical issues 15](#_Toc512852627)

[6.7.1 Adjustments for Covariates 15](#_Toc512852628)

[6.7.2 Imbalance in number of observations per CU 15](#_Toc512852629)

[**Appendix A: CONSORT flow-chart for progress of individuals through four treatment groups**  17](#_Toc512852630)

[**Appendix B:** 19](#_Toc512852631)

[**Appendix C. Shell tables for main analysis** 21](#_Toc512852632)

[**Appendix D: Wealth Index** 26](#_Toc512852633)

[**Appendix E. Example Sampling Plan from Bokoli Division)** 31](#_Toc512852634)

[**References** 32](#_Toc512852635)

[**Amendments to Analysis** 33](#_Toc512852636)

# **1. Introduction**

The overall objective of this study is to evaluate the public health impact of targeted antimalarial subsidies through scale-up by determining the community-wide effects of targeting an antimalarial subsidy through a partnership between CHVs and the private retail sector. We will use a cluster-randomized design using established community health volunteers (CHVs) in both areas. The conditional subsidy will be offered in the form of a voucher providing for the purchase of a WHO-qualified ACT at a reduced, fixed price to those with a positive malaria test that can be redeemed at a local drug retailer.

The **primary outcome** of this study is to compare the percent of fevers that receive a malaria test from any source between the intervention and control arms.

The **secondary outcomes** of this study will also be measured and compared between intervention and control arms. The main secondary outcome is the percent of all ACTs used that were taken by people with a malaria positive test. Additional secondary outcomes are: the percent of all ACTs used that were taken by people without a test, the percent of those with a positive test who got an ACT, and the percent of those with a negative test who got an ACT.

Aim 2 of IPPP is a cluster-randomized where community units (CUs) are the clusters to be randomized to either an intervention or control arm. The study will be carried out in two sub-counties in Kenya with active community health worker programs. Community units consist of 1000 households (approximately 5000 people), 10-20 CHVs and One Community Health Extension Worker (CHEW). Each CHV is responsible for a specific set of 50-100 households while the CHEW supervises the 10 CHVs. There are 33 CUs with existing community health workers in total across both sub-counties, 19 in Bungoma East, and 16 in Kiminini.

This trial was registered at [clinicaltrials.gov](https://clinicaltrials.gov/) as [NCT02461628](https://clinicaltrials.gov/ct2/show/NCT02461628?spons=Duke+University&cntry1=AF%3AKE&rank=2).

# **2. Sample Size**

The study will survey 40 people with fever in the previous 4 weeks in each CU at each of the 4 measurement time points (baseline, 6, 12 and 18 months post-baseline). With 16 CU per arm, this will total 640 fevers per arm at each time point for a total of 1280 across both arms. We anticipate that approximately 22% of households will have a member with a self-reported fever within the previous 4 weeks, so that the study team outcome assessors will need to visit 2883 households per arm at each measurement time point. All endpoints will be evaluated at 6 months post-baseline to determine whether high coverage of testing can be scaled-up quickly. Evaluation of the endpoint at 12 and 18 months will measure the saturation level of the intervention.

Based on pilot data from Bungoma, we anticipate that the proportion of fevers in the past 4 weeks who underwent testing for malaria (i.e. our primary outcome) will be 70% vs. 31% in intervention vs. control arms. These assumptions together with other assumptions are shown in Figure 2. Specifically, pilot data suggests that 43% of tested fevers will be positive (with no difference between arms). Of those with a positive test, 90% vs. 70% will purchase ACT in the intervention arm vs. control arm. Only 10% of those with a negative test are assumed to purchase ACTs (at the retail price) in both arms. Of those without a test, pilot data indicates that 21% purchase an ACT, and we expect this to be the same in both arms. Based on these assumptions, Table 1 shows the expected proportions for each of our primary and secondary outcomes and the number of fevers per CU which will be included in the denominator of each outcome (since all our secondary outcomes are conditional on testing or on ACT use so that the denominators are smaller than our assumed 40 fevers per CU). The table also reports the anticipated power of the comparisons. Details of the rationale and other assumptions of power calculations are listed below. Changes in power for our key outcomes under varying assumptions and scenarios can be found in Appendix B.

**Figure 1: Participant Flow and assumed probabilities.**

**Note that percentages on the branches of the tree are probabilities conditional on the previous step. Percentages at the end of the branch represent overall percentages out of 100% for each arm.**


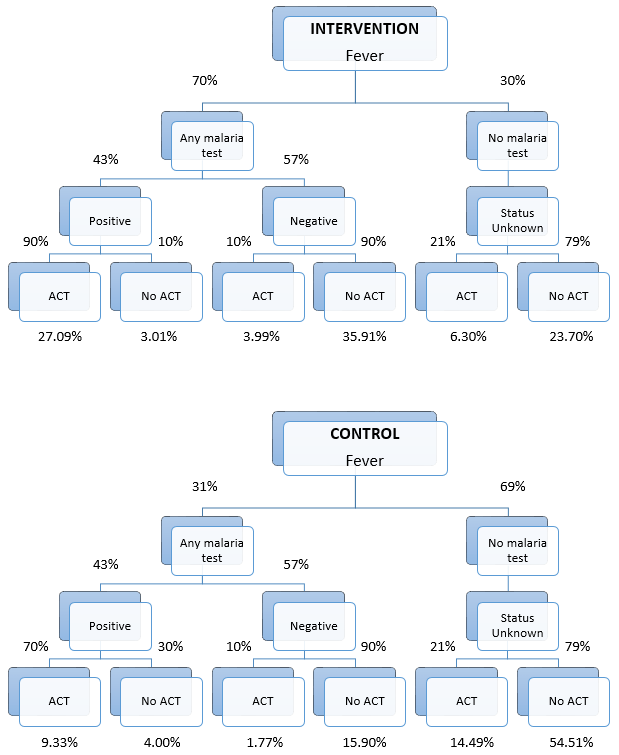


We calculated power based on a cluster randomized two-sample two-tailed t-test for the comparison of two proportions using standard formulae, which use the coefficient of variation as the measure of between-cluster variability (Hayes & Moulton, 2009). Specifically, we calculated power for the two-tailed comparison of the intervention arm vs. the control arm for the primary outcome of the proportion of clients with fever in the previous four weeks who reported being tested for malaria prior to any treatment. To ensure that our overall two-tailed Type I error (alpha) was 5%, we fixed the alpha level at 1.667% (i.e. 5%/3) for each of the 3 follow-up time points (6, 12 and 18 months post-baseline), using the conservative Bonferroni correction (Aickin & Gensler, 1996)[1]. In practice, we expect some dependency between tests of the treatment effect at different time points, therefore Bonferroni is too conservative. Instead, we will use the less conservative Benjamini Hochberg procedure^[[1]](#footnote-1)^ to adjust define the appropriate alpha level for simultaneously testing the primary outcome at those 3 time-points [2]. This procedure is more powerful than the traditional Bonferroni correction while controlling the false discovery rate under positive dependency between test statistics [3]. To further protect against possible losses in power due to the stratified study design, we conservatively based the power on a matched-cluster design, as the stratified design is expected to have more power than a matched design.

**Table 1: Summary of study outcomes, assumed effects, denominators per CU, and power at each of 3 follow-up time points for a cluster-randomized trial of 16 intervention CUs vs 16 control CUs at overall 5% type-1 error rate for each outcome (Bonferroni correction for 3 time points)**

| **Assumptions** | |
| --- | --- |
| **A. % fever with test** | 70% vs. 31% |
| **B. % +ve test** | 43% vs. 43% |
| **C. % take AL if +ve** | 90% vs. 70% |
| **D. % take AL if -ve** | 10% vs. 10% |
| **E. % take AL if no test** | 21% vs. 21% |
| **Outcome** | **INT vs. control** |
|  | ICC**; Assumed n per cluster (set at min of intervention and control); Power** |
| **A. % fevers with test**  *(Primary Outcome 1)* | 70% vs. 31% |
|  | 0.073; 40; 98% |
| **% +ve of all ACT taken**  *(Secondary Outcome 1)* | 72.5% vs. 36.5% |
|  | 0.017; 10; >99% |
| **% no test of all ACT taken**  *(Secondary Outcome 2)* | 16.9% vs. 56.6% |
|  | 0.064; 10; >99% |
| **C. % take AL if +ve**  *(Other Outcome 1)* | 90% vs. 70% |
|  | 0.003; 5; 50% |
| **D. % take AL if –ve**  *(Other Outcome 2)* | 10% vs. 10% |
|  | 0.007; 7; - |
| **% take AL overall**  *(Other Outcome 3)* | 37% vs. 26% |
|  | 0.012; 40; 84% |
| **% Targeted AL Use (all fevers)**  *(Secondry Outcome 4)* | 63% vs. 28% |
|  | 0.028; 40; >99% |
| **% Appropriate AL use (tested)**  *(Other Outcome 4)* | 90% vs. 90% |
|  | 0.007; 11; - |
| *Letters A – E denote assumptions we are making about the sample in order to calculate power. In some cases, the assumption itself is the outcome in which case we list the letter with that outcome | |

# **3. Randomization**

Community Units (CUs) in each sub-county were the units of randomization. A community unit consists of 1000 households (approximately 5000 people) 10 CHVs and One Community Health Extension Worker (CHEW). Each CHV is responsible for 100 households while the CHEW supervises the 10 CHVs. There are 35 CUs in total across both sub-counties, 19 in Bungoma East (with two sub-areas – Western BE, i.e. Bokoli (8 CUs), and Eastern BE, i.e. Ndivisi (11 CUs)) and 16 in Kiminini. One CU in Ndivisi was dropped to avoid odd numbers (CU dropped base on “last one added”). The 34 CUs were stratified within the three study areas based on whether there was a health facility located within its administrative borders. This created 5 strata in total.

Within each of the 5 strata, half of the community units were randomly allocated to the intervention and the remainder of the community units to the comparison arm (refer to Table 2). ^[[2]](#footnote-2)^ The randomization code is contained in the file *Randomization Coupon R01 August28th 2015.do*. Following randomization and baseline data collection (prior to implementation of the intervention) two CUs were found to be inelgibile for the intervention due to an absence of any known CHVs. Both CUs belonged to the Kiminini area and both belonged to the “no health facility” strata. This left a total of 32 CUs that were eligible to participate in the study.

Based on initial data, the study team expected to screen 2883 subjects for eligibility in each arm at each of 4 outcome assessment surveys (refer to Section 2 for calculation of sample size, refer to Section 5 for study procedures, including data collection) and we expect to enroll 640 assuming that 25% of households have had an individual with fever in the last one month. In order to obtain a random sample of 40 fevers per CU, we used systematic random sampling whereby households in the CU were visited according to a pre-specified sampling interval. Based on administrative rosters indicating the number of households in each CU and based on the assumed prevalence of P%, we calculated the sampling interval as follows:

$${Sampling Interval}_{i}= N_{i}/\left[ \frac{40}{P} \right]$$

where i=1,..,32 indicates CU, P is the expected prevalence of malaria, and $N_{i}$ is the number of households in CU i (derived from local administrative records). For additional details on how houses were sampled once the sampling interval was determined, see Appendix E “Sampling Plan.” For each survey round, as new prevalence information came about and as the malaria season progressed, the prevalence was adjusted to compute new CU sampling intervals.

We expect minimal contamination between the intervention and the control arm since CHVs are organized by geographic location and are responsible for specific households.

**Table 2. Sampling and stratification**

|  | **Health Facility?** | **Number of Community Units (CUs)** | | | | **Target number of subjects with fever in each of the 4 cross-sectional surveys^1^** | **Target number of subjects screened in each of the 4 cross-sectional surveys^2^** |
| --- | --- | --- | --- | --- | --- | --- | --- |
| **Arm** |  | Bungoma East | | Kiminini | Row Total |  |  |
|  |  | Ndivisi | Bokoli |  |  |  |  |
| **Intervention** | YES | 2 | 0 | 2 | 4 | 160 | 721 |
|  | NO | 3 | 4 | 5 | 12 | 480 | 2163 |
| **Comparison** | YES | 2 | 0 | 2 | 4 | 160 | 721 |
|  | NO | 3 | 4 | 5 | 12 | 480 | 2163 |
| **Total number** | | 10 | 8 | 14 | 32 | 1280 | 5768 |
| ^1^Number of CUs x 40 individuals per CU | | | | | | | |
| ^2^Number of subjects with fever based on assumed prevalence of 22.21% | | | | | | | |

# **4. Study Population**

Half of the community units in each study area (9 in Bungoma East and 7 in Kiminini) (Table 3) will be randomly selected to be included in the intervention arm. The remainder of the community units will be the comparison arm. CHVs are responsible for specific households and they know the household members in their allotted households.

## 4.1 Inclusion/Exclusion Criteria

### 4.1.1 INCLUSION CRITERIA

Subjects who meet all of the following inclusion criteria will be eligible to participate in the study

**Intervention participation criteria:**

- Client is older than 1 year
- Client has fever or history of fever or feeling unwell with a malaria-like illness within the last 2 days
- Client or their parent/legal guardian (if under 18) consents to participate

**Cross sectional survey participation criteria:**

- Household representative in the intervention or control arm
- At least one member in the respondent’s household with a history of fever or feeling unwell with a malaria like illness within the last four weeks
- Respondent is older than 18 years

### 4.1.2 EXCLUSION CRITERIA

**Intervention exclusion criteria:**

- Client has signs of severe disease or other problem requiring immediate referral to a health facility
- Client has already visited a health facility, taken or purchased antimalarials for the current illness.

**Cross sectional survey exclusion criteria:**

- Households not in the intervention or control arms

# **5. Study procedure:**

Following randomization, if a participant in the intervention arm decides to seek a test from the CHW, s/he will have to contact the CHW and request a test. After seeking written informed consent, the test will be performed by the CHW and the results recorded in a register. The CHW will interpret the test and explain the results to the participant. CHWs will keep the used RDT and give them to the field supervisor each week. The field supervisor will check the recorded results against the RDT to make sure the CHW correctly interpreted the test. Supervisors will also conducted random visits to observe a test being done to ensure adherence to proper procedures and study protocol.

The CHW will provide a written referral note for both positive and negative results that the participant can present to a health facility. The CHW will refer seriously ill patients to the facility.

If the participant has a positive RDT result, the CHW will provided a serialized voucher to the participant to be used within two days for a discount on a WHO-quality assured ACT (‘conditional subsidy’). The voucher can be redeemed at any nearby participating shop that stocks the correct ACTs. The subsidy level combined with the current government subsidy reduces the price down to the price of the drug under the original AMFm subsidy (<$0.50 USD), which is no longer available in Kenya.

At a follow up visit one week later, the CHV will revisit enrolled clients. The type and price of the drug(s) reported to have been purchased will be recorded for all customers regardless of whether they redeemed their voucher.

Data will be collected by a face-to-face interview at enrolment and follow-up. A single finger-prick blood sample (if an RDT is desired) will be collected to perform the RDT at a separate encounter with the CHW. Data will be entered into an electronic survey form on an encrypted, password protected netbook.

## 5.1 Trial flow chart

Data on the number of CUs randomized (with exclusions and reasons for exclusion), the flow of individuals through enrolment, allocation to intervention, follow-up (including withdrawals and the stage of the trial at which they occurred) and analysis will be presented in a flow chart [2]. See example CONSORT flow-chart in the Appendix.

## 5.2 Timing of outcome measures

**Figure 1. Study Timeline**

# **6. Primary, Secondary and Other Outcomes (see Table 1 for group definitions)**

All primary and secondary endpoints will be evaluated at 12 months post-baseline. Additional information will be provided by the 18-month timepoint

### 6.1.1 Primary Outcome Measures:

1. Comparison of percent of fevers that receive a malaria test from any source between arms.

### 6.1.2 Secondary Outcome Measures:

1. Percent of all ACTs used that were taken by people with a malaria positive test.
2. Percent of all ACTs used that were taken by people without a test
3. Percent of people that took ACTs and received a correct dose^[[3]](#footnote-3)^
4. The percent of people with correct AL targeting (“correct targeting” defined as positives taking AL OR negatives not taking AL over a denominator of all fevers)

### 6.1.3 Other Outcome Measures:

1. Percent that take AL if +ve
2. Percent that take AL if –ve
3. Percent that take AL overall
4. Percent with good malaria management of those tested (i.e. positives taking AL + negatives not taking AL / total tested)

## 6.2 Model Specification

All study outcome measures are individual-level binary outcome measures. To answer our primary research questions on the impact of the intervention on those binary outcomes, we will fit linear probability model (LPM) using a binomial distribution with an identity link. The LPM has the advantage of offering ease of interpretation of regression parameters (interpreted as absolute differences in proportions).^[[4]](#footnote-4)^ The main disadvantage of the LPM is that convergence can become an issue when predicted values fall outside of the [0, 1] interval. Our second preference for functional form for our regression models is the modified Poisson model to generate ratios of prevalences [5], followed by logistic regression, which would yield ratios of prevalence odds.The following criteria will be used to assess whether we will switch from LPM to modified Poisson or from modified Poisson to logistic:

- LPM (binomial model)
  - **Criteria 1**: Must converge with covariates for each of our main and secondary outcomes
  - **Criteria 2:** Sensitivity analyses must converge for unadjusted models
  - ***Both criteria must be met***
  - Modelling as separate time points does not alleviate convergence issue
- Modified Poisson model
  - Same as LPM criteria
- Logistic model
  - If all else fails

We will use generalized linear mixed models (GLMM) to account for clustering within CU and repeated measures over time (if needed if we randomly sample the same people at different follow-up time points). We will indicator variables for the presence of a health facility in a CU, an indicator of sub-county (Kimini, Bokoli, or Ndivisi), and their interaction as fixed effects to account for the stratification in our study design, and individual level weights (calculated to weight all CUs equally) to account for the study design. All analyses will be based on the intention-to-treat principle. Using our primary endpoint of testing prior to any treatment in those with fever as an example, each model takes the form:

- - 1. Yijk= β0 + β1interventioni + β2 T_12mth,_jk + β3 T_18mth,_jk + β4 interventioni X T_12mth,_jk + β5 interventioni x T_18mth,_jk + β_6_logit(Yi0) **+** β_7_Facilityi **+ SubCounty**i‘**κ+** Facilityi x **SubCounty**i ’**π** + Dijk + **w**ijk’**α**  + **c**i0’**δ**

where Y_ijk_ is indicator of whether participant j in CU i at follow-up time point k (j=1,…,40; i = 1…,32; k=1,2,3 corresponding to 6, 12 and 18 months post-baseline) took a malaria test; Intervention_j_ is an indicator of whether the jth CU is in the intervention arm (1= in arm, 0=not in arm), T_12mth,_jk and T_18mth,_jk are indicators for the jth CU at follow-up time point k, which indicate whether the time point is the 12 month or 18 month follow-up respectively, Facilityi indicates that there is a health facility in CU i, **SubCounty**i is an vector of indicators for location of a CU within Kimini, Bokoli, or Ndvisi; **w**ijk is a vector of potential confounder variables measured at each wave (e.g., season, age and distance to health facility) to account for imbalances between the 2 study arms; and yj0 is the baseline (i.e. pre-intervention) CU-level proportion of the outcome for the jth CU (i.e. jth cluster); We will consider including Dijk as an individual level fixed effect indicating which data collector surveyed the household of individual j in CU i at time point k, and **c**i0 is a vector of potential confounder variables measure for CU i at baseline. Regression diagnostics, including residual plots, will be used to verify model assumptions.

The model-estimated contrast of β3 + β5 represents the main outcome (the adjusted intervention effect at 18 months follow-up) while the contrast β2 + β4 represents our secondary outcome (the adjusted intervention effect at 18 months follow-up) (see Figure 1). If the LPM model is not the final chosen model, probability ratios (or log-probability odds ratios if logistic link is used) for the intervention vs. control arms can be estimated by exponentiating the parameters and deriving corresponding 95% confidence intervals.

Though using a mixed model approach with repeated cross-sectional surveys on the same clusters over time, it may exacerbate convergence issues that occur due to possible differences in prevalence of outcomes at different time points (e.g. very low prevalence of malaria in the dry season) . The gain in power from jointly modeling all follow-up time points is expected to be modest due to the cross-sectional nature of the data (i.e. we have repeated measures only at the cluster level, not at the individual level). This, coupled with the fact that our main outcome of interest is a between group difference at a specific time point, rather than the changes in outcome levels over time makes it prudent for us to consider modeling each time point separately as an alternative approach. We have therefore added this approach to our decision criteria for selection of modelling approach.

Our primary aim is to determine whether there is a significant difference between the 2 study arms in the proportion of clients with fever who are tested prior to any treatment at the 12-month time point, after adjusting for relevant covariates at each of the follow-up periods. This can be evaluated by testing the null hypothesis H0: β3 + β4=0. As a secondary aim we will examine the effect of the intervention at the 18-month follow-up, which will be determined by testing H0: β2 + β_5_ =0. As noted above in Section 2 (Sample Size), we will use the Benjamini Hochberg procedure for determining significance of the tests of the two contrasts together with the contrast at the 6-month time point. We will fit all models using meglm or xtlogit commands in Stata 14 (StataCorp, College Station, TX). We will also compare secondary measures using the same modeling approach.

## 6.3 Outcome Definitions

| **Table 4. Study Outcomes Derived from Survey Questions** | |
| --- | --- |
| **Outcomes** | **Survey questions used** |
| **Primary Outcome Measures:** |  |
| 1.       Comparison of percent of fevers that receive a malaria test from any source between arms. | *45. Did you have a malaria test? (1="Yes")* |
| **Secondary Outcome Measures:** |  |
| 1.       Percent of all ACTs used that were taken by people with a malaria positive test. | *44. Did you have a malaria test? (1="Yes") 50. What were the results of the malaria test? (1="Positive") 60. Which medicines did you take? (1=”Coartem/Artefan/other AL“ )  65. Did you start taking the Antimalarial before or after the malaria test? (2="After")* |
| 2.       Percent of all ACTs used that were taken by people without a test | *45. Did you have a malaria test? (2="No") 60. Which medicines did you take? (1=”Coartem/Artefan/other AL“ )* |
| 3.       Percent of people that took ACTs and received a correct dose* | *64. If AL, how many pills were given? If not AL, skip to 64*  *1=6*  *2=12*  *3=18*  *4=24*  *5=Don’t know/remember*  *6=OTHER: _____________*  *66. If AL, did you / patient complete the full course?*  *1=Yes*  *2=No*  *3=Not sure*  *4=Don’t remember*  *68. If AL, how many days to complete the course?* |
| 4.       The percent of people with fever that received correct treatment (“correct treatment” defined as positives taking AL OR negatives not taking AL) | *44. Did you have a malaria test? (1="Yes") 50. What were the results of the malaria test? (1="Positive") (2="Negative) 60. Which medicines did you take? (1=”Coartem/Artefan/other AL“ ) if q50 = 1 65. Did you start taking the Antimalarial before or after the malaria test? (2="After")* |
| **Other Outcome Measures:** |  |
| 1.       Percent that take AL if +ve | *44. Did you have a malaria test? (1="Yes") 50. What were the results of the malaria test? (1="Positive") 60. Which medicines did you take? (1=”Coartem/Artefan/other AL“ )  65. Did you start taking the Antimalarial before or after the malaria test? (2="After")* |
| 2.       Percent that take AL if –ve | *44. Did you have a malaria test? (1="Yes") 50. What were the results of the malaria test? (2="Negative") 60. Which medicines did you take? (1=”Coartem/Artefan/other AL“ )  65. Did you start taking the Antimalarial before or after the malaria test? (2="After")* |
| 3.       Percent that take AL overall | *60. Which medicines did you take? (1=”Coartem/Artefan/other AL“ )* |
| 4.       Percent with good malaria management of those tested (i.e. positives taking AL + negatives not taking AL / total tested) | *44. Did you have a malaria test? (1="Yes") 50. What were the results of the malaria test? (1="Positive") (2="Negative) 60. Which medicines did you take? (1=”Coartem/Artefan/other AL“ ) if q50 = 1 65. Did you start taking the Antimalarial before or after the malaria test? (2="After")* |

*Taken from *“C:\Users\acp18\Dropbox\Coupon R01\Field tools\Aim 2 Tools\Household Survey\Reduced survey for Feb 2016”*, all other questions from *“C:\Users\acp18\Dropbox\Coupon R01\Baseline training and tools\ revised survey tool_20150608”*

## 6.4. Demographic and Other Characteristics

We will compare key household characteristics between the intervention and control groups in order to ascertain balance between arms. In order to compare balance in SES, we will use principle components analysis to define a wealth index[6] (see Appendix C for more information on variables and method). No significance tests will be performed to test for differences at baseline. Descriptive statistics for continuous variables will include the mean, standard deviation, median, range and the number of observations. Categorical variables will be presented as numbers and percentages. Any demographic or SES variables expected to prognostic for study outcomes will be included in our regression analysis. Proposed demographic characteristics can be found in Table 1 of Appendix A. In addition, we will include individual level, time-varying fixed-effects for data collector in our main analysis. An F-test will determine whether data collector is an important predictor for each outcome in our adjusted regressions. If the null hypothesis, that there is no joint effect of data collector on our outcome is accepted, these fixed effects will be dropped from the analysis.

Geographic coordinates were collected for each household enrolled in the study, we will calculate geographically derived variables, such as distance to health facilities and market centers, to account for characteristics of the geographic area that may influence the outcome of testing and AL purchase but may not have been balanced via the cluster randomization.

## 6.5 Missing data

Because our outcomes are modelled using repeated cross-sectional survey data, missingness is defined simply as a household being absent or refusing to complete survey. We do not expect this issue to influence our study outcomes.

## 6.6 Sub-Analyses and Examination of Subgroups

Autonomy of testing and treatment decisions depend on the age of the febrile individual. It is possible that the effects of the intervention may differ based on age (i.e. children vs. adults). We will examine this possibility by testing the presence of an interaction effect between age and treatment group. Differences between sub-groups will be identified by the significance of the interaction effect. If outcomes do indeed differ by subgroup, we will report results separately by subgroup.

Sub-analysis will also be performed to examine the sensitivity of results to the precise definition of the outcome. The first set of sub-analyses will look at the set of main outcomes using only those malaria tests for which documentation was provided to the data collectors (i.e. duplicate form for those who tested at the CHV center, health booklet for those who tested at facilities).

A third sub-analysis will involve determining sensitivity of results to inclusion of covariates that add geographic context to health decisions. Covariates indicating distance to health facilities and distance to shops will be included to account for these contextual factors.

A final sub-analysis may be to look at the differences in awareness of health services in a CU by arm, pre and post intervention.

## 6.7 Statistical/analytical issues

### 6.7.1 Adjustments for Covariates

Though we will present unadjusted results for our main analysis, we will consider our adjusted regression results as our main results (i.e. equation (1) with covariates). We will perform adjusted regressions, using covariates thought to be related to the outcome (e.g. age, gender, education, SES). We will also use CU level adjustment variables to try to explain any existing relationship between CU and outcome.

### 6.7.2 Imbalance in number of observations per CU

We designed and powered our study to collect equal amounts of information by CU. In practice, we are unlikely to achieve perfect balance by CU. Therefore, we compute survey weights to correct for any imbalances in number of households surveyed per CU. Survey weights will be computed as follows:

${weight}_{ik}= \left( \frac{N_{total}}{32} \right)/N_{ik}$

Where i=1,….,32 indicates CU and k=0, 1, 2, 3 indicates baseline, 6-months, 12-months, and 18-months respectively. N_total_ represents total number of fevers while $N_{ik}$ represents the number of fevers in CU i at time point k.

Descriptive statistics and all regression analysis will include the above weights to account for imbalance between CUs. We will also present descriptive statistics as unweighted in order to characterize the individuals in the sample we ultimately collect.

# **Appendix A: CONSORT flow-chart for progress of individuals through four treatment groups**

**Appendix B: Power for 6 key outcomes for a two-group cluster randomized controlled trial design with a total of 32 clusters for the Coupon R01 Aim 2 trial updated with assumptions based on data from 1,997 participants in the baseline survey. Note that the calculations are at an overall two-tailed 5% significance level, with a Bonferroni correction for tests at three time points (i.e. significance level for each test of 0.016667) with original estimates from the IRB submission of CV based on assumptions about distribution of distribution of CU-specific proportions. Note that power was taken from column AA of sheet *CRT sample size OMeara R01 June 2015 Pre-Study FINAL1 SCENARIO ORIGINAL AP - 12-2-2015.xlsx.* USING ORIGINAL ICCs**

|  | **Scenario (**with differences from scenario 1 **highlighted)** | | | | |  |
| --- | --- | --- | --- | --- | --- | --- |
|  | **Original** | **1. All at pilot %** | **2. Like 1, except %+ve=43% INT** | **3. “Best case”:**  **Like 2, except in INT**  **%-ve with ACT = %no test with ACT = 50%** | **4. “Worst case”: Like 2, except in INT**  **%+ve take test = 70%** | **5. Intervention estimates via Aim 1, Control estimates via Aim 2 (baseline)** |
| **Assumptions** | **INT vs. control** | **INT vs. control** | **INT vs. control** | **INT vs. control** | **INT vs. control** | **INT vs. control** |
| **A. % fever with test** | 70% vs. 31% | 70% vs. 31% | 70% vs. 31% | 70% vs. 31% | 70% vs. 31% | 74% vs 44% |
| **B. % +ve test** | 43% vs. 43% | **69%** vs. **69%** | **43%** vs. 69% | **43%** vs. 69% | **43%** vs. 69% | 39% vs. 83% |
| **C. % take AL if +ve** | 90% vs. 90% | 90% vs. 90% | 90% vs. 90% | 90% vs. 90% | **70%** vs. 90% | 82% vs. 87% |
| **D. % take AL if -ve** | 10% vs. 10% | **70%** vs. **70%** | 70% vs. 70% | **50%** vs. 70% | 70% vs. 70% | 28% vs. 53% |
| **E. % take AL if no test** | 21% vs. 21% | **80%** vs. **80%** | 80% vs. 80% | **50%** vs. 80% | 80% vs. 80% | 30% vs. 67% |
| **Outcome** | **INT vs. control**  **ICC; Assumed n per cluster (set at min of intervention and control); Power** | | | | | |
| **Primary Outcome 1 (A)** | 70% vs. 31%  0.073; 40; 98% | | | | | 74% vs. 44%  0.043; 40; >99% |
| **% +ve of all ACT taken** | 72% vs. 36%  0.027; 10; 90% | 53% vs. 24%;  0.027; 32; 98% | 34% vs. 24%  0.027; 32; 36% | 44% vs. 24%  0.027; 25; 81% | 29% vs. 24%  0.027; 29; 9% | 54% vs. 43%  0.093; 18; 14% |
| **% no test of all ACT taken** | 17% vs. 57%  0.064; 10; 100% | 29% vs. 68%;  0.064; 32; >99% | 30% vs. 68%  0.064; 32; >99% | 24% vs. 68%  0.064; 25; >99% | 33% vs. 68%  0.064; 29; >99% | 18% vs. 51%  0.078; 18; >99% |
| **C. % take AL if +ve** | 90% vs. 70%  0.074; 5; 50% | 90% vs. 90%;  0.074; 9; - | 90% vs. 90%  0.074; 9; - | 90% vs. 90%  0.074; 9; - | 70% vs. 90%  0.074; 9; 62% | 82% vs. 87%  0.12; 12; 6.5% |
| **D. % take AL if -ve** | 10% vs. 10%  0.007; 7; - | 70% vs. 70%;  0.007; 4, - | 70% vs. 70%  0.007; 4, - | 50% vs. 70%  0.007; 4; 40% | 70% vs. 70%  0.007; 4; - | 28% vs. 53%  0.37; 3; 27.2% |
| **% take AL overall** | 37% vs. 26%  0.012; 40; 84% | 83% vs. 81%;  0.012; 40; 2% | 79% vs. 81%;  0.012; 40; 2% | 62% vs. 81%;  0.012; 40; 86% | 73% vs. 81%;  0.012; 40; 16% | 44% vs. 73%  0.062; 40; >99% |

# **Appendix C. Shell tables for main analysis**

| **Table 1. Baseline Sample Characteristics by Treatment Group – n (%), unless otherwise noted'** | | | |
| --- | --- | --- | --- |
|  | Control (N=?) | Intervention (N=?) | Total (N=?) |
|  |  |  |  |
| **Adjustment Covariates** |  |  |  |
| **Age (Patient)** |  |  |  |
| Missing |  |  |  |
| Under 5 |  |  |  |
| 5 to 17 |  |  |  |
| 18+ |  |  |  |
| **Sex (Patient)** |  |  |  |
| Missing |  |  |  |
| Male |  |  |  |
| Female |  |  |  |
| **Household size (No. persons)** |  |  |  |
| Median |  |  |  |
| Q1, Q3 |  |  |  |
| **Any Mosquito Nets (Household)?** |  |  |  |
| Missing |  |  |  |
| Yes |  |  |  |
| No |  |  |  |
| **Slept under a net last night (patient)?** |  |  |  |
| Missing |  |  |  |
| Yes |  |  |  |
| No |  |  |  |
| **DHS Wealth Index (Quintiles)** |  |  |  |
| Missing |  |  |  |
| 0-20th |  |  |  |
| >20 - 40th |  |  |  |
| >40 - 60th |  |  |  |
| >60 - 80 |  |  |  |
| >80th |  |  |  |
| **Education (Respondent)** |  |  |  |
| Missing |  |  |  |
| None or less than primary |  |  |  |
| Completed primary |  |  |  |
| Completed secondary |  |  |  |
| Above secondary |  |  |  |
| **Cluster Level Covariates** |  |  |  |
| Has health facility |  |  |  |
| Number of participating shops |  |  |  |
| Number of households |  |  |  |
| Proportion of households with recent fever** |  |  |  |
| **Outcomes** |  |  |  |
| **Had malaria test** |  |  |  |
| Missing |  |  |  |
| No |  |  |  |
| Yes |  |  |  |
| **Malaria test result** |  |  |  |
| Missing |  |  |  |
| Negative |  |  |  |
| Positive |  |  |  |
| **Patient took AL** |  |  |  |
| Missing |  |  |  |
| No AL |  |  |  |
| AL |  |  |  |
| **Negative Test: Took AL** |  |  |  |
| Missing |  |  |  |
| No |  |  |  |
| Yes |  |  |  |
| **Positive Test: Took AL** |  |  |  |
| Missing |  |  |  |
| No |  |  |  |
| Yes |  |  |  |
| **No Test: Took AL** |  |  |  |
| Missing |  |  |  |
| No |  |  |  |
| Yes |  |  |  |
| **Appropriate AL Use** |  |  |  |
| Missing |  |  |  |
| No |  |  |  |
| Yes |  |  |  |
| **Targeted AL Use** |  |  |  |
| No |  |  |  |
| Yes |  |  |  |
| *Individual level means weighted to account for imbalance between CUs  **Mean (SD) | | | |

| **Table 2. Sample Proportions for testing and treatment outcomes and behavior for N=? participants in 32 clusters by study group** | | | | | | |
| --- | --- | --- | --- | --- | --- | --- |
|  | **Baseline** | | | **18-months** | | |
|  | **Control** | **Intervention** | **Total** | **Control** | **Intervention** | **Total** |
|  | **N=?** | **N=?** | **N=?** | **N=?** | **N=?** | **N=?** |
|  | % | % | % | % | % | % |
| **Had a malaria test^§^** |  |  |  |  |  |  |
| Positive |  |  |  |  |  |  |
| No ACT |  |  |  |  |  |  |
| ACT |  |  |  |  |  |  |
| Correct dose |  |  |  |  |  |  |
| Incorrect dose |  |  |  |  |  |  |
| Negative |  |  |  |  |  |  |
| No ACT |  |  |  |  |  |  |
| ACT |  |  |  |  |  |  |
| Appropriate ACT use* |  |  |  |  |  |  |
| **Did not have a test** |  |  |  |  |  |  |
| No ACT |  |  |  |  |  |  |
| ACT |  |  |  |  |  |  |
| **Targeted ACT use**** |  |  |  |  |  |  |
| **Observations** |  |  |  |  |  |  |
| **^§^** Self-report of any malaria test (RDT or slide) at health facility or tested by the CHW | | | | | | |
| * Defined as taking ACT if positive or not taking ACT if negative amongst those who had a malaria test | | | | | | |
| ** Defined as taking ACT if positive or not taking ACT if negative amongst all participants | | | | | | |

| **Table 3. Model estimated between group differences in testing between recipients of free RDT testing and conditional ACT subsidies vs. usual care in malaria testing behavior, defined as self-report of taking any malaria test^§^, for n=? study participants in k=32 CUs** | | |
| --- | --- | --- |
|  | Unadjusted | Adjusted |
| **Baseline** |  |  |
| Control |  |  |
| Intervention |  |  |
| **6-months** |  |  |
| Control |  |  |
| Intervention |  |  |
| **12-months** |  |  |
| Control |  |  |
| Intervention |  |  |
| **18-months** |  |  |
| Control |  |  |
| Intervention |  |  |
| **^§^** Self-report of any malaria test (RDT or slide) at CHW or health facility | | |
| *Interpreted as baseline risk (odds) for the control group (ref) | | |
| **Estimates weighted to account for imbalance in number of observations per CU | | |
| ***Estimates interpreted as risk ratios |  |  |
| *Fully adjusted model includes age (of patient), gender (of patient), education level (of patient or guardian if patient <18years), occupation (of patient or guardian), household size, wealth, and CU.* | | |

| **Table 4. Model estimated between group differences in AL use between recipients of free RDT testing and conditional ACT subsidies vs. usual care in 32 CUs** | | | | | | | | | | |
| --- | --- | --- | --- | --- | --- | --- | --- | --- | --- | --- |
|  | **Any AL Use** | | | | | | | | **Targeted AL Use** | |
|  | Unadjusted | Adjusted | Unadjusted | Adjusted | Unadjusted | Adjusted | Unadjusted | Adjusted | Unadjusted | Adjusted |
| **Baseline** |  |  |  |  |  |  |  |  |  |  |
| Control |  |  |  |  |  |  |  |  |  |  |
| Intervention |  |  |  |  |  |  |  |  |  |  |
| **6-months** |  |  |  |  |  |  |  |  |  |  |
| Control |  |  |  |  |  |  |  |  |  |  |
| Intervention |  |  |  |  |  |  |  |  |  |  |
| **12-months** |  |  |  |  |  |  |  |  |  |  |
| Control |  |  |  |  |  |  |  |  |  |  |
| Intervention |  |  |  |  |  |  |  |  |  |  |
| **18-months** |  |  |  |  |  |  |  |  |  |  |
| Control |  |  |  |  |  |  |  |  |  |  |
| Intervention |  |  |  |  |  |  |  |  |  |  |
| Observations |  |  |  |  |  |  |  |  |  |  |
| Sample | Positive | | Negative | | No Test | | All fevers | | All fevers | |
| *Interpreted as baseline risk (odds) for the control group (ref) | | | | | | | | | | |
| **Estimates weighted to account for imbalance in number of observations per CU | | | | | | | | | | |
| ***Estimates interpreted as risk ratios | | | | | | | | | | |
| *Fully adjusted model includes age (of patient), gender (of patient), education level (of patient or guardian if patient <18years), occupation (of patient or guardian), household size, wealth, and CUs.* | | | | | | | | | | |

| **Table 5. Model estimated between group differences in appropriate ACT use between recipients of free RDT testing and conditional ACT subsidies vs. usual care on among (n=?) users in 32 CUs** | | | | | | |
| --- | --- | --- | --- | --- | --- | --- |
|  | **Outcomes** | | | | | |
|  | **Malaria Positive** | | **Untested** | | **Positives Receiving Correct Dosage** | |
|  | Unadjusted | Adjusted | Unadjusted | Adjusted | Unadjusted | Adjusted |
| **Baseline** |  |  |  |  |  |  |
| Control |  |  |  |  |  |  |
| Intervention |  |  |  |  |  |  |
| **6-months** |  |  |  |  |  |  |
| Control |  |  |  |  |  |  |
| Intervention |  |  |  |  |  |  |
| **12-months** |  |  |  |  |  |  |
| Control |  |  |  |  |  |  |
| Intervention |  |  |  |  |  |  |
| **18-months** |  |  |  |  |  |  |
| Control |  |  |  |  |  |  |
| Intervention |  |  |  |  |  |  |
| *Interpreted as baseline risk (odds) for the control group (ref) | | | | | | |
| **Estimates weighted to account for imbalance in number of observations per CU | | | | | | |
| ***Estimates interpreted as risk ratios | | | | | | |
| *Fully adjusted model includes age (of patient), gender (of patient), education level (of patient or guardian if patient <18years), occupation (of patient or guardian), household size, wealth, and CU of residence.* | | | | | | |

# **Appendix D: Wealth Index**

Frequency tables will be created for each of the indicators in the wealth index. Sparsity in categories will be improved by collapsing categories into meaningful dimensions (e.g. wall materials may be grouped into “porous” and “non-porous” categories; roof type may be collapsed into thatched vs. not). Once meaningful categories are created (or confirmed) for each of the categorical variables, binary indicators will be created to represent each “category.” Continuous variables (9.3, 9.9, 1.5) will be kept in their continuous format. A polychoric correlation matrix will be construct for the final set of variables and the correlation matrix used in the subsequent factor analysis retaining only the first factor. Finally, a factor score will be computed for each household to complete the wealth index.

See below Table D for a list of the variables to be used in constructing the wealth index.

| **Table D. Variables Used for DHS-style Wealth Index** | | | |
| --- | --- | --- | --- |
| **Question #** | **Text** | **Choices** | **Type** |
| 101 | Main source of drinking water for your household: | *Piped water/Public Tap/borehole *Unprotected well *Protected well *Protected Spring *Unprotected Spring *Rain water *River water *Other:_______ | Multiple Choice (Binary) |
| 102 | Does your household have the following items: |  |  |
|  | a) Electricity? | Yes/No | Binary |
|  | b) A television? | Yes/No | Binary |
|  | c) A refrigerator? | Yes/No | Binary |
|  | d) A radio? | Yes/No | Binary |
|  | e) A mobile phone (at least one member of the household has)? | Yes/No | Binary |
|  | f) A motorcycle (at least one member of the household has)? | Yes/No | Binary |
|  | g) A car/truck? | Yes/No | Binary |
|  | h) A bank account (at least one member of the household has)? | Yes/No | Binary |
| 103 | How many of the following livestock does your household have? |  |  |
|  | a) Cows |  | Integer |
|  | b) Sheep |  | Integer |
|  | c) Goats |  | Integer |
|  | d) Pigs |  | Integer |
| 104 | What kind of toilet does your household have? | *Flush or pour flush toilet *VIP / Ventilated improved pit latrine *Pit latrine with slab *Pit latrine without slab *Composting toilet *Bucket toilet *No facility / bush / field *Other (Please specify): _________ | Multiple Choice (Binary) |
| 105 | What type of fuel does your household mainly use for cooking? | *Liquefied petroleum gas *Paraffin/Kerosene *Charcoal *Firewood *Dung *Biogas *Crop residue *Other (Specify)____________ | Multiple Choice (Binary) |
| 106 | What is the main material of the floor in your house? | *Earthen  *Cement *Floor Tiles *Wood planks *Polished wood *Other (please specify) __________ | Multiple Choice (Binary) |
| 107 | What is the main material of the walls in your house? | *Stone  *Brick *Timber *Iron Sheet *Mud  *Wood *Cement *Other (please specify)_____________ | Multiple Choice (Binary) |
| 9.8 | What is the main material of the roof of your house? | *Iron sheets  *Roof tiles *Grass Thatched *Wood *Other (please specify)________ | Multiple Choice (Binary) |
| 9.9 | How many acres/hectares/feet of land for farming does your household own? | *None  *Acres: _____________  *Hectares: _________  *Square feet(xx by xx):__________ | Integer |
| 21 | Total number of household members including respondent or patient: |  | Integer |

# **Appendix E. Example Sampling Plan from Bokoli Division)**

Information

- 8 clusters
- 750-1500 households per cluster

Study design

- Need 40 fevers per cluster.
- Assume screen about 200 households.

Average sampling interval 4-5 households

Procedure (sampling interval = 5)

1. Start at the village elder’s house
2. Roll a die
3. Start at the first, second, third, fourth or fifth house from the village elder’s house based on the number on the die. If you rolled a six, treat this as a zero and start at the elder’s home
4. Proceed in the most northerly direction possible until the boundary is reached, sampling every Nth household. Place a discreet chalk mark on fence or doorframe of each sampled household.
5. Return to the starting point and proceed in the same manner in the other three compass directions until you have covered the entire village.
6. Note how many households you sampled in that village
7. When the enumerators arrive, they should visit every marked household. If no one is home, ask when they can return. Try three times.

# **References**

1. Aickin, M. and H. Gensler, *Adjusting for multiple testing when reporting research results: the Bonferroni vs Holm methods.* American journal of public health, 1996. **86**(5): p. 726-728.

2. Benjamini, Y. and Y. Hochberg, *Controlling the false discovery rate: a practical and powerful approach to multiple testing.* Journal of the Royal Statistical Society. Series B (Methodological), 1995: p. 289-300.

3. Benjamini, Y. and D. Yekutieli, *The control of the false discovery rate in multiple testing under dependency.* Annals of statistics, 2001: p. 1165-1188.

4. Cheung, Y.B., *A Modified Least-Squares Regression Approach to the Estimation of Risk Difference.* American Journal of Epidemiology, 2007. **166**(11): p. 1337-1344.

5. Zou, G., *A Modified Poisson Regression Approach to Prospective Studies with Binary Data.* American Journal of Epidemiology, 2004. **159**(7): p. 702-706.

6. Rutsein, S.O. and K. Johnson, *The DHS Wealth Index*, in *DHS Comparative Reports*. 2004: Calverton, Maryland, USA.

# **Amendments to Analysis**

6/29/2017 – Wealth index constructed using only variables available in all 4 waves (assets, animals, floor, and wall materials); Binary splits created for floor and wall; binary indicator for each animal used due to overdispersion. Assets with cells less than 5% dropped.

8/8/2017 – Analytic team decides that it is important to generate both risk differences AND relative risk (previous SAP only specifies one or the other). Binomial risk model chosen for risk differences and modified Poisson for relative risk due to stability of estimation. Binomial risk converges for all but 2 outcomes (adjusted AL use in positives and adjusted AL use in negatives.

8/17/2017 – Selection of KC correction for finite sample correction after diagnostic analysis and comparison with FG correction.

9/18/2017 – Analytic team has decided that the wealth index should be pooled over all waves in order to be: (1) more stable; (2) representative of wealth over the entire study.

9/22/2017 – Analytic team chooses to use OLS with robust standard errors to generate risk differences for adjusted models that don’t converge (AL use in positives and AL use in negatives). For sensitivity, OLS models are generated for ALL outcomes and estimates + 95% CIs compared. Predicted values outside of interval [0,1] are calculated for AL use in positives and AL use in negatives (very small percentages) all leading to much more confidence that OLS with robust standard is giving us good estimates of risk differences for these outcomes.

10/9/2017 – Review of definition of outcomes measures, particularly as they apply to chronology of AL use (i.e. before or after test). Consensus reach about logical pathways to each study outcome (see Diagram below).


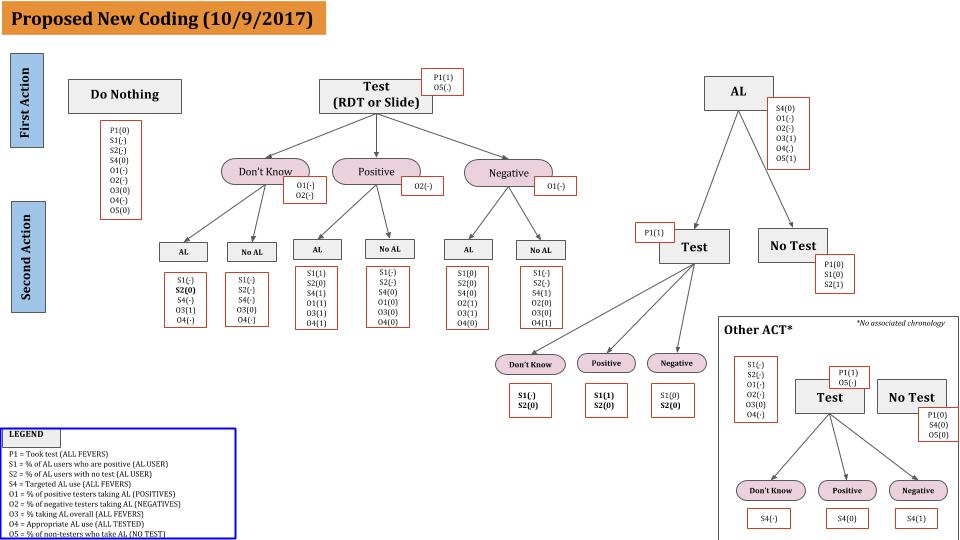


1. The Benjamini Hocherg Procedure: Let $p_{(1)}\leq p_{\left( 2 \right)}\leq\cdot\cdot\cdot\leq p_{(m)}$. Define: k$=max\left\{ i: p_{(i)}\leq\frac{i}{m}q \right\}$ , where m is the total number of hypothesis and q is the chosen false discovery rate (in our case, 0.05). Reject $H_{(1)} ... H_{(k)}$. If no such i exists, reject no hypothesis, An additional modification can be made to ensure wider breadth of dependency between tests is account for, simply by replacing q with $q/\left( \sum_{i=1}^{m} 1/i \right)$. [↑](#footnote-ref-1)
2. We dropped two in Kiminini after baseline but BEFORE intervention because they were found NOT to have CHVs and so violated the inclusion criteria. Fortunately, one was intervention and one was control and they were both from the same strata. [↑](#footnote-ref-2)
3. Note: There is no dosage information collected at baseline. All comparisons for this outcome will be made between groups at specific time points (12 and 18 months). [↑](#footnote-ref-3)
4. We may also want to consider using a modified least squares approach as specified by Cheung (2007).4. Cheung, Y.B., *A Modified Least-Squares Regression Approach to the Estimation of Risk Difference.* American Journal of Epidemiology, 2007. **166**(11): p. 1337-1344. [↑](#footnote-ref-4)
