# Supplementary material for: Improving rational use of ACTs through diagnosis-dependent subsidies: Evidence from a cluster-randomized controlled trial in western Kenya
Source: PLoS Med. 2018 Jul 17;15(7):e1002607. doi: 10.1371/journal.pmed.1002607 (PMC6049880; doi:10.1371/journal.pmed.1002607)
Supplement: S1 Protocol — (PDF) [file pmed.1002607.s003.pdf]

Innovative public-private partnership to target subsidized antimalarials in the retail sector

DMID Protocol Number:
15-0022

Sponsored by:
National Institute of Allergy and Infectious Diseases (NIAID)

DMID Funding Mechanism:

Principal Investigator:
Wendy Prudhomme O'Meara

DMID Program officer:

Malla Rao, DrPH, MEng

Draft or Version Number:
1.6

Day Month Year
23 June 2016

Statement of Compliance

The study will be carried out in accordance with the principles set forth in The Council for International Organizations of Medical Sciences (CIOMS) International Ethical Guidelines for Biomedical Research Involving Human Subjects.

SIGNATURE PAGE

The signature below constitutes the approval of this protocol and the attachments, and provides the necessary assurances that this trial will be conducted according to all stipulations of the protocol, including all statements regarding confidentiality, and according to local legal and regulatory requirements and applicable US federal regulations and ICH guidelines.

Site Investigator:

Signed: ____ Date: ________________

Name
Title

Table of Contents

page

Statement of Compliance i
Signature Page ii
List of Abbreviations iv
Protocol Summary v

1 Key Roles 2
2 Background Information and Scientific Rationale 4
 2.1 Background Information 4
 2.2 Scientific Rationale 5
 2.3 Potential Risks and Benefits 6
  2.3.1 Potential Risks 6
  2.3.2 Known Potential Benefits 7
3 Objectives 8
4 Study Design 9
5 Study Population 13
 5.1 Selection of the Study Population 13
 5.2 Inclusion/Exclusion Criteria 13
6 STUDY PROCEDURES/EVALUATIONS 15
 6.1 Study Procedures 15
 6.2 Laboratory Evaluations 19
  6.2.1 Laboratory Evaluations/Assays 19
  6.2.2 Specimen Collection, Preparation, Handling and Shipping 19
7 Statistical Considerations 20
 7.1 Study Outcome Measures 20
 7.2 Sample Size Considerations 22
 7.3 Participant Enrollment and Follow-Up 25
 7.4 Analysis Plan 25
8 Subject Confidentiality 27
 8.1 Future Use of Stored Specimens 27
9 Informed Consent Process 28
 9.1 Informed Consent/Assent Process (in Case of a Minor or Others Unable to Consent for Themselves) 29
10 Literature References 30

Table of Contents - *continued*

APPENDICES
Note: Files provided separately

Appendix 1: CHV client encounter documents
A. Client consent
B. Client assent (For children 8 years or older and younger than 18 years)
C. CHV encounter form
D. Voucher form

Appendix 2: Shop and Facility documents
A. Shops and facilities verbal consent
B. Shop and facility survey
C. Terms of Reference and written consent for shop participation in voucher program

Appendix 3: Household survey documents
A. Household survey verbal consent
B. Household survey instrument

Appendix 4: CHV evaluation documents
A. CHV observation checklist
B. CHV survey instrument
C. Verbal consent script for CHVs participating in CHV evaluation activities
D. Verbal consent script for clients participating in CHV evaluation activities (observation)

List of Abbreviations

ACT	Artemisinin Combination Therapy
AE	Adverse Event
AMFm	Affordable Medicines Facility - malaria
CFR	Code of Federal Regulations
CHV	Community Health Volunteer (also often called Community Health Worker)
CIOMS	Council for International Organizations of Medical Sciences
CRF	Case Report Form
DMID	Division of Microbiology and Infectious Diseases, NIAID, NIH, DHHS
DSMB	Data and Safety Monitoring Board
FWA	Federal-Wide Assurance
GCP	Good Clinical Practice
ICF	Informed Consent Form
ICH	International Conference on Harmonisation
IEC	Independent or Institutional Ethics Committee
IRB	Institutional Review Board
ISM	Independent Safety Monitor
JAMA	Journal of the American Medical Association
MOP	Manual of Procedures
N	Number (typically refers to subjects)
NEJM	New England Journal of Medicine
NIAID	National Institute of Allergy and Infectious Diseases, NIH, DHHS
NIH	National Institutes of Health
OCRA	Office of Clinical Research Affairs, DMID, NIAID, NIH, DHHS
OHRP	Office for Human Research Protections
ORA	Office of Regulatory Affairs, DMID, NIAID, NIH, DHHS

List of Abbreviations - *continued*

PI	Principal Investigator
RDT	Rapid Diagnostic Test for Malaria
SAE	Serious Adverse Event
SMC	Safety Monitoring Committee
SOP	Standard Operating Procedure
WHO	World Health Organization

Protocol Summary

Title: Innovative public-private partnership to target subsidized antimalarials in the retail sector

Population: This study will take place in Bungoma East and Kiminini sub-county in Kenya. Both are high malaria transmission areas but with different patterns of access to health services. The study population will be 20 community units in Bungoma East sub-county and 12 community units in Kiminini. Half of the community units in each study area (10 in Bungoma East and 6 in Kiminini) will be randomly selected to be included in the intervention arm. The remainder of the community units will be the comparison arm.

Number of Sites: (2) Bungoma East sub-county in Bungoma County, Kiminini sub-county in Trans Nzoia County, Kenya

Study Duration: 24 months (intervention period 18 months)

Subject Duration: Approximately 1 hour for each type of visit (3 possible visit types: 1) CHV visit; 2) Follow-up supervisor visit; 3) cross-sectional survey)

Objectives: Our overall objective is to evaluate the public health impact of targeted antimalarials subsidies through scale-up by determining the community-wide effects of targeting an antimalarial subsidy through a partnership between Community Health Volunteers (CHVs) and the private retail sector.

The **primary outcome** of this study is to compare the percent of fevers that receive a malaria test from any source between the intervention and control arms.

The **secondary outcomes** of this study will also be measured and compared between intervention and control arms. The main secondary outcome is the percent of all ACTs used that were taken by people with a malaria positive test. Additional secondary outcomes are: the percent of all ACTs used that were taken by people without a test, the percent of those with a positive test who got an ACT, and the percent of those with a negative test who got an ACT.

1 KEY ROLES

Individuals: DMID Representative:
Malla Rao, DrPH, M.Eng
DMID, NIAID
610 Rockledge Dr, MSC 6604, Room 5095
Bethesda MD 20892
301-451-3749
301-402-0659

MRao@niaid.nih.gov

Principal Investigator:

Wendy Prudhomme O'Meara, PhD

Assistant Professor of Medicine and Global Health

Duke University

Box 90519

Trent Hall, Durham, NC 27708

+254 728 306 521

wpo@duke.edu

Institutions: 1. Duke University

Box 90519 Durham, NC 27710

Darlene McCain

PHONE: 919 681 7717

FAX: 919 681 7677

2. Moi University School of Medicine

MTRH OPD Complex

Nandi Road

PO Box 4046

Eldoret 30100 KENYA

Christine Tonui, Research and Sponsored Projects Office

FAX: +254 532061195

PHONE: +254 532033471 ex. 3528

Cchuani@iukenya.org

2 BACKGROUND INFORMATION AND SCIENTIFIC RATIONALE

2.1 Background Information

In most malaria-endemic countries, a large fraction of fevers are treated in the informal health sector where diagnostic testing is uncommon and effective drugs are expensive. For many families, particularly in rural areas, the first source of treatment for fevers are retail medicine outlets such as chemists, pharmacists and small, unregulated medicine shops (Abuya, et al., 2007; Amin et al., 2003; Chuma et al., 2009; Malik et al., 2006; Littrell et al., 2011). These retail outlets, also referred to as the 'informal health sector', are more accessible than formal health services, but effective drugs are expensive and most clients purchase cheaper, ineffective therapies to which high levels of resistance exist. For patients with malaria, treatment failure is common and contributes to increased morbidity and mortality from this treatable disease. Fewer than 15% of fevers treated for malaria receive appropriate, effective therapy (World Health Organization, 2012).

Subsidized antimalarials in the retail sector improve access to effective drugs. The Global Fund piloted a drug subsidy called the Affordable Medicines Facility – malaria (AMFm) to reduce the prices of effective, high quality ACTs in the private sector. AMFm was launched in 2010 and provided quality-assured ACTs to wholesale markets at substantially reduced prices in seven pilot countries, including Kenya. $339 million dollars were earmarked for subsidies and 155.8 million doses were delivered in the first 18 months of the program (ICF International, 2012). Prices of subsidized ACTs in most pilot countries dropped below that of cheaper, ineffective drugs and substantial cost savings were seen by the end consumer. In Kenya, the retail market share of ACTs increased from 12% to 61% in the first 18 months of the program (Tougher et al., 2012). However, there is concern that dramatically lowering the price of ACTs opened the door to over-treatment and overuse of ACTs.

As the price of drug declines, the demand for it increases amongst both appropriate and inappropriate users. Between 36-77% of fevers treated in the retail sector do not have malaria (Mangham, et al., 2012; Cohen et al., 2012). A study in Tanzania showed that 80% of ACTs are sold to patients without parasitemia and only 69% of parasitemic patients purchased an ACT (Briggs, et al., 2012). This mismatch between who needs an ACT and who purchases one highlights the importance of improving targeting of antimalarials purchased in the retail sector.

Very few of the millions of cases treated in the retail sector have a diagnostic test before treatment, including clients who purchased one of the 14.4 million courses of AMFm ACTs that were delivered to Kenya in 2011. It has been argued that RDTs could effectively be deployed in the retail sector. Experimental evidence from a study in western Kenya shows high uptake of free or heavily subsidized RDTs, exceptional adherence to the results of a positive test (>98% purchasing a

subsidized ACT), but low adherence to the results of a negative test. Sixty-three percent of clients with a negative RDT result purchased an ACT (Cohen, et al., 2012).

In 2012, the Global Fund revised the AMFm strategy and ended the stand-alone subsidy. Countries receiving Global Fund support for malaria control can incorporate wholesale drug subsidies into their malaria control portfolios, but within their existing budgets. In addition, they can now use Global Fund money to provide subsidized malaria rapid diagnostic tests (RDTs) to the private sector. Ideally, incorporating diagnosis into a subsidy program would allow subsidized ACTs to be targeted to those with confirmed malaria infection, thus reducing unnecessary overuse of ACTs and reducing the cost of the subsidy program per patient treated. However, there is little evidence to guide policy makers in the implementation of diagnostic testing within a subsidy framework in the retail sector.

In 2014, this team implemented a pilot study to test the use of a voucher to improve uptake of testing. We also included testing for free compared to charging a small amount of money for the test. Although results of that pilot are still being analyzed, we have shown that 1) uptake of testing is improved when offered through CHVs, 2) far fewer clients chose to be tested when asked to pay for testing and 3) clients with vouchers were charged a higher base price for drugs. This information has informed our study design. Specifically, we will not charge for RDTs and we will offer a voucher for a fixed reduced price rather than a discount.

This study will evaluate the public health impact of targeted antimalarials subsidies through scale-up by determining the community-wide effects of targeting an antimalarial subsidy through a partnership between CHVs in the public sector and the private retail sector. In the intervention arm, CHVs will offer household members free RDTs and a voucher allowing the purchase of a qualified ACT at a reduced fixed price in the retail sector conditional on a positive test while individuals in the comparison arm will only receive standard community health volunteer (CHV) visits. Cross-sectional household surveying at pre-intervention, and 6 months, 12 months, and 18 months post-baseline will allow us to determine any change in the percent of fevers that are tested for malaria and the effect of testing on subsequent drug purchasing decisions.

The primary hypothesis to be tested is that offering a fixed-price voucher that reduces the cost for ACT purchase in the retail sector conditional on a positive malaria test (targeted subsidy) can improve uptake of testing for malaria and will increase the proportion of fevers tested for malaria before treatment.

2.2 Scientific Rationale

There are compelling medical and public health reasons to reduce unnecessary consumption of antimalarials and strong evidence to support the use of RDTs in malaria case management. However, the large numbers of fevers that are treated in the informal health sector go undiagnosed. Consumption of first-line antimalarials has increased due to availability of subsidized ACTs in the retail sector. Inappropriate consumption is a drain on public funds and jeopardizes the useful therapeutic life of ACTs.

Community diagnosis of malaria using RDTs by CHVs has been done in several settings in Africa. Evidence shows that CHVs can safely and correctly use RDTs with appropriate training and supervision (Briggs et al., 2012; Chanda et al. 2011; Cohen et al., 2012; Hamer et al., 2012. This approach has now been adopted by Kenya's Malaria Control Unit as part of their National Malaria Control Plan.

Using a cluster randomized trial, we will describe the effect of a conditional ACT subsidy for only clients with a positive malaria test on the proportion of fevers tested for malaria and consequent drug purchasing decisions after testing.

2.3 Potential Risks and Benefits

2.3.1 Potential Risks

It should be noted that the RDTs used in this study are the same brand and test as those used by the Government of Kenya in public health facilities and in their community-based case management for malaria. The Malaria Control Unit has embraced the strategy of community-based diagnosis for malaria using RDTs and has begun to train CHVs in some areas, although the program remains small due to funding constraints. Neither the RDT itself, nor the use of the RDT by CHVs are experimental in this study. The RDT itself is not the point at which the subject is participating in research.

The intervention proposed is the use of a conditional voucher for a positive test. This voucher is offered to patients with a positive test, but the client is free to choose whether or not to use it. Participating in this study involves agreeing to receive or not a voucher depending on the test results and allowing us to record information about treatment seeking behavior following a test. There is a small risk of breach of confidentiality of these information.

While unlikely, there is a small potential for excess bleeding or infection associated with finger pricks conducted in the course of administering an RDT. Proper training of the CHVs according to strict protocols will further minimize these risks. Study participants will be advised to contact the CHV in the case of any adverse events which may occur after the visit; CHVs will be trained in the recognition of and response to the unlikely occasion of any adverse events. These risks are equivalent to the risk of seeking the same test from a facility or CHV outside of the study.

2.3.2 Known Potential Benefits

There is significant benefit to the client to know their malaria infection status prior to purchasing a drug. There is also a benefit to the client to be able to purchase an effective drug at a reduced, fixed price when they have a confirmed malaria infection, which may also reduce the likelihood that they would purchase an inappropriate or outdated therapy.

More broadly, there are important future benefits to rigorous testing of subsidy schemes that promote testing before treatment. This work will contribute to evidence-based policy making, improved access to malaria diagnosis and ultimately reduced potential for spread of antimalarial resistance.

3 OBJECTIVES

The overall objective of this study is to evaluate the public health impact of targeted antimalarial subsidies through scale-up by determining the community-wide effects of targeting an antimalarial subsidy through a partnership between CHVs and the private retail sector. We will use a cluster-randomized design using established community health volunteers (CHVs) in both areas. The conditional subsidy will be offered in the form of a voucher providing for the purchase of a WHO-qualified ACT at a reduced, fixed price to those with a positive malaria test that can be redeemed at a local drug retailer.

The **primary outcome** of this study is to compare the percent of fevers that receive a malaria test from any source between the intervention and control arms.

The **secondary outcomes** of this study will also be measured and compared between intervention and control arms. The main secondary outcome is the percent of all ACTs used that were taken by people with a malaria positive test. Additional secondary outcomes are: the percent of all ACTs used that were taken by people without a test, the percent of those with a positive test who got an ACT, and the percent of those with a negative test who got an ACT.

4 STUDY DESIGN

We will use a cluster-randomized design to assign community units to either an intervention or control arm. The study will be carried out in two sub-counties in Kenya with similar malaria burden but different access to health services. Community Units (CUs) in each sub-county are the clusters to be randomized. A community unit consists of 1000 households (approximately 5000 people) 10 CHVs and One Community Health Extension Worker (CHEW). Each CHV is responsible for 100 households while the CHEW supervises the 10 CHVs. There are 32 CUs in total across both sub-counties, 20 in Bungoma East (10 in Ndivisi and 8 in Bokoli) and 14 in Kiminini. Four of the CUs in Bungoma East and 4 of the CUs in Kiminini have health facilities. To ensure that the randomization is balanced with regard to the presence of health facilities, we use this as a strata in our randomization. Half of the community units in each study area within each strata (9 in Bungoma East sub-county and 7 in Kiminini) will be randomly allocated to the intervention and the remainder of the community units to the comparison arm (refer to Table 1). We will screen 2884 subjects for eligibility in each arm at each of 4 outcome assessment surveys (refer to Section 7.2 for calculation of sample size, refer to Section 6.1 for study procedures, including data collection) and we expect to enroll 640 assuming a fever prevalence of 22.21% (see Section 7.2 for calculation of expected prevalence) of households have had an individual with fever in the last one month. We expect minimal contamination between the intervention and the control arm since CHVs are organized by geographic location and are responsible for specific households.

Table 1

	**Health Facility?**	**Number of Community Units (CUs)**				**Number of subjects with fever in each of the 4 cross-sectional surveys[1]**	**Number of subjects screened in each of the 4 cross-sectional surveys[2]**
**Group**		Bungoma East		Kiminini	Row Total		
		Ndivisi	Bokoli				
**Intervention arm**	YES	2	0	2	4	160	721
	NO	3	4	5	12	480	2162
**Comparison arm**	YES	2	0	2	4	160	721
	NO	3	4	5	12	480	2162
**Total number**		10	8	14	32	1280	5766

[1]Number of CUs x 40 individuals per CU

[2]Number of subjects with fever / 22.21% prevalence

CHVs will be trained to use RDTs to diagnose malaria in household members with documented or reported fever. Households in intervention CUs will be informed of the intervention and encouraged to contact the CHV for any febrile illness in the home. Household members with a negative RDT will be given a referral note documenting the results of the RDT and the date it was performed in the event they choose to go to a health facility to seek diagnosis and treatment for a different cause.

Intervention: Households with a positive RDT will be given a serialized voucher. The voucher will entitle the holder to purchase a quality assured ACT in the retail sector at a reduced, fixed price. The holder will redeem the voucher at any participating retailer by presenting both the voucher and the positive RDT provided by the CHV in a sealed plastic pouch.

All retail shops that serve customers in the intervention clusters will be identified through a comprehensive census. Shop owners and shop attendants will be invited to participate in the intervention. When a community member presents a voucher for redemption, participating outlets will collect both the voucher as well as the used positive RDT. Both will be given to the study team in return for payment on the value of the voucher.

The CHV will attempt to follow up on all clients receiving an RDT 4 days after testing to record what action was taken and what treatment, if any, was obtained. The CHV will provide further advice or referral depending on persisting symptoms.

Comparison Arm: Community health volunteers will conduct standard health education and health promotion activities. ACTs are available in the retail sector at unsubsidized and government subsidized prices.

Study outcomes and additional data will be collected via population-based cross-sectional household surveys in all of the 32 CUs at four regularly spaced time points: baseline, 6 months, 12 months, and 18 months post-baseline. We will use independent survey teams blinded to the assignment of arms to collect household data in a random sample of homes. The households will be selected randomly using a systematic sampling approach and selection will be independent of whether they received service from a CHV. Only one fever case from each household will be included. Data obtained from these population-based surveys will be used to evaluate the impact of the community intervention.

The overall intervention strategy is summarized in Figure 1 below.

The primary outcome of this study is to compare the percent of fevers that receive a malaria test from any source between the intervention and control arms. Testing is the gateway to targeting and if we cannot improve the proportion tested, then the voucher subsidy program will not have any effect. The main secondary outcome is the percent of all ACTs used that were taken by people with a malaria positive test. Additional secondary outcomes are: the percent of all ACTs used that were taken by people without a test, the percent of those with a positive test who got an ACT, and the percent of those with a negative test who got an ACT. Completion of the treatment course of ACT may be influenced by the cost of the drug; therefore we will also compare drug adherence amongst

those who redeemed a voucher for their ACT and those who paid the retail price. See Table 2 for detailed definitions of study outcomes.

Table 2. Study Outcomes Derived from Survey Questions

Outcomes	Survey questions used
**Primary Outcome Measures:**	
1. Comparison of percent of fevers that receive a malaria test from any source between arms.	*45. Did you have a malaria test? (1="Yes")*
**Secondary Outcome Measures:**	
1. Percent of all ACTs used that were taken by people with a malaria positive test.	*44. Did you have a malaria test? (1="Yes")*<br>*50. What were the results of the malaria test? (1="Positive")*<br>*60. Which medicines did you take? (1="Coartem/Artefan/other AL")*<br>*65. Did you start taking the Antimalarial before or after the malaria test? (2="After")*
2. Percent of all ACTs used that were taken by people without a test	*45. Did you have a malaria test? (2="No")*<br>*60. Which medicines did you take? (1="Coartem/Artefan/other AL")*
3. Percent that take AL if positive	*44. Did you have a malaria test? (1="Yes")*<br>*50. What were the results of the malaria test? (1="Positive")*<br>*60. Which medicines did you take? (1="Coartem/Artefan/other AL")*<br>*65. Did you start taking the Antimalarial before or after the malaria test? (2="After")*
4. Percent that take AL if negative	*44. Did you have a malaria test? (1="Yes")*<br>*50. What were the results of the malaria test? (2="Negative")*<br>*60. Which medicines did you take? (1="Coartem/Artefan/other AL")*<br>*65. Did you start taking the Antimalarial before or after the malaria test? (2="After")*
**Other Outcome Measures:**	
1. Percent of people that took ACTs and received a correct dose*	*64. If AL, how many pills were given? If not AL, skip to 64*<br>*1=6*<br>*2=12*<br>*3=18*<br>*4=24*<br>*5=Don't know/remember*<br>*6=OTHER: _____________*<br>*66. If AL, did you / patient complete the full course?*<br>***1=Yes***<br>*2=No*<br>*3=Not sure*<br>*4=Don't remember*<br>*68. If AL, how many days to complete the course?*

Figure 1: Diagram of intervention strategy

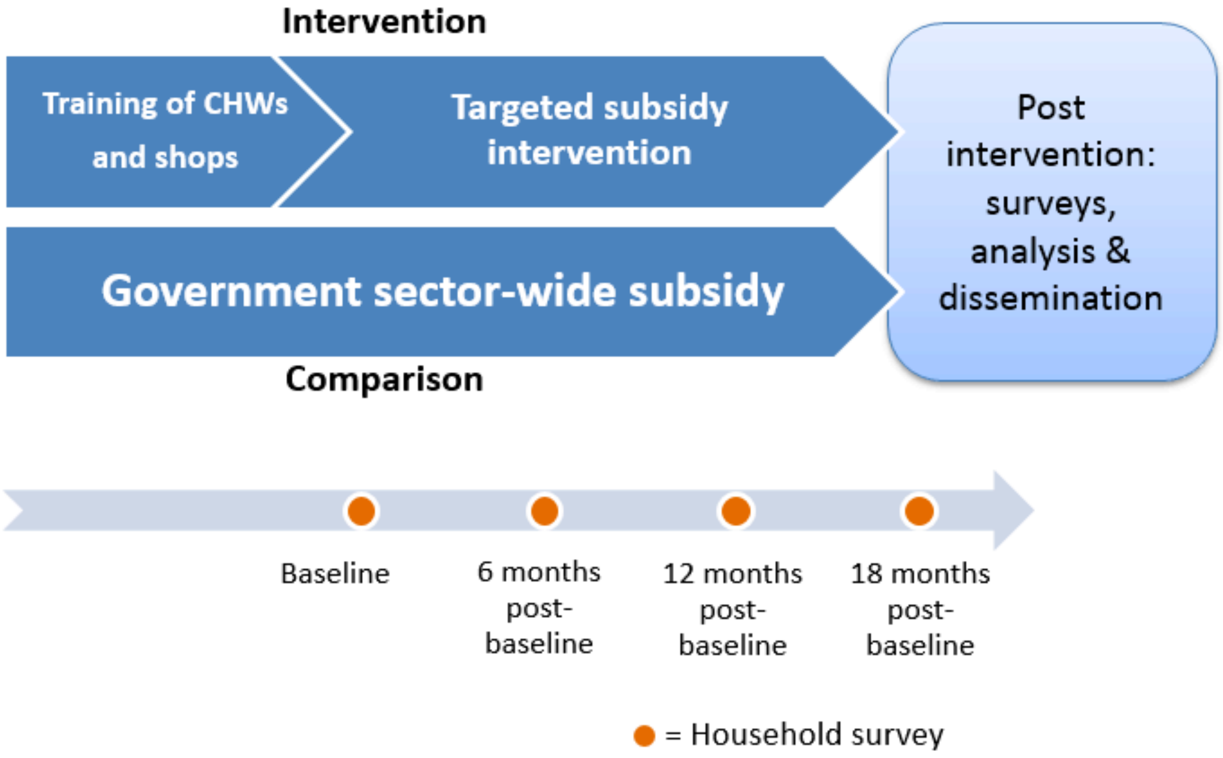
Intervention
Training of CHWs and shops
Targeted subsidy intervention
Government sector-wide subsidy
Comparison
Post intervention: surveys, analysis & dissemination
Baseline
6 months post-baseline
12 months post-baseline
18 months post-baseline
= Household survey

5 Study Population

This study will take place in Bungoma East and Kiminini sub-County. Both are high malaria transmission areas. The study population will be all individuals resident in the 20 community units in Bungoma East sub-county and 12 community units in Kiminini.

5.1 Selection of the Study Population

Half of the community units in each study area (10 in Bungoma East and 6 in Kiminini) will be randomly selected to be included in the intervention arm. The remainder of the community units will be the comparison arm. CHVs are responsible for specific households and they know the household members in their allotted households.

5.2 Inclusion/Exclusion Criteria

INCLUSION CRITERIA

Subjects who meet all of the following inclusion criteria will be eligible to participate in the study

Intervention participation criteria:

- Client is older than 1 year
- Client has fever or history of fever or feeling unwell with a malaria-like illness within the last 2 days
- Client or their parent/legal guardian (if under 18) consents to participate

Cross sectional survey participation criteria:

- Household representative in the intervention or control arm
- At least one member in the respondent's household with a history of fever or feeling unwell with a malaria like illness within the last four weeks
- Respondent is older than 18 years

EXCLUSION CRITERIA

Intervention exclusion criteria:

- Client has signs of severe disease or other problem requiring immediate referral to a health facility
- Client has already visited a health facility, taken or purchased antimalarials for the current illness.

Cross sectional survey exclusion criteria:

- Households not in the intervention or control arms

6 STUDY PROCEDURES/EVALUATIONS

6.1 Study Procedures

CHVs in the intervention arm will be trained to use RDTs to diagnose malaria in household members with documented or reported fever. The RDT that the study will use is the CareStart™ Malaria HRP-2 Pf Test. HRP2 malaria rapid diagnostic tests have been shown to have an average sensitivity and specificity of 94.8% and 95.2%, respectively, for *P. falciparum* infection when compared to light microscopy (Abba et al., 2011). This particular brand of test was reported to have sensitivity >90% even for low density infections and a false positive rate of <1% (World Health Organization, 2014). Several studies have demonstrated that CHVs can safely and correctly use rapid diagnostic tests (Briggs et al., 2012; Chanda et al. 2011; Cohen et al., 2012; Hamer et al., 2012). Although community-based diagnosis has been formally adopted by the Kenya Malaria Control Unit, roll-out has been limited due to funding constraints. Therefore we will have an opportunity to help implement this policy in the intervention areas and will train the CHVs in comparison areas at the conclusion of the study.

CHVs will be trained using a validated 3-day curriculum (based on the Kenya Ministry of Health curriculum) in conjunction with practical, skills-oriented sessions. Training session facilitators will include members of the study team, CHEWs, members of the sub-county health management team (SC-HMT), and peer mentors with extensive experience administering RDTs during previous studies. During the intervention, CHVs will be continuously monitored by both the study team and the CHEWs to ensure proper use and interpretation of RDT results.

Households in the 16 intervention CUs will be informed of the intervention and encouraged that any household member who experiences a febrile illness should visit the CHV in his/her village. The specific place where the CHV can be found may already be common knowledge within the community, but will also be made clear during community sensitization efforts. A mobile number at which the CHV can be reached will also be provided to the community; the CHV will attempt to make arrangements to visit sick clients who contact the CHV but who face difficulties in reaching the CHV's established location. For participants presenting for evaluation, the project CHV will assess and record on a standardized encounter form basic medical and medications history. Data about febrile household members and their RDT results will be collected by CHVs using customized carbonless-copy client registers designed to be read and digitized by Captricity (Captricity, Inc.) for automated data entry. Data will be routinely scanned and digitized by field supervisors. The CHV will take the participant's temperature and evaluate participant-reported fever history in the past two days. The CHV will record

participant-reported information on any medications taken for the treatment of malaria in the past two weeks. The CHV will assess whether women of child-bearing age presenting for evaluation may be pregnant based on self-reported date of last menstrual period. A participant having a measured temperature above 37.5 C or self-reporting a fever in the past two days will be administered an RDT (at no cost); the results will be recorded and provided to the participant.

A participant with a negative RDT will be issued a referral note documenting the results of the RDT and the date it was performed in the event they should choose to go to the health facility. This would eliminate the need to be re-tested at the facility. Children under 5 with signs of pneumonia (fast breathing, cough) or any individual with danger signs will be referred to a facility regardless of the results of the malaria test.

A participant having a positive RDT will be given a serialized voucher for a quality-assured ACT purchased at a participating drug shop. The voucher will entitle the holder to purchase a quality-assured ACT at a reduced, fixed price in the retail sector (Table 3). The fixed price will vary by age group according to the dosage required for the patient; the fixed price for a full course of treatment will be 10 KES for a child under three years of age (6 tablets), 15 KES for a child aged 3-8 years old (12 tablets), 20 KES for an adolescent aged 9-15 years old (18 tablets), and 40 KES for an adult (aged 16 years or older, 24 tablets). The voucher will be valid for 3 days from the date of issue. The holder will redeem the voucher at any participating retailer by presenting both the voucher and the positive RDT (provided by the CHV in a sealed plastic pouch). The retailer will be reimbursed the difference between the normal retail price and the study price plus 5 shillings. Women with a positive RDT who the CHV determines may be pregnant will not be offered a voucher, but will instead receive a referral.

Table 3: Pricing scheme for voucher holders compared to standard retail prices.

Age group	Average unsubsidized price	Study-subsidized price for voucher holders
Adult dose (>16 years)	100-120 KES	40 KES
9-15 years	80 KES	20 KES
3-8 years	50 KES	15 KES

<3 years	40 KES	10 KES

All retail medicine outlets that serve customers in the intervention clusters will be identified through a comprehensive census. We estimate a total of 40-50 shops will be enrolled in total across both sites. Shop owners and shop attendants will be invited to participate in the intervention and will attend a one-day training on current Government of Kenya malaria treatment guidelines, the role of RDTs in case management, and the study procedures. The training of shop keepers/owners will also include identification of danger signs and procedures for referral when necessary. They will be encouraged to purchase ACTs at the normal government-subsidized price and will be provided with a list of wholesalers in order to participate in the voucher scheme. When a community member presents a voucher for redemption, participating outlets will collect both the voucher as well as the used positive RDT (stored in a sealed plastic pouch). Both will be given to the study team in return for payment on the value of the voucher. Saving and re-reading the used RDT will ensure that exactly one voucher is redeemed for each positive RDT. Patients who redeem vouchers will be logged in a register and a unique study number will be written on the RDT.

The CHV will attempt to follow up on all clients receiving an RDT 4 days after testing to record what action was taken and what treatment, if any, was obtained. The CHV will provide further advice or referral depending on persisting symptoms or danger signs. They may provide education on adherence to full course of medication.

In the comparison areas, CHVs will provide standard care including home-based health education and referral services. ACTs will be available in the retail sector at unsubsidized and government-subsidized prices. No free RDTs or ACT vouchers will be offered by the CHVs in the comparison area clusters during the intervention period.

Survey teams who are blinded to the assignment of arms and independent of the intervention will collect household data in four regularly spaced cross-sectional surveys (baseline, 6 months, 12 months, and 18 months post-baseline). Household survey data will be collected in a random sample of households through face-to-face interviews. The households will be selected randomly according to village rosters and selection will be independent of whether members of that household received service from a CHV. The sample of homes will be unique in each survey round. The surveys will ask household representatives whether members experienced any fevers in the preceding four weeks. For one reported fever per household, the survey team will record whether any drug was taken, which drug or drugs were taken, and where they were obtained. They will also ask the individual or caregiver whether any diagnostic test for malaria (RDT or microscopy

using blood from a finger-prick) was done prior to treatment and the results of the test (self-reported). Whenever possible, health records from a facility or CHV will be reviewed for test results and the drug packaging will be observed to reduce recall bias. Survey teams will also have examples of antimalarial drug packaging to help with identification of type and brand used. The household survey data will be collected on android netbooks running the OpenDataKit platform. Internal consistency checks and data quality checks will be programmed into the forms. Data will be uploaded onto a secure laptop after each day of data collection and reviewed by the data managers.

Monitoring and supervision activities will be ongoing throughout the intervention to ensure compliance and data quality. The study team will randomly visit participating retail outlets several times each month. The availability of ACTs and prices of antimalarials will be monitored during supervisory visits. The team will check the list of patients who redeemed vouchers and cross-check with the used positive RDTs. Monitoring of the implementation of the intervention will occur through routine program records kept by both CHVs and retail medicine outlets in the intervention arm. Supervisors will also conduct random visits to observe a test being done to ensure adherence to proper procedures and study protocol. In addition, monitoring for adherence to the intervention will occur through collection and re-reading of used RDTs, and unannounced supervisory visits to retail outlets. At the conclusion of the study, all shop owners and shop attendants from participating outlets will be invited to participate in a focus group discussion to give their views about participating in the intervention (Focus group discussion guide can be found in Appendix 29 March 1). This will help us understand the perceived value, advantages, and disadvantages of the voucher program from the perspective of the shops. We anticipate conducting 3 focus groups with 10-12 participants per group.

In addition, the performance and satisfaction of CHVs participating in implementation of the study will be evaluated through standardized observation and questionnaire. A randomly-selected sample of 90 CHVs are expected to complete both evaluation activities. First, CHVs will be surveyed via a structured questionnaire regarding their understanding of, and satisfaction with, their role in the study as well as information regarding their demographics and experience as a CHV. Then, in order to monitor the quality of the rapid diagnostic test procedure performed by CHVs, trained observers will use a standardized checklist and note which, if any, steps are misconducted or omitted as they observe the CHVs perform RDTs. The CHVs will also be asked to interpret about 10 used RDT cassettes. For this specific component, clients receiving RDTs for CHV evaluation will be recruited from amongst patients presenting to local health facilities for a malaria test (i.e., that would be receiving a malaria test anyway). Clients will be invited to receive a test from a CHV participating in this study, but then sent back to the referring clinician for treatment after the test has been performed. Note that if the RDT is positive, the client will not receive a voucher for an ACT (as is the case for standard clients receiving a CHV-administered RDT outside the context of this observation activity). Rather, clients receiving an RDT in the context of this observation activity would receive

the treatment determined by a health facility clinician, who would be informed of the results of the RDT. ACTs are free at the health facility. Encounter forms will not be completed for these clients. Satisfaction and motivation of CHVs in both comparison and intervention areas will also be measured at the conclusion of the study. 130 CHVs in each area (total of 260) will be interviewed to understand what aspects of their community work contribute to their motivation and retention and whether that differs between intervention and comparison areas (Appendix 29March 2)

6.2 Laboratory Evaluations

6.2.1 Laboratory Evaluations/Assays

Evaluation of samples is limited to RDTs performed and evaluated in the field. No evaluations will take place in the laboratory.

6.2.2 Specimen Collection, Preparation, Handling and Shipping

Positive RDTs will be provided to the client in a sealed plastic bag, to be presented to the drug shop when redeeming a voucher. Supervisors will retrieve these tests from the drug shop regularly for diagnosis confirmation.

6.2.2.1 Instructions for Specimen Preparation, Handling, and Storage

Collection of samples will be limited to the extent described above. All RDTs (after supervisor confirmation) and associated waste will be disposed of properly according to strict protocol.

6.2.2.2 Specimen Shipment

NA

7 STATISTICAL CONSIDERATIONS

7.1 Study Outcome Measures

All study outcomes will be those measured in the four regularly spaced population-based cross-sectional surveys (baseline, 6 months, 12 months, and 18 months post-baseline). The primary outcome will be the percent of fevers that are tested for malaria either in the community or in the facility. Testing is the gateway to targeting and if we cannot improve the proportion tested, then the voucher subsidy program will not have any effect. The secondary outcomes of this study are to measure and compare between intervention and control arms. The main secondary outcome is the percent of all ACTs used that were taken by people with a malaria positive test. Additional secondary outcomes are: the percent of all ACTs used that were taken by people without a test, the percent of those with a positive test who got an ACT, and the percent of those with a negative test who got an ACT. Completion of the treatment course of ACT may be influenced by the cost of the drug; therefore we will also compare drug adherence amongst those who redeemed a voucher for their ACT and those who paid the retail price. All endpoints will depend on self-report supplemented by any medical records that describe diagnosis and treatment of the eligible fever episode and inspection of medicine packaging of drugs taken for the illness, if available. We will evaluate our study endpoints across all individuals >1 year of age. Although studies often focus on the treatment of fevers in children, the majority of ACT overuse occurs in adults. Adults are less likely to have a febrile illness caused by malaria, less likely to have a diagnostic test before treatment, and more likely to receive treatment in the informal retail sector. Therefore, increasing testing and reducing unnecessary use amongst adults has the largest potential for improving targeting.

To help clarify the population to which our outcomes apply, we show in Figure 2 the expected participant flow in the intervention and comparison arms.

Figure 2: Participant Flow and assumed probabilities.

Note that percentages on the branches of the tree are probabilities conditional on the previous step. Percentages at the end of the branch represent overall percentages out of 100% for each arm.

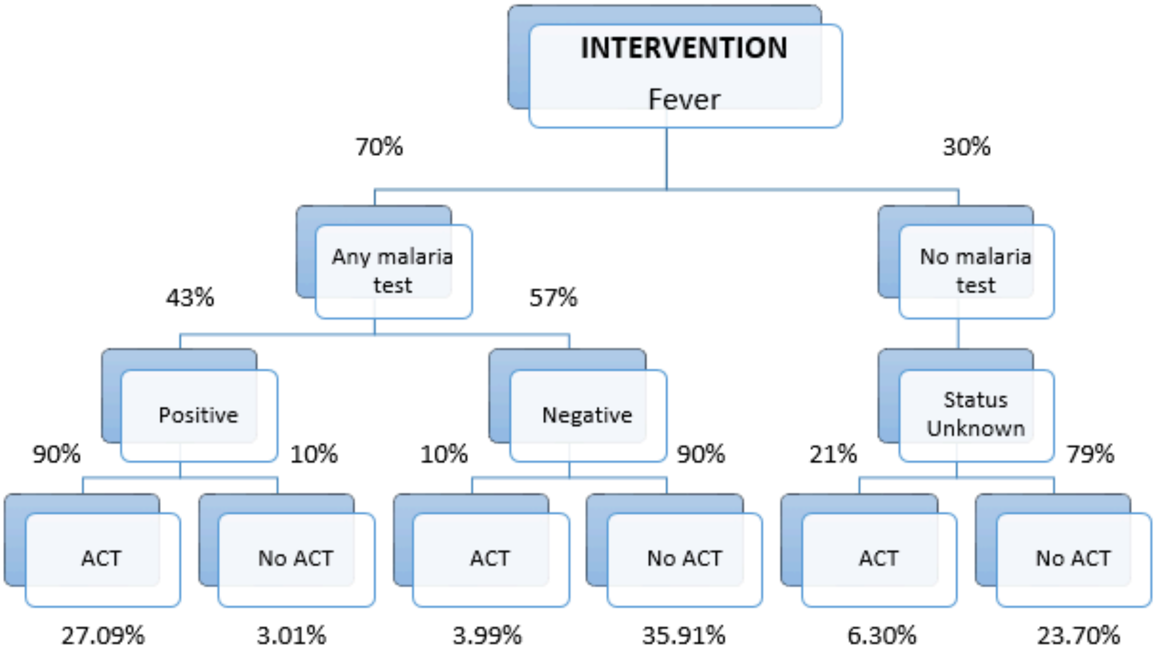

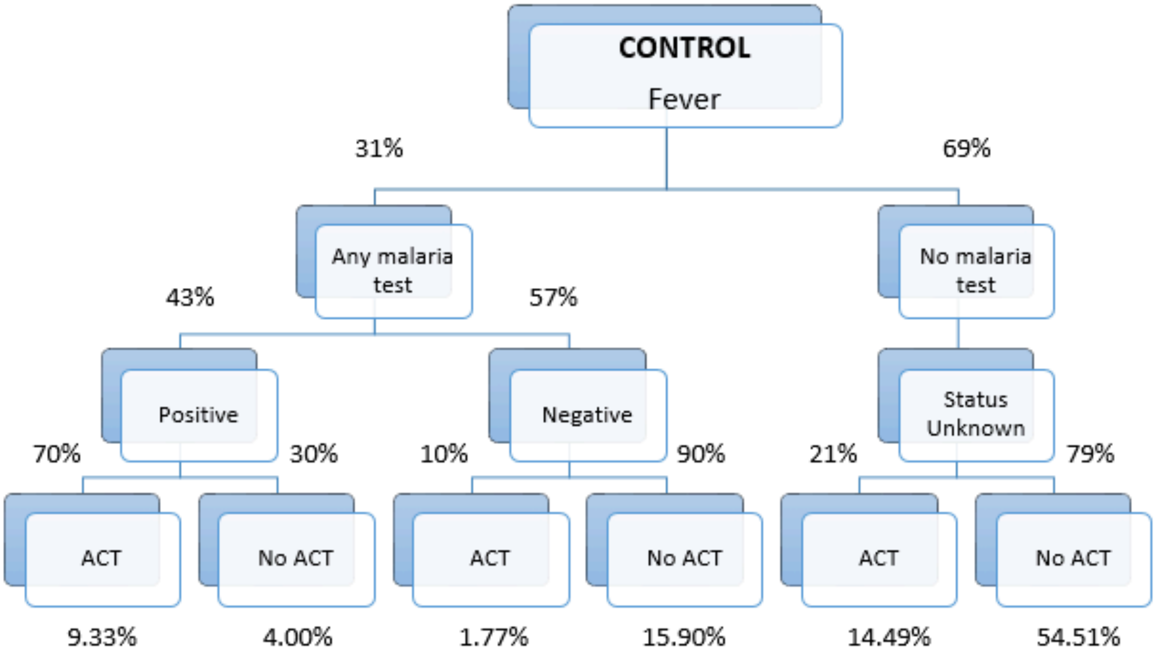

7.2 Sample Size Considerations

The study will survey 40 people with fever in the previous 4 weeks in each CU at each of the 4 measurement time points (baseline, 6, 12 and 18 months post-baseline). With 16 CU per arm, this will total 640 fevers per arm at each time point for a total of 1280 across both arms. We anticipate that approximately 22F% of households will have a member with a self-reported fever within the previous 4 weeks, so that the study team outcome assessors will need to visit 2884 households per arm at each measurement time point. All endpoints will be evaluated at 6 months post-baseline to determine whether high coverage of testing can be scaled-up quickly. Evaluation of the endpoint at 12 and 18 months will measure the saturation level of the intervention.

Based on pilot data from Bungoma, we anticipate that the proportion of fevers in the past 4 weeks who underwent testing for malaria (i.e. our primary outcome) will be 70% vs. 31% in intervention vs. control arms. These assumptions together with other assumptions are shown in Figure 2. Specifically, pilot data suggests that 43% of tested fevers will be positive (with no difference between arms). Of those with a positive test, 90% vs. 70% will purchase ACT in the intervention arm vs. control arm. Only 10% of those with a negative test are assumed to purchase ACTs (at the retail price) in both arms. Of those without a test, pilot data indicates that 21% purchase an ACT, and we expect this to be the same in both arms. Based on these assumptions, Table 4 shows the expected proportions for each of our primary and secondary outcomes and the number of fevers per CU which will be included in the denominator of each outcome (since all our secondary outcomes are conditional on testing or on ACT use so that the denominators are smaller than our assumed 40 fevers per CU). The table also reports the calculated interclass correlation coefficient and anticipated power of the comparisons. We base the main calculation (titled "Original" in Table 4) on the results of our preliminary pilot study, then expand into various scenarios that may occur during our proposed full-scale cRCT to demonstrate how power may change under various conditions. Details of the rationale and other assumptions of power calculations are listed below.

We calculated power based on a cluster randomized two-sample two-tailed t-test for the comparison of two proportions using standard formulae, which use the coefficient of variation as the measure of between-cluster variability (Hayes & Moulton, 2009). Specifically, we calculated power for the two-tailed comparison of the intervention group vs. the control group for the primary outcome of the proportion of clients with fever in the previous four weeks who reported being tested for malaria prior to any treatment. To ensure that our overall two-tailed Type I error (alpha) was 5%, we fixed the alpha level at 1.667% (i.e. 5%/3) for each of the 3 follow-up time points (6, 12 and 18 months post-baseline), using the conservative Bonferroni correction (Aickin & Gensler, 1996). In practice, we will use the less conservative Benjamini Hochberg procedure to adjust the actual alpha level for simultaneously testing the primary outcome at those 3 time-points (Benjamini & Yekutieli, 2001). To further protect against possible losses in power due to the stratified study design, we conservatively based the power on a matched-cluster design, as the stratified design is expected to have more power than a matched design.

Table 4: Summary of study outcomes under varying scenarios, assumed effects, ICC, n per cluster, and power at each of 3 follow-up time points for a cluster-randomized trial of 16 control CUs vs 16 intervention CUs at overall 5% type-1 error rate for each outcome (Bonferroni correction for 3 time points)

			**Scenario** (with differences from scenario 1 **highlighted)**		
	**Original**	**1. Calculated from pilot data**	**2. Like Scenario 1, except Positive (%) = 43% for intervention**	**3. "Best case": Like Scenario 2, except in intervention Positive (%) with ACT = No test (%) with ACT = 50%**	**4. "Worst case": Like Scenario 2, except in intervention positive (%) and take ACT 70%**
**Assumptions**	**Intervention vs. Control**				
Percent of fevers with test	70% vs. 31%	70% vs. 31%	70% vs. 31%	70% vs. 31%	70% vs. 31%
Percent of tested fevers that are positive	43% vs. 43%	**69%** vs. **69%**	**43%** vs. 69%	**43%** vs. 69%	**43%** vs. 69%
Percent that take ACT if positive	90% vs. 70%	90% vs. 90%	90% vs. 90%	90% vs. 90%	**70%** vs. 90%
Percent that take ACT if negative	10% vs. 10%	**70%** vs. **70%**	70% vs. 70%	**50%** vs. 70%	70% vs. 70%
Percent that take AL with no test	21% vs. 21%	**80%** vs. **80%**	80% vs. 80%	**50%** vs. 80%	80% vs. 80%
**Outcome**	**Intervention vs. Control**<br>**ICC; Assumed n per cluster (set at min of intervention and control); Power**				
Percent of fevers with test	70% vs. 31%<br>0.073; 40; 98%				
Percent of ACT taken by those who test positive	72% vs. 36% 0.027; 10; 90%	53% vs. 24%; 0.027; 32; 98%	34% vs. 24% 0.027; 32; 36%	44% vs. 24% 0.027; 25; 81%	29% vs. 24% 0.027; 29; 9%
Percent of ACT taken by those with no test	17% vs. 57% 0.064; 10; 100%	29% vs. 68%; 0.064; 32; >99%	30% vs. 68% 0.064; 32; >99%	24% vs. 68% 0.064; 25; >99%	33% vs. 68% 0.064; 29; >99%

Percent that take ACT after positive test	90% vs. 70%<br><br>0.074; 5; 50%	90% vs. 90%;<br><br>0.074; 9; -	90% vs. 90%<br><br>0.074; 9; -	90% vs. 90%<br><br>0.074; 9; -	70% vs. 90%<br>0.074; 9; 62%
Percent that take ACT after negative test	10% vs. 10%<br><br>0.007; 7; -	70% vs. 70%;<br><br>0.007; 4, -	70% vs. 70%<br><br>0.007; 4, -	50% vs. 70%<br><br>0.007; 4; 40%	70% vs. 70%<br>0.007; 4; -

* See Figure 2

The coefficient of variation (CV) for each outcome was estimated based on preliminary (unpublished) pilot data and substantive knowledge of the research setting. In the absence of measurements on any of the proposed outcomes, we used the following procedure as recommended in the literature (Hayes & Moulton, 2009). First, we took the assumed value of the outcome in the control group (see Table 4), assumed a normal distribution on the CU-specific proportions with the outcome centered around the control arm proportion. The SD of that distribution (i.e. the between-CU SD) was then calculated based on a plausible range for 95% of possible CU-specific proportions agreed by the study team. We used a conservative width of 0.5 for the percent of fevers that take a test, percent of ACT taken by those with no test, and percent that take ACT after a negative test; a width of 0.25 for percent of ACTs taken by those with a positive test, and a width of 0.10 for percent that take ACT after a positive test. Corresponding intra-cluster correlation coefficients (ICCs) were all estimated according to the formula ICC = $CV^2*\pi/(1-\pi)$, where π is the expected proportion for the outcome in the control group (Hayes & Moulton, 2009).

As noted above, with 40 fevers per cluster (1280 in total across all 32 CUs), we are very well powered for our primary outcome (under most scenarios), and for our secondary outcomes except for the two secondary outcomes related to the proportion buying ACT following a test result (either positive or negative). As we do not expect the intervention to influence the proportion who buy ACT following a negative result, we are not concerned that we are not powered for this outcome. Even though we do not expect this to be different between the arms, this is an important metric of appropriate malaria treatment and still important to evaluate in our study area.

In order to determine how many people will need to be screened in order to obtain the required sample size of 1280 fevers across all 32 study CUs, we use data collected in previous studies in the region. From our preliminary results, we know that the period prevalence of fevers in children under 5 in the last one month is 33% in the Bungoma East area and 38% in Kiminini. We expect the incidence of fever in older children and adults to be roughly half of that in children. Based on a population mix of 1:3 for under 5s vs. older children and adults we assume that the prevalence of fever in the previous four weeks is 22.21%. Therefore, overall we assume that 22.21% of the population in both arms will experience a fever during the previous four weeks. Consequently we will need to sample just over four-times as many clients, which corresponds to a total of 5766

individuals (2884 per arm) to be sampled at each of 3 follow-up surveys (6, 12 and 18 month post-baseline). Assuming comparable intervention effects at the final time point, we will use the same sample size at all 4 data collection time points. With a team of ten, surveys should take approximately 2 months to complete at each time point.

7.3 Participant Enrollment and Follow-Up

Eligibility for enrollment will be determined according to the specific inclusion and exclusion criteria outlined in Section 5.2 of this protocol. The CHV in the intervention arm will attempt to follow up on all clients receiving an RDT 4 days after testing to record what action was taken and what treatment, if any, was obtained.

7.4 Analysis Plan

All study outcome measures are individual-level binary outcome measures. Results will be presented as effect sizes with 95% confidence intervals. We will compare each of the study outcomes at each time point between study groups. We will account for the stratified design and clustering by CU for all analyses. All analyses will be based on the intention-to-treat principle.

Models with adjusted and unadjusted estimates will be presented for all analyses. Adjustment covariates will be pre-specified based on prior knowledge of relationships with the outcome variables. Adjustment covariates will include: age, sex, household size, and household socioeconomic status. Analyses may be adjusted for the baseline level of the outcome variable in order to improve the precision of the measurement of the treatment effect.

Baseline levels of demographic variables as well as outcome variables will be compared to assess balance of covariates between treatment arms. No formal hypothesis testing will be performed in these comparisons.

Autonomy of testing and treatment decisions depend on the age of the febrile individual. It is possible that the effects of the intervention may differ based on age (i.e. children vs. adults). We will examine this possibility by testing the presence of an interaction effect between age and treatment group. Differences between sub-groups will be identified by the significance of the interaction effect. If outcomes do indeed differ by subgroup, we will report results separately by subgroup.

Sub-analysis will also be performed to examine the sensitivity of results to the precise definition of the outcome. The first set of sub-analyses will look at the set of main outcomes using only those malaria tests for which documentation was provided to the data collectors (i.e. duplicate form for those who tested at the CHV center, health booklet for those who tested at facilities).

Our primary aim is to determine whether there is significant difference between the 2 study arms in the proportion of clients with fever who are tested prior to any treatment after adjusting for relevant covariates at each of the follow-up periods. As noted above in Section 7.2 (Sample Size Considerations), we will use the Benjamini Hochberg procedure for determining significance of the 3 tests of the difference between groups at each follow-up time point. We will also compare secondary measures using the same modeling and adjustment approach.

8 SUBJECT CONFIDENTIALITY

Data collected by CHVs about client visits will be entered into electronic databases using web-based technology and will not include names and birthdates of individuals. Client information from cross-sectional household surveys will be collected on encrypted, password-protected tablets. Data will be removed from the tablets weekly and stored in a secure study server. Participants will be assigned a study number which will link them to their study data. For all databases, all personal identifying information will be stored separately from the study data and will be linked only by the unique study number. Field supervisors may review data including identifying information to ensure compliance with study procedures. All data released to co-investigators or statisticians for analysis will be anonymized. Research assistants will be trained on proper data collection techniques and protection of client data.

All data released to co-investigators or statisticians for analysis will be anonymized. GPS coordinates of retail outlets and households is considered to be personal identifying information. Therefore, the data manager will use the coordinates to calculate distances between the household and the health facilities and only distance information will be released for analysis. If coordinates are required to make maps of the study area, a random error of 50-100 meters will be added to each coordinate to protect individual household identities before the data is released to the analyst. Recipients of study data will be asked to sign a data sharing agreement that specifies what the data may be used for (specific analyses), criteria for acknowledging the source of the data, and the conditions for publication. It will also stipulate that the recipient may not share the data with other investigators. Requests for data use must be made directly to the PI and not through third parties.

8.1 Future Use of Stored Specimens

NA (No samples will be stored).

9 INFORMED CONSENT PROCESS

Potential subjects presenting to a CHV for testing will be screened for eligibility in privacy. Those who meet the inclusion criteria will be asked to provide written consent before testing. A consent document will be read and explained to the participant. A copy will be provided if requested. The subject will sign the encounter form to indicate consent (Appendix 1). A copy of the encounter form will be released to the patient and a copy will be retained by the study.

Clients selected to receive an RDT from amongst health facility attendees under the CHV observation (evaluation) activity will provide verbal consent, as their participation will meet the following criteria for a waiver - this study presents no more than minimal risk to the participant, and does not include any procedure for which consent would normally be required outside of the research setting; and the only record linking the subject and the research would be the consent document.

Potential respondents to the baseline, interim, and final household questionnaires will be asked to provide verbal consent for participation. We are requesting a waiver of documentation of informed consent for the household surveys only. Clients will complete a questionnaire which meets the following criteria for a waiver - this study presents no more than minimal risk to the client and does not include any procedure for which consent would normally be required outside of the research setting. The consent document would be the only information linking the respondent to his or her study id.

Shop owners of participating retail medicine outlets in the study areas will be asked to provide written informed consent for participating in the intervention, including honoring client vouchers and collecting used RDTs. For shops that choose not to participate in the voucher scheme, we will seek verbal consent for the study to collect study-related data only such as stocking and sales of antimalarials. We are requesting a waiver of documentation of informed consent for the survey as this study presents no more than minimal risk and no personal identifying information will be collected about the shop attendant/owner.

CHVs will provide verbal consent for CHV evaluation activities (surveying and observation), which meet the following criteria for a waiver - this study presents no more than minimal risk to the participant, and does not include any procedure for which consent would normally be required outside of the research setting.

9.1 Informed Consent/Assent Process (in Case of a Minor or Others Unable to Consent for Themselves)

The assent process for minors (defined as those under 18 years of age) will be to obtain consent from the minor's parent or legal guardian for the participation of the minor and verbal assent from minors over the age of 8 years. Eight years is considered the age above which assent is required in Kenya. This applies only to subjects presenting to the CHV for testing. Minors will not be eligible to participate in the household survey.

10 LITERATURE REFERENCES

Abba K., Deeks J.J., Olliaro P.L., Naing C.M., Jackson S.M., Takwoingi Y., Donegan S., and P. Garner, *Rapid diagnostic tests for diagnosing uncomplicated P. falciparum malaria in endemic countries (Review).* Cochrane Database Syst Rev., 2011. (7): CD008122.

Abuya, T.O., W. Mutemi, B. Karisa, S.A. Ochola, G. Fegan, and V. Marsh, *Use of over-the-counter malaria medicines in children and adults in three districts in Kenya: implications for private medicine retailer interventions.* Malar J, 2007. **6**: p. 57.

Aickin, M., and Gensler, H., *Adjusting for multiple testing when reporting research results: the Bonferroni vs Holm methods*. Am J Public Health, 1996. (86): p. 726–728.

Amin, A.A., V. Marsh, A.M. Noor, S.A. Ochola, and R.W. Snow, *The use of formal and informal curative services in the management of paediatric fevers in four districts in Kenya.* Trop Med Int Health, 2003. **8**(12): p. 1143-52.

Benjamini Y., and Yekutieli D. *The control of the false discovery rate in multiple testing under dependency*. Annals of statistics. 2001:1165-88.

Briggs, M., A. Kalolella, K. Bruxvoort, G. Lopez, C. Festo, P. Lyaruu, M. Kenani, B. Johannes, S. Abdulla, C. Goodman, and S.P. Kachur, *Prevalence of malaria parasitemia and medication utilization patterns among clients seeking care for malaria or fever in drug shops in Tanzania, March – May 2012.*

Cameron, A.C., J.B. Gelbach, and D.L. Miller, *Bootstrap-Based Improvements for Inference with Clustered Errors.* Review of Economics and Statistics, 2008. **90**(3): p. 414-427

Chanda, P., B. Hamainza, H.B. Moonga, V. Chalwe, and F. Pagnoni, *Community case management of malaria using ACT and RDT in two districts in Zambia: achieving high adherence to test results using community health workers.* Malar J, 2011. **10**: p. 158.

Chuma, J., T. Abuya, D. Memusi, E. Juma, W. Akhwale, J. Ntwiga, A. Nyandigisi, G. Tetteh, R. Shretta,and A. Amin, *Reviewing the literature on access to prompt and effective malaria treatment in Kenya: Implications for meeting the Abuja targets.* Malar J, 2009. **8**: p. 243.

Cohen, J., P. Dupas, and S. Schaner, *Price Subsidies, Diagnostic Tests, and Targeting of Malaria Treatment: Evidence from a Randomized Controlled Trial.* National Bureau of Economic Research, 2012. **Working Paper 17943**.

Hamer, D.H., E.T. Brooks, K. Semrau, P. Pilingana, W.B. MacLeod, K. Siazeele, L.L. Sabin, D.M. Thea,and K. Yeboah-Antwi, *Quality and safety of integrated community case management*

of malaria using rapid diagnostic tests and pneumonia by community health workers. Pathog Glob Health, 2012. **106**(1): p. 32-9.

Hayes, R. J., and Moulton, L. H. (2009). *Cluster randomised trials.* Boca Raton, FL: CRC Press.

Hochberg, Y., and Tamhane, A.C. (2009). *Multiple comparison procedures*. New York, NY: John Wiley & Sons.
ICF International, *Independent Evaluation of the Affordable Medicines Facility - malaria (AMFm) Phase 1*, The Global Fund to Fight AIDS, Tuberculosis and Malaria 2012: Calverton, MD.

Littrell, M., H. Gatakaa, I. Evance, S. Poyer, J. Njogu, T. Solomon, E. Munroe, S. Chapman, C. Goodman, K. Hanson, C. Zinsou, L. Akulayi, J. Raharinjatovo, E. Arogundade, P. Buyungo, F. Mpasela, C.B. Adjibabi, J.A. Agbango, B.F. Ramarosandratana, B. Coker, D. Rubahika, B. Hamainza, T. Shewchuk, D. Chavasse, and K.A. O'Connell, *Monitoring fever treatment behaviour and equitable access to effective medicines in the context of initiatives to improve ACT access: baseline results and implications for programming in six African countries.* Malar J, 2011. **10**: p. 327.

Malik, E.M., K. Hanafi, S.H. Ali, E.S. Ahmed, and K.A. Mohamed, *Treatment-seeking behaviour for malaria in children under five years of age: implication for home management in rural areas with high seasonal transmission in Sudan.* Malar J, 2006. **5**: p. 60.

Mangham, L.J., B. Cundill, O.A. Achonduh, J.N. Ambebila, A.K. Lele, T.N. Metoh, S.N. Ndive, I.C. Ndong, R.L. Nguela, A.M. Nji, B. Orang-Ojong, V. Wiseman, J. Pamen-Ngako, and W.F. Mbacham, *Malaria prevalence and treatment of febrile patients at health facilities and medicine retailers in Cameroon.* Trop Med Int Health, 2012. **17**(3): p. 330-42.

Tougher, S., Y. Ye, J.H. Amuasi, I.A. Kourgueni, R. Thomson, C. Goodman, A.G. Mann, R. Ren, B.A. Willey, C.A. Adegoke, A. Amin, D. Ansong, K. Bruxvoort, D.A. Diallo, G. Diap, C. Festo, B. Johanes, E. Juma, A. Kalolella, O. Malam, B. Mberu, S. Ndiaye, S.B. Nguah, M. Seydou, M. Taylor, S.T. Rueda, M. Wamukoya, F. Arnold, and K. Hanson, *Effect of the Aff ordable Medicines Facility—malaria (AMFm) on the availability, price, and market share of quality-assured artemisinin-based combination therapies in seven countries: a before-and-after analysis of outlet survey data.* Lancet, 2012. **380**: p. 1916-1926.

Westgate, P.M. and T.M. Braun, *Improving small-sample inference in group randomized trials with binary outcomes.* Statistics in Medicine, 2011. **30**(3): p. 201-210.

World Health Organization, *World Malaria Report*. WHO, 2012: Geneva.

World Health Organization, *Malaria Rapid Diagnostic Test Performance: Summary results of WHO product testing of malaria RDTs: Round 1-5 (2008-2013)*. WHO, 2014: Geneva.
