# Supplementary material for: Improving rational use of ACTs through diagnosis-dependent subsidies: Evidence from a cluster-randomized controlled trial in western Kenya
Source: PLoS Med. 2018 Jul 17;15(7):e1002607. doi: 10.1371/journal.pmed.1002607 (PMC6049880; doi:10.1371/journal.pmed.1002607)
Supplement: S1 Table — (DOCX) [file pmed.1002607.s005.docx]

**S1 Table**. Weighted* characteristics of febrile participants and respondents (for participants < 18 years) by arm and follow-up survey time-point.

|  | **Baseline (N=2017)** | | **6-Months (N=1660)** | | **12-Months (N=1812)** | | **18-Months (N=1927)** | |
| --- | --- | --- | --- | --- | --- | --- | --- | --- |
| **Variable – Number (%), unless otherwise stated** | **Control** | **Intervention** | **Control** | **Intervention** | **Control** | **Intervention** | **Control** | **Intervention** |
| Age of febrile participant (years) |  |  |  |  |  |  |  |  |
| Under 5 | 221 (21.9%) | 239 (23.7%) | 210 (25.3%) | 201 (24.2%) | 183 (20.3%) | 207 (22.9%) | 171 (18.7%) | 191 (19.1%) |
| 5 to 17 | 388 (38.5%) | 405 (40.2%) | 319 (38.5%) | 325 (39.2%) | 367 (40.6%) | 359 (39.6%) | 416 (45.4%) | 463 (46.3%) |
| 18+ | 399 (39.6%) | 364 (36.1%) | 300 (36.2%) | 303 (36.5%) | 353 (39.1%) | 340 (37.5%) | 329 (35.9%) | 346 (34.6%) |
| Missing | 1 (.%) | 1 (.%) | 2 (.%) | 1 (.%) | 2 (.%) | 1 (.%) | 5 (.%) | 6 (.%) |
| Gender of febrile participant |  |  |  |  |  |  |  |  |
| Male | 379 (37.6%) | 416 (41.3%) | 323 (39.0%) | 312 (37.6%) | 356 (39.3%) | 357 (39.5%) | 369 (40.3%) | 423 (42.2%) |
| Female | 629 (62.4%) | 592 (58.7%) | 505 (61.0%) | 518 (62.4%) | 548 (60.7%) | 548 (60.5%) | 547 (59.7%) | 579 (57.8%) |
| Missing | 1 (.%) | 1 (.%) | 2 (.%) | 0 (.%) | 2 (.%) | 1 (.%) | 5 (.%) | 4 (.%) |
| Wealth Index (quintile) |  |  |  |  |  |  |  |  |
| 0 - 20th | 216 (21.7%) | 211 (21.1%) | 203 (25.2%) | 187 (23.5%) | 171 (19.6%) | 165 (19.0%) | 184 (20.3%) | 174 (17.4%) |
| >20 - 40th | 169 (17.0%) | 182 (18.2%) | 138 (17.1%) | 122 (15.3%) | 174 (20.0%) | 187 (21.5%) | 174 (19.2%) | 231 (23.1%) |
| >40 - 60th | 202 (20.3%) | 218 (21.8%) | 139 (17.3%) | 144 (18.1%) | 167 (19.2%) | 184 (21.1%) | 177 (19.5%) | 209 (20.9%) |
| >60 - 80th | 196 (19.7%) | 207 (20.8%) | 142 (17.6%) | 189 (23.7%) | 158 (18.2%) | 164 (18.8%) | 190 (20.9%) | 197 (19.7%) |
| >80th | 212 (21.3%) | 181 (18.1%) | 184 (22.8%) | 154 (19.3%) | 200 (23.0%) | 172 (19.7%) | 183 (20.2%) | 188 (18.8%) |
| Missing | 13 (.%) | 10 (.%) | 24 (.%) | 35 (.%) | 35 (.%) | 34 (.%) | 13 (.%) | 7 (.%) |
| Highest school level of patient/guardian |  |  |  |  |  |  |  |  |
| None or less than primary | 399 (39.9%) | 421 (42.0%) | 287 (34.6%) | 298 (36.1%) | 316 (35.0%) | 313 (34.6%) | 315 (34.4%) | 333 (33.3%) |
| Completed primary | 373 (37.2%) | 360 (35.9%) | 351 (42.4%) | 342 (41.4%) | 372 (41.2%) | 402 (44.4%) | 416 (45.4%) | 454 (45.4%) |
| Completed secondary | 230 (22.9%) | 221 (22.1%) | 190 (23.0%) | 186 (22.5%) | 214 (23.8%) | 190 (21.0%) | 185 (20.2%) | 213 (21.3%) |
| Missing | 7 (.%) | 6 (.%) | 2 (.%) | 4 (.%) | 3 (.%) | 2 (.%) | 5 (.%) | 6 (.%) |
| Distance – median (25^th^, 75^th^ percentile) |  |  |  |  |  |  |  |  |
| Nearest Retail medicine outlet (km) | 1.3 (0.7, 2.3) | 1.1 (0.7, 1.6) | 1.3 (0.7, 2.1) | 1.2 (0.7, 1.8) | 1.3 (0.8, 2.1) | 1.2 (0.8, 1.8) | 1.3 (0.8, 2.1) | 1.3 (0.8, 1.8) |
| Nearest Private Clinic (km) | 18.0 (9.8, 25.3) | 15.5 (2.9, 24.5) | 17.8 (10.3, 23.4) | 15.5 (7.9, 20.4) | 17.9 (10.2, 23.2) | 15.6 (8.1, 22.0) | 18.8 (10.9, 23.7) | 15.5 (8.2, 22.9) |
| Nearest Private Hospital (km) | 14.1 (8.5, 42.9) | 15.2 (9.8, 41.5) | 29.7 (11.6, 41.4) | 31.5 (10.5, 37.5) | 29.7 (11.7, 41.1) | 31.7 (10.6, 39.7) | 35.7 (13.4, 41.6) | 32.5 (10.9, 40.2) |
| Nearest Pubic Health Centre/Dispensary (km) | 2.2 (1.3, 3.2) | 2.4 (1.5, 3.5) | 2.4 (1.5, 3.3) | 2.4 (1.4, 3.6) | 2.4 (1.5, 3.3) | 2.4 (1.5, 3.6) | 2.5 (1.5, 3.4) | 2.5 (1.5, 3.7) |
| Nearest Public Hospital (km) | 5.9 (4.1, 8.2) | 4.1 (2.4, 8.3) | 7.3 (5.0, 14.2) | 8.2 (2.9, 12.2) | 7.4 (5.0, 14.3) | 8.2 (3.1, 12.2) | 7.8 (5.2, 14.7) | 8.2 (2.7, 12.4) |

* Note that observed totals (N) by time point are provided, whereas the table body contains weighted numbers. As a consequence, the sum of weighted numbers may slightly differ from the observed totals. All summaries are weighted using the following weight calculation: ${weight}_{ik}=\left( \frac{N_{k,total}}{32} \right)/{N_{ik}},$where i=1,…,32 indicates CU and k=0, 1, 2, 3 indicates baseline, 6-months, 12-months, and 18-months, respectively.
