# Supplementary material for: Improving rational use of ACTs through diagnosis-dependent subsidies: Evidence from a cluster-randomized controlled trial in western Kenya
Source: PLoS Med. 2018 Jul 17;15(7):e1002607. doi: 10.1371/journal.pmed.1002607 (PMC6049880; doi:10.1371/journal.pmed.1002607)
Supplement: S2 Table — (DOCX) [file pmed.1002607.s006.docx]

**S2 Table**. Coefficient of variation (CV) for all study outcomes measured at baseline and for cluster sizes at baseline.

| **Outcome** | **CV (%) of baseline outcomes^a^** | **CV (%) of baseline cluster sizes^b^** |
| --- | --- | --- |
| **Primary** |  |  |
| Took malaria test (AMONG ALL FEVERS) | 25.0 | 40.9 |
| **Secondary** |  |  |
| Targeted ACT use (AMONG ALL FEVERS) | 31.9 | 40.9 |
| Rational ACT use (AMONG ACT USERS) | 34.3 | 49.1 |
| Had no test (AMONG ACT USERS) | 28.1 | 49.1 |
| **Other** |  |  |
| Took ACT after a POSITIVE test | 13.5 | 52.3 |
| Took ACT after a NEGATIVE test | 92.1 | 74.6 |
| Took ACT with NO test | 26.8 | 48.5 |
| Test adherence among ALL tested | 11.5 | 48.2 |
| ^a^Based on baseline (N=32) CU-level proportions of outcome measures; ^b^Based on baseline (N=32) CU-level counts of individuals with the denominator for outcome measures. Therefore, the same CV of baseline cluster size is observed for outcomes with the same denominator e.g. for “took malaria test” and “targeted ACT use” which both have a denominator of “AMONG ALL FEVERS”. | | |
