# Supplementary material for: Improving rational use of ACTs through diagnosis-dependent subsidies: Evidence from a cluster-randomized controlled trial in western Kenya
Source: PLoS Med. 2018 Jul 17;15(7):e1002607. doi: 10.1371/journal.pmed.1002607 (PMC6049880; doi:10.1371/journal.pmed.1002607)
Supplement: S3 Table — (DOCX) [file pmed.1002607.s007.docx]

**S3 Table**. Weighted* design-adjusted and weighted design and covariate adjusted model-estimated between-arm differences^1^ comparing intervention and control arms in the primary outcome of uptake of testing, i.e. took a malaria test from any source among all recent fevers. This table provides complete regression output for the primary outcome presented in Table 3 of the main manuscript, including coefficients on all adjustment variables.

|  | **Risk Ratios** | | | | **Risk Difference** | | | |
| --- | --- | --- | --- | --- | --- | --- | --- | --- |
|  | **Unadjusted** | | **Adjusted** | | **Unadjusted** | | **Adjusted** | |
| **Variable** | **Estimate (95% CI)** | **P-value** | **Estimate  (95% CI)** | **P-value** | **Estimate  (95% CI)** | **P-value** | **Estimate  (95% CI)** | **P-value** |
| Intercept^2^ | 0.75 (0.57,0.98) | 0.0374 | 0.84 (0.66,1.06) | 0.1302 | 0.29  (0.15,0.42) | 0.0002 | 0.37  (0.25,0.49) | <.0001 |
| Intervention | 1.05 (0.91,1.23) | 0.4705 | 1.07 (0.93,1.24) | 0.3464 | 0.03  (-.05,0.10) | 0.4708 | 0.03  (-.04,0.10) | 0.3513 |
| 12-Months | 0.93 (0.82,1.04) | 0.1972 | 0.93 (0.83,1.04) | 0.2026 | -.03  (-.09,0.02) | 0.2012 | -.03  (-.08,0.02) | 0.2113 |
| 18-Months | 0.95 (0.82,1.11) | 0.542 | 0.94 (0.81,1.09) | 0.3916 | -.02  (-.09,0.05) | 0.5471 | -.03  (-.10,0.04) | 0.4103 |
| Intervention*12-Months | 1.13 (0.95,1.34) | 0.1563 | 1.12 (0.95,1.33) | 0.167 | 0.06  (-.02,0.14) | 0.1655 | 0.06  (-.03,0.14) | 0.1725 |
| Intervention*18-Months | 1.19 (0.98,1.45) | 0.0713 | 1.17 (0.96,1.43) | 0.1138 | 0.09  (-.00,0.18) | 0.0616 | 0.08  (-.02,0.17) | 0.103 |
| Baseline CU Proportion who took a malaria test^3^ | 1.57 (1.22,2.01) | 0.001 | 1.52 (1.22,1.90) | 0.0006 | 0.51  (0.26,0.76) | 0.0003 | 0.50  (0.29,0.71) | <.0001 |
| Strata |  |  |  |  |  |  |  |  |
| Webuye W.  (No Health Facility) | 0.91 (0.78,1.07) | 0.25 | 0.96 (0.82,1.12) | 0.5515 | -.04  (-.12,0.03) | 0.2355 | -.02  (-.09,0.05) | 0.4997 |
| Webuye E.  (Health Facility) | 0.99 (0.87,1.13) | 0.8551 | 1.03 (0.92,1.16) | 0.5461 | -.01  (-.07,0.06) | 0.8696 | 0.02  (-.05,0.08) | 0.6035 |
| Kiminini  (No Health Facility) | 0.92 (0.78,1.08) | 0.2975 | 0.91 (0.80,1.04) | 0.1619 | -.04  (-.12,0.04) | 0.295 | -.05  (-.11,0.02) | 0.1851 |
| Webuye E.  (No Health Facility) | 0.99 (0.80,1.23) | 0.9414 | 0.99 (0.83,1.18) | 0.8704 | -.00  (-.10,0.10) | 0.9866 | -.00  (-.09,0.08) | 0.9745 |
| Kiminini (Health Facility) |  | - |  | - |  | - |  | - |
| Wealth Index |  |  |  |  |  |  |  |  |
| 0 - 20th |  | . | 0.77 (0.71,0.85) | <.0001 |  | . | -.13  (-.18,-.09) | <.0001 |
| >20 - 40th |  | . | 0.87 (0.81,0.93) | 0.0002 |  | . | -.08  (-.11,-.04) | <.0001 |
| >40 - 60th |  | . | 0.83 (0.75,0.91) | 0.0002 |  | . | -.10  (-.15,-.05) | <.0001 |
| >60 - 80th |  | . | 0.87 (0.81,0.93) | 0.0002 |  | . | -.08  (-.12,-.04) | 0.0001 |
| 80^th^-100^th^ |  |  | Ref | - |  |  | Ref | - |
| Patient Age |  |  |  |  |  |  |  |  |
| 18+ |  | . | 0.87 (0.81,0.95) | 0.0011 |  | . | -.07  (-.11,-.03) | 0.0012 |
| 5 to 17 |  | . | 0.88 (0.82,0.94) | 0.0002 |  | . | -.07  (-.10,-.03) | 0.0002 |
| 1 to 5 |  |  | Ref | - |  |  | Ref | - |
| Female |  | . | 1.08 (1.02,1.14) | 0.007 |  | . | 0.04  (0.01,0.07) | 0.0033 |
| Male |  |  | Ref | - |  |  | Ref | - |
| Respondent Highest Education level |  |  |  |  |  |  |  |  |
| Completed primary |  | . | 1.03 (0.97,1.09) | 0.3738 |  | . | 0.01  (-.02,0.04) | 0.3792 |
| Completed secondary |  | . | 1.21 (1.13,1.28) | <.0001 |  | . | 0.10  (0.07,0.13) | <.0001 |
| Less than primary |  |  | Ref | - |  |  | Ref | - |

*All regressions are weighted using the following weight calculation: ${weight}_{ik}=\left( \frac{N_{k,total}}{32} \right)/{N_{ik}},$where i=1,…,32 indicates CU and k=1, 2, 3 indicates 6-months, 12-months, and 18-months, respectively.

^1^Between-arm differences expressed as relative risks and risk differences. Relative risk estimated with Modified Poisson regression using generalized estimating equations (GEE) with independence working correlation matrix (clustered at the CU level) and robust standard errors. Risk differences estimated with binomial regression using an identity link function, GEE with independence working correlation and robust standard errors (clustered at the CU level).

^2^ Estimated values correspond to estimation proportions.

^3^ Log baseline proportion for relative risk regressions, raw baseline proportion for risk difference regressions
