# Supplementary material for: Improving rational use of ACTs through diagnosis-dependent subsidies: Evidence from a cluster-randomized controlled trial in western Kenya
Source: PLoS Med. 2018 Jul 17;15(7):e1002607. doi: 10.1371/journal.pmed.1002607 (PMC6049880; doi:10.1371/journal.pmed.1002607)
Supplement: S4 Table — AL, artemether lumefantrine. (DOCX) [file pmed.1002607.s008.docx]

**S4 Table.** Weighted* number (percentage) of ACT users who received a correct dose, an under-dose or an over-dose according to their age.

|  | **6-Months** | | **12-Months** | | **18-Months** | |
| --- | --- | --- | --- | --- | --- | --- |
| **AL dosing** | **Control** | **Intervention** | **Control** | **Intervention** | **Control** | **Intervention** |
| Below dose | 143 (26.4%) | 148 (28.4%) | 161 (27.3%) | 125 (22.6%) | 184 (27.7%) | 148 (21.0%) |
| Correct dose | 344 (63.4%) | 341 (65.5%) | 379 (64.0%) | 379 (68.7%) | 405 (60.9%) | 487 (69.1%) |
| Above dose | 55 (10.2%) | 32 (6.1%) | 51 (8.7%) | 48 (8.6%) | 76 (11.4%) | 70 (9.9%) |
| Missing | 22 (.%) | 20 (.%) | 60 (.%) | 45 (.%) | 19 (.%) | 13 (.%) |
| *All summaries are weighted using the following weight calculation: ${weight}_{ik}=\left( \frac{N_{k,total}}{32} \right)/{N_{ik}},$where i=1,…,32 indicates CU and k=1, 2, 3 indicates 6-months, 12-months, and 18-months, respectively. Note that the denominators in this table (ACT users) are the same as denominators of secondary outcomes of rational ACT use and ACT users with no test. | | | | | | |
