# Supplementary material for: Improving rational use of ACTs through diagnosis-dependent subsidies: Evidence from a cluster-randomized controlled trial in western Kenya
Source: PLoS Med. 2018 Jul 17;15(7):e1002607. doi: 10.1371/journal.pmed.1002607 (PMC6049880; doi:10.1371/journal.pmed.1002607)
Supplement: S5 Table — RD, risk difference. (DOCX) [file pmed.1002607.s009.docx]

**S5 Table**. Weighted* design-adjusted^1^ and weighted design and covariate adjusted^2^ model-estimated between-arm risk differences^3,4^ in ACT use among those who test positive, negative, without a test, and the overall percent who use ACT appropriately^5^ between intervention and control arms. This table provides complementary information to that contained in Table 4 and demonstrates that the linear-binomial and linear-normal approaches to estimating risk differences are comparable for outcomes for which both models converge.

|  |  |  | Linear-Binomial^3^ | | Linear-Normal^4^ | |
| --- | --- | --- | --- | --- | --- | --- |
|  | Sample Proportions | | Unadjusted | Adjusted | Unadjusted | Adjusted |
| *Outcome (denominator)* | *Control* | *Intervention* | *Estimate*  *(95% CI)* | *Estimate (95% CI)* | *Estimate*  *(95% CI)* | *Estimate*  *(95% CI)* |
| **Took ACT after a POSITIVE test** | |  |  |  |  |  |
| Baseline (N=634) | 83.7 | 83.5 |  |  |  |  |
| 6-months (N=602) | 89.7 | 88.5 | -.01 (-.07,0.05) | ** | -.02 (-.07,0.04) | -.02 (-.08,0.04) |
| 12-months (N=628) | 84.4 | 87.9 | 0.03 (-.02,0.09) | ** | 0.03 (-.03,0.08) | 0.02 (-.03,0.07) |
| 18-months (N=782) | 83.8 | 90.0 | 0.05 (-.01,0.11) | ** | 0.05 (-.02,0.13) | 0.05 (-.02,0.12) |
| **Took ACT after a NEGATIVE test** | |  |  |  |  |  |
| Baseline (N=122) | 31.7 | 36.8 |  |  |  |  |
| 6-months (N=179) | 42.4 | 37.7 | -.10 (-.30,0.10) | ** | -.09 (-.29,0.12) | -.10 (-.29,0.10) |
| 12-months (N=128) | 37.8 | 31.9 | -.08 (-.31,0.15) | ** | -.09 (-.35,0.17) | -.08 (-.34,0.18) |
| 18-months (N=90) | 45.6 | 29.9 | -.21 (-.44,0.02) | ** | -.20 (-.44,0.03) | -.20 (-.43,0.03) |
| **Took ACT with NO test** | |  |  |  |  |  |
| Baseline (N=1240) | 62.4 | 61.9 |  |  |  |  |
| 6-months (N=860) | 56.5 | 58.6 | 0.03 (-.06,0.12) | 0.05 (-.04,0.13) | 0.04 (-.05,0.12) | 0.05 (-.03,0.14) |
| 12-months (N=1001) | 69.2 | 61.3 | -.07 (-.14,0.00) | -.06 (-.13,0.01) | -.06 (-.14,0.01) | -.06 (-.13,0.01) |
| 18-months (N=998) | 71.6 | 60.2 | -.10 (-.16,-.04) | -.09 (-.15,-.04) | -.10 (-.16,-.03) | -.09 (-.16,-.03) |
| **TEST ADHERENCE among ALL tested** | | |  |  |  |  |
| Baseline (N=756) | 81.5 | 80.0 |  |  |  |  |
| 6-months (N=781) | 84.8 | 80.3 | -.05 (-.12,0.02) | -.06 (-.12,-.00) | -.04 (-.11,0.02) | -.05 (-.12,0.01) |
| 12-months (N=755) | 82.6 | 83.2 | 0.01 (-.05,0.07) | 0.00 (-.05,0.06) | 0.00 (-.05,0.06) | -.00 (-.06,0.05) |
| 18-months (N=868) | 80.7 | 88.5 | 0.08 (0.02,0.13) | 0.07 (0.02,0.13) | 0.08 (0.01,0.14) | 0.07 (0.01,0.13) |

*All sample proportions and regressions are weighted using the following weight calculation: ${weight}_{ik}=\left( \frac{N_{k,total}}{32} \right)/{N_{ik}},$where i=1,…,32 indicates CU and k=1, 2, 3 indicates 6-months, 12-months, and 18-months, respectively. The N for each time point is the observed total. As a consequence, the sum of weighted numbers may slightly differ from the observed totals.

^1^Design-adjusted model: Adjusts for baseline community unit (CU) level outcome proportion, time indicators for 12- and 18-months, treatment indicator, time x treatment interaction and fixed effects for strata

^2^Design and covariate adjusted model: Adds indicators for wealth quintile, patient age (<5, 5-17, 18+), female gender, and highest level of education of the respondent (none or less than primary, completed primary, completed secondary)

^3^Between-arm differences expressed as risk differences estimated with binomial regression using log-link generalized estimating equations (GEE) with independence working correlation matrix (clustered at the CU level) and robust standard errors. Convergence issues occurred in adjustment models for positives taking ACT and negatives taking ACT, therefore alternative normal linear regression method used to produce estimates and provided for all outcomes for comparison purposes.

^4^Between-arm differences expressed as risk differences estimated with normal linear regression using identity-link GEE with independence working correlation matrix (clustered at the CU level) and robust standard errors. Because the normal distribution model may predict values outside of the [0,1] interval, we report the percentage of predicted values outside this interval. Fortunately, only 3.6% are out of range for “negatives taking ACT” and <1% are out of range for “positives taking ACT”.
