# Supplementary material for: Improving rational use of ACTs through diagnosis-dependent subsidies: Evidence from a cluster-randomized controlled trial in western Kenya
Source: PLoS Med. 2018 Jul 17;15(7):e1002607. doi: 10.1371/journal.pmed.1002607 (PMC6049880; doi:10.1371/journal.pmed.1002607)
Supplement: S6 Table — (DOCX) [file pmed.1002607.s010.docx]

**S6 Table**. Weighted* summaries of antibiotic use of febrile participants and respondents (for participants < 18 years) by arm, follow-up survey time-point, and malaria testing behavior.

|  | **Baseline (N=2017)** | | | **18-Months (N=1927)** | | |
| --- | --- | --- | --- | --- | --- | --- |
| **Variable – Number (%), unless otherwise stated** | **Control** | **Intervention** | **Total** | **Control** | **Intervention** | **Total** |
| **Took Antibiotics**  **(AMONG ALL FEVERS)** |  |  |  |  |  |  |
| No | 675 (67.1%) | 685 (67.9%) | 1360 (67.5%) | 706 (74.0%) | 740 (77.4%) | 1446 (75.7%) |
| Yes | 331 (32.9%) | 324 (32.1%) | 655 (32.5%) | 248 (26.0%) | 217 (22.6%) | 465 (24.3%) |
| Missing | 2 (.%) | 0 (.%) | 2 | 10 (.%) | 6 (.%) | 16 |
| **Took Antibiotics**  **(Positive Malaria Test)** |  |  |  |  |  |  |
| No | 224 (60.2%) | 205 (61.1%) | 429 (60.7%) | 257 (69.8%) | 339 (74.8%) | 596 (72.6%) |
| Yes | 148 (39.8%) | 130 (38.9%) | 279 (39.3%) | 111 (30.2%) | 114 (25.2%) | 225 (27.4%) |
| **Took Antibiotics**  **(Negative Malaria Test)** |  |  |  |  |  |  |
| No | 22 (33.4%) | 32 (38.6%) | 54 (36.3%) | 16 (30.7%) | 35 (52.7%) | 51 (43.2%) |
| Yes | 44 (66.6%) | 50 (61.4%) | 94 (63.7%) | 35 (69.3%) | 31 (47.3%) | 67 (56.8%) |
| **Took Antibiotics**  **(No Malaria Test)** |  |  |  |  |  |  |
| No | 429 (75.8%) | 445 (76.2%) | 874 (76.0%) | 425 (81.4%) | 362 (84.5%) | 787 (82.8%) |
| Yes | 137 (24.2%) | 139 (23.8%) | 276 (24.0%) | 97 (18.6%) | 67 (15.5%) | 164 (17.2%) |

*All sample proportions are weighted using the following weight calculation: ${weight}_{ik}=\left( \frac{N_{k,total}}{32} \right)/{N_{ik}},$where i=1,…,32 indicates CU and k=1, 2, 3 indicates 6-months, 12-months, and 18-months, respectively. The N for each time point is the observed total. As a consequence, the sum of weighted numbers may slightly differ from the observed totals.
