# Supplementary material for: Improving rational use of ACTs through diagnosis-dependent subsidies: Evidence from a cluster-randomized controlled trial in western Kenya
Source: PLoS Med. 2018 Jul 17;15(7):e1002607. doi: 10.1371/journal.pmed.1002607 (PMC6049880; doi:10.1371/journal.pmed.1002607)
Supplement: S8 Table — (DOCX) [file pmed.1002607.s012.docx]

**S8 Table**. Analyses restricted to non-testers and those who had a record observed for their test. Weighted* design-adjusted^1^ and weighted design and covariate adjusted^2^ model-estimated between-arm differences^3^ between intervention and control arms comparing malaria testing behavior, percent of ACT users testing positive, and targeted ACT use^4^. Sample proportions at each time-point, including baseline, are also reported for each outcome.

|  | | | **Relative Risk** | | **Risk Difference** | |
| --- | --- | --- | --- | --- | --- | --- |
|  | **Sample Proportions** | | **Unadjusted** | **Adjusted** | **Unadjusted** | **Adjusted** |
| Outcome | Control | Intervention | Estimate  (95% CI) | Estimate (95% CI) | Estimate  (95% CI) | Estimate (95% CI) |
| **Took malaria test** (AMONG ALL FEVERS) | | | | | | |
| Baseline (N=1622) | 27.9 | 28.2 | - | - | - | - |
| 6-months (N=1158) | 23.4 | 28.1 | 1.20 (0.93,1.55) | 1.22 (0.94,1.58) | 0.05 (-.01,0.11) | 0.06 (-.01,0.12) |
| 12-months (N=1297) | 23.0 | 29.5 | 1.28 (0.99,1.66) | 1.28 (0.98,1.67) | 0.06 (-.00,0.13) | 0.07 (-.00,0.14) |
| 18-months (N=1410) | 24.5 | 39.6 | 1.61 (1.24,2.09) | 1.61 (1.24,2.09) | 0.15 (0.07,0.23) | 0.15 (0.07,0.23) |
| **TARGETED ACT use** (AMONG ALL FEVERS) | | | | | | |
| Baseline (N=1612) | 22.2 | 20.5 | - | - | - | - |
| 6-months (N=1158) | 19.2 | 22.7 | 1.21 (0.86,1.72) | 1.24 (0.86,1.77) | 0.04 (-.03,0.12) | 0.05 (-.03,0.13) |
| 12-months (N=1290) | 18.5 | 23.9 | 1.33 (0.93,1.90) | 1.33 (0.92,1.93) | 0.06 (-.02,0.14) | 0.06 (-.02,0.15) |
| 18-months (N=1397) | 18.6 | 34.2 | 1.88 (1.37,2.60) | 1.89 (1.36,2.64) | 0.16 (0.08,0.24) | 0.16 (0.08,0.24) |
| **Rational ACT use** (AMONG ACT USERS) | | | | | | |
| Baseline (N=1082) | 30.9 | 28.1 | - | - | - | - |
| 6-months (N=704) | 24.4 | 25.0 | 1.02 (0.65,1.60) | 1.03 (0.65,1.61) | 0.01 (-.10,0.13) | 0.01 (-.10,0.12) |
| 12-months (N=888) | 24.2 | 29.1 | 1.20 (0.84,1.71) | 1.20 (0.84,1.71) | 0.05 (-.05,0.15) | 0.05 (-.05,0.14) |
| 18-months (N=1007) | 23.4 | 44.9 | 1.93 (1.43,2.58) | 1.91 (1.41,2.58) | 0.22 (0.13,0.30) | 0.21 (0.13,0.30) |

*All sample proportions and regressions are weighted using the following weight calculation: ${weight}_{ik}=\left( \frac{N_{k,total}}{32} \right)/{N_{ik}},$where i=1,…,32 indicates CU and k=1, 2, 3 indicates 6-months, 12-months, and 18-months, respectively. The N for each time point is the observed total.

^1^Design-adjusted model: Adjusts for baseline community unit (CU) level outcome proportion, time indicators for 12- and 18-months, treatment indicator, time x treatment interaction and fixed effects for strata

^2^Design and covariate adjusted model: Adds indicators for wealth quintile, female gender, and highest level of education of the respondent (none or less than primary, completed primary, completed secondary)

^3^Between group differences expressed as relative risks estimated with Modified Poisson regression using generalized estimating equations (GEE) with independence working correlation matrix (clustered at the CU level) and robust standard errors.

^4^Targeted ACT use defined as taking ACT if positive OR not taking ACT if negative, amongst all febrile individuals. N=9 participants in baseline, N=1 at 12-months, and N=4 at 18-months tested for malaria but had missing information on whether ACT was taken before or after malaria test. N=6 participants at 12-months and N=9 at 18-months had took ACT with conflicting test results. Data on chronology of ACT use was missing for 6-month wave.
